# Mapping subnational gender gaps in internet and mobile adoption using social media data

**DOI:** 10.1073/pnas.2416624122

**Published:** 2025-10-14

**Authors:** Casey F. Breen, Masoomali Fatehkia, Jiani Yan, Xinyi Zhao, Douglas R. Leasure, Ingmar Weber, Ridhi Kashyap

**Affiliations:** ^a^Department of Sociology, University of Oxford, Oxford OX1 1JD, United Kingdom; ^b^Nuffield College, University of Oxford, Oxford OX1 1NF, United Kingdom; ^c^Leverhulme Centre for Demographic Science, University of Oxford, Oxford OX1 1JD, United Kingdom; ^d^Department of Sociology and Population Research Center, University of Texas at Austin, Austin, TX 78712; ^e^Qatar Computing Research Institute, Hamad bin Khalifa University, Doha 00000, Qatar; ^f^Department of Digital and Computational Demography, Max Planck Institute for Demographic Research, Rostock, Mecklenburg-Vorpommern, 18057 Germany; ^g^Saarland Informatics Campus, Saarland University, Saarbrücken 66123, Germany

**Keywords:** digital adoption, sustainable development, machine learning, low- and middle-income countries, gender inequality

## Abstract

The digital revolution has ushered in many societal and economic benefits. Yet access to digital technologies such as mobile phones and internet remains highly unequal, especially by gender in the context of low- and middle-income countries (LMICs). While national-level estimates are increasingly available for many countries, reliable, quantitative estimates of digital gender inequalities at the subnational level are lacking. These estimates, however, are essential for monitoring gaps within countries and implementing targeted interventions within the global sustainable development goals, which emphasize the need to close inequalities both between and within countries. We develop estimates of internet and mobile adoption by gender and digital gender gaps at the subnational level for 2,075 regions in 117 LMICs from 2015 through 2025, a context where digital penetration is low and national-level gender gaps disfavoring women are large. We construct these estimates by applying machine-learning algorithms to Facebook user counts, geospatial data, development indicators, and population composition data. We calibrate and assess the performance of these algorithms using ground-truth data from subnationally representative household survey data from 33 LMICs. Our results reveal striking disparities in access to mobile and internet technologies between and within LMICs. These disparities imply that as of 2025, women are 19% less likely to use the internet and 8% less likely to own a mobile phone in LMICs, corresponding to over 190 million fewer women owning a mobile phone and over 320 million fewer women using the internet.

## Significance

Reliable estimates of digital adoption by gender are essential for tracking progress on global sustainable development targets, such as the United Nations Sustainable Development Goal 5 on gender equality. We construct subnational estimates of internet and mobile adoption by gender, including gender gaps, for 117 low- and middle- income countries from 2015 through 2025. Our estimates show that for women in the least developed countries, as measured by the Human Development Index, more than 40% of total inequality in internet use arises from within-country differences. We validate our estimates and provide accompanying uncertainty estimates. Our estimates provide a valuable resource for researchers and policymakers to monitor digital gender gaps and track progress toward sustainable development goals in real time.

The digital revolution has yielded major societal and economic benefits in low- and middle-income country (LMIC) settings. Internet and mobile technologies are powerful mediums for boosting social connectivity (1, 2), promoting social learning, and providing access to new information channels (3, 4). Increasing digital adoption has generated “digital dividends,” such as job creation (5), better educational outcomes (6), and improved economic growth (7). From a gender perspective, digital technologies have the potential to empower women across many domains and reduce gender inequalities by providing access to information, networks, and vital services that lead to higher contraceptive uptake (8), increased labor market and economic opportunities (9–12), and improved child and maternal health (8, 13–15). The benefits of digital technology are generally greatest in the most unequal, disadvantaged regions (8).

Yet the global spread of digital technologies has been uneven. Over 2.6 billion people have never accessed the internet, and the majority of the unconnected are women and girls (16). This digital divide by gender is an increasingly salient dimension of contemporary population inequality and is especially pronounced in LMICs. Reliable quantitative estimates of digital gender inequalities are essential for tracking progress on and implementing targeted policies and interventions in the context of the global sustainable development goals (SDGs). Reducing inequalities in access to digital technologies by gender is a target within SDG 5 on gender equality, while digital literacy is a core part of SDG 4 on the right to education.

Gender-disaggregated data on digital adoption in LMIC settings are significantly lacking. While the availability of national-level estimates of digital gender gaps has improved (16–18), to date there are no subnational estimates of digital adoption by gender for the majority of LMICs in the world. Past estimates of digital adoption have typically been based on probabilistic household surveys (19, 20), which generally either lack gender disaggregation or are underpowered for subnational analyses. Even when high-quality, subnationally representative data are available, they quickly become outdated in the context of a rapidly evolving technological landscape. In other contexts, such as poverty (21, 22), wealth (23), and population mapping (24), “big data” derived from satellite, social media, and mobile phone records have been used to overcome data gaps in survey-based approaches. The potential of nontraditional sources for mapping gender inequality indicators at subnational geographical resolution has yet to be explored.

Subnational estimates are critical because internet and mobile phone adoption can vary substantially within countries. This mirrors patterns in economic and educational development, which also show high subnational heterogeneity (25). These subnational inequalities in human development are often largest at low levels of human development and also substantial at middle levels. When taking subnational variation into account, inequalities in human development among LMICs are approximately double those when only accounting for national-level variation (25). Moreover, factors associated with digital adoption for women and men can differ, as economic development does not necessarily weaken gender inequalities and facilitate women’s empowerment linearly (26). As development programs are increasingly deployed through digital means, and are often targeted in local geographies, subnational estimates are critical to monitoring their progress and understanding how digital inequalities affect sustainable development.

Here, we introduce an approach to estimating subnational digital adoption and gender gaps by applying machine learning algorithms to social media data, big geospatial data, and development indicators. We focus on internet adoption, defined as having used the internet in the past 12 mo, and mobile phone ownership, defined as having personal ownership of a mobile phone. We train and assess the performance of these algorithms using “ground truth” data from subnationally representative Demographic and Health Surveys (DHS) from 525 regions across 33 LMICs. We use this approach to estimate digital adoption at the first subnational administrative level (admin-1) for 2,075 regions in 117 LMICs where sufficient data are available to make estimates. To facilitate the study of trends in digital adoption and inequality over time, we produce annual estimates from 2015 to 2023, and monthly estimates beginning in 2024 through the present day. These estimates are provided freely alongside this study and will be publicly available and updated on a monthly basis on an interactive web dashboard www.digitalgendergaps.org.

## Results

**Fig. 1.**
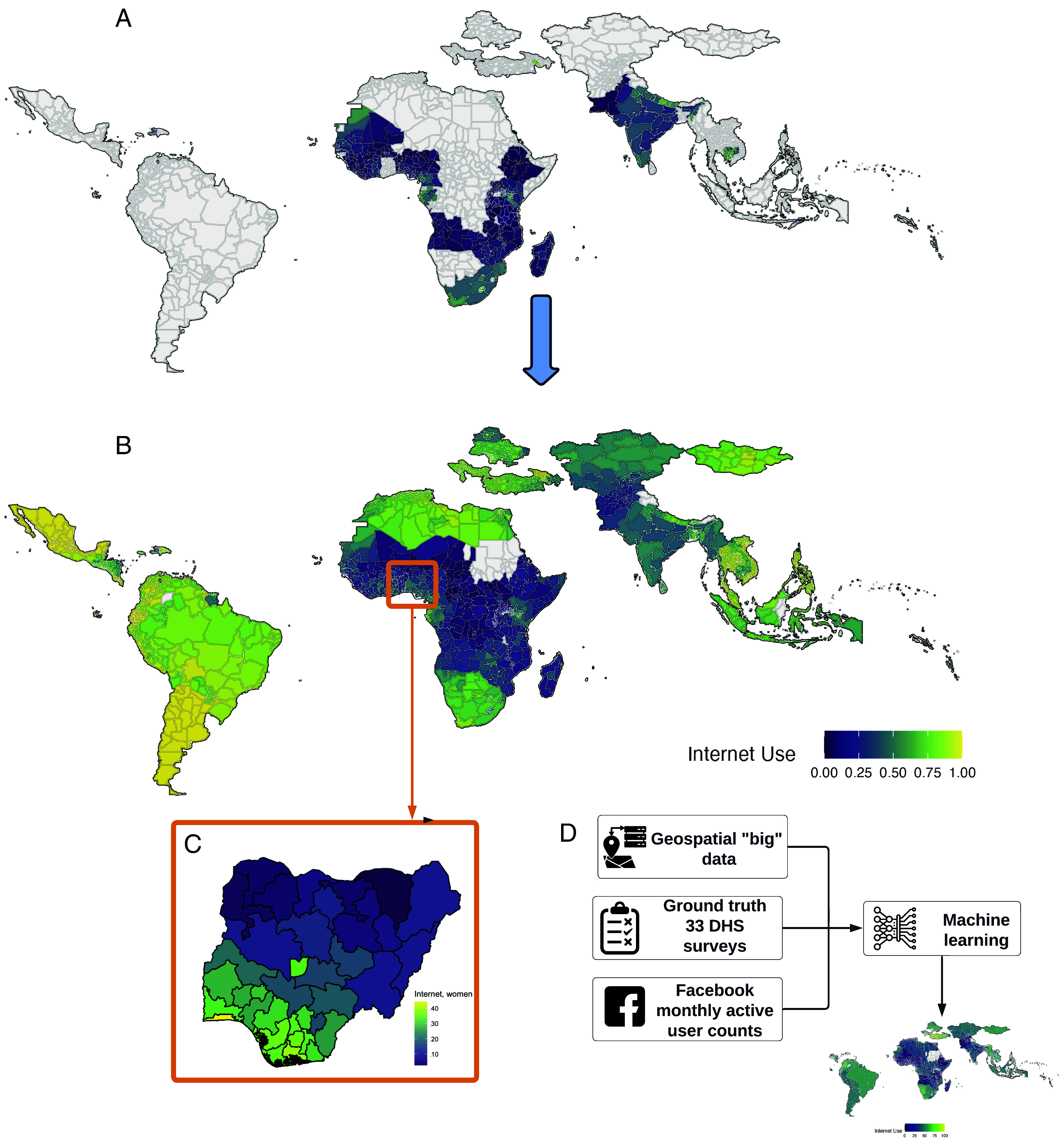
Description of the general approach. (*A*) The 33 countries with available ground truth data. (*B*) Model-based estimates of internet adoption for women across 117 countries for January 2025. (*C*) Enlargement of estimates for Nigeria. (*D*) Illustration of inputs to the machine learning models for predicting digital adoption indicators.

Our general approach is illustrated in Fig. 1. We use survey-based indicators of internet usage and mobile phone ownership for men and women combined with a set of satellite-based geospatial data, development indicators, and social media user count data to train a machine learning model. The social media data we use are Facebook monthly active user counts by gender obtained from the public Facebook Marketing API. The availability of 33 recent DHS surveys (Fig. 1*A*) provides us good coverage of ground truth data on internet and mobile adoption by gender and digital gender gaps to calibrate and assess our models. We temporally align our predictive features to the year of the DHS survey. The near global coverage and more timely, higher-frequency availability of the geospatial and social media features provides a basis for extrapolation to locations without DHS data. Using this approach, we expand our geographical and temporal coverage of digital adoption estimates to 2,075 regions in 117 LMICs across the globe from 2015 to the present.

We focus on LMICs as national-level adoption in these settings is low, digital gender gaps disfavoring women are large (17, 18), and gender-disaggregated data at finer geographical resolution on digital adoption are limited. Our results reveal large disparities in digital adoption between and within countries and demonstrate the promise of this method for real-time monitoring of global SDGs.

### Evaluating Predictive Accuracy.

To assess the predictive accuracy of our machine learning models, we employ three validation strategies: 1) leave-one-country-out cross-validation (LOCO-CV), 2) conventional 10-fold cross-validation, and 3) external benchmarking against independent surveys from the Living Standards Measurement Study (LSMS) and the Multiple Indicator Cluster Survey (MICS) program. Together, these methods help validate our approach and provide evidence of the external validity of our estimates outside of countries where DHS ground truth data are available.

To assess the performance of our estimates using LOCO-CV, we hold out all data from one country at a time. We train our model on ground truth data from all countries not withheld, then make predictions for each subnational unit in the hold-out country. This process is repeated for each country. The resulting estimates give insight into how we would expect the model to perform in countries without ground truth data.

LOCO-CV is a contrasting and more stringent approach to standard 10-fold cross-validation, where one fold (one-tenth) of the dataset is left out at a time instead of an entire country. Leaving out an entire country prevents any data from that country from influencing the training process. The 10-fold cross-validation gives insight into performance in countries where survey data are available in many, but not all areas. In contrast, LOCO-CV imitates a setting where no ground truth data are available for any subnational units in a given country. As over 80 LMICs have no ground truth data on digital adoption at the admin-1 level, we consider this a more conservative and realistic evaluation of model performance.

**Fig. 2.**
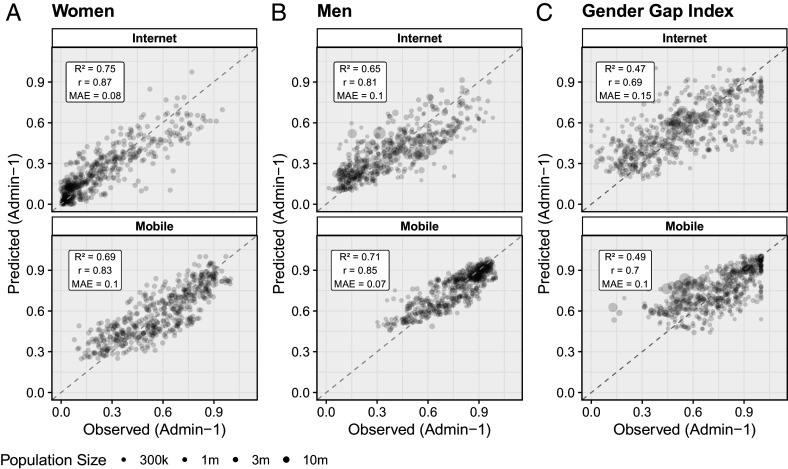
Summary of model performance using leave-one-country-out cross-validation (LOCO-CV). (*A*) Scatterplot of predicted vs. observed subnational values for internet adoption and mobile phone ownership for women. (*B*) Scatterplot of predicted vs. observed subnational values for mobile phone ownership and internet penetration for men. (*C*) Scatterplot of predicted vs. observed subnational values of the gender gap index (female-to-male ratio) for internet and mobile phone adoption. The $R^{2}$ values report the coefficient of determination, the *r* values report the Pearson Correlation Coefficient, and MAE values report mean absolute error.

Fig. 2 plots our model-based predicted values against our observed ground truth values across all subnational units for the different indicators of internet and mobile adoption by gender, and the gender gap indices (female-to-male ratio) of both. Maps visualizing predicted values of all indicators are shown in *SI Appendix*, section 4. To quantify the agreement between our predictions and ground truth, we report the coefficient of determination ($R^{2}$), the Pearson correlation coefficient (*r*), and the mean absolute error (MAE). The coefficient of determination ($R^{2}$) represents the proportion of total variation in the dependent variable that is predictable from the independent variable, and the MAE reports the average absolute difference between the predicted and observed values.

Our predictions align closely with the ground truth where these are available. On average, our predictions are better for women’s internet adoption than men’s. Our predictive accuracy is lower for our estimates of gender gaps. This likely reflects a combination of more noise in the underlying ground truth data (for more details, see *SI Appendix*, section 3C) and overall weaker relationship between our predictive features and digital gender gaps compared to digital adoption levels (*SI Appendix*, Fig. S32). In addition to material gender inequalities, which we are better able to measure in our feature set through indicators linked to income and education, gender gaps may reflect social norms, which are generally more challenging to measure in these settings.

As an additional validation exercise, we benchmark our estimates against the LSMS surveys—high-quality, subnationally representative surveys fielded by the World Bank. Despite using slightly different definitions of digital adoption than the DHS, our estimates show strong agreement with LSMS estimates at the admin-1 level (*SI Appendix*, Fig. S3). We also benchmark against MICS surveys at the admin-1 level (*SI Appendix*, Fig. S4), finding close alignment even in high-adoption settings in Central and South America.

Through temporally aligning our features with the year of the DHS, the model learns from both spatial and temporal variation. Even though the DHS are fielded at different time points, our models predict accurately across the span of 2015 to 2022 (see *SI Appendix*, section 3F for details). This suggests that our model generalizes well across time and space. Direct validation of temporal trends is constrained by data availability and differences in survey design, question wording, and sample sizes across surveys. Despite these constraints, we conduct analyses using DHS, MICS, and LSMS surveys for the limited number of countries where multiple waves are available. As shown in *SI Appendix*, Fig. S10, the correlations between our predicted and survey-based estimates of change over time range from 0.21 to 0.38. We report these correlations to assess alignment despite known limitations, but caution against overinterpretation: Subnational estimates of multiyear change from different surveys are subject to substantial noise. Low to moderate correlation is expected and does not necessarily reflect the models’ inability to capture trends.

The variance of the predicted changes in adoption over time shown in *SI Appendix*, Fig. S10 is far more attenuated than the variance of survey-based estimates of changes in adoption over time. Further, survey-based estimates show declines in adoption over time for some subnational units that our models do not predict. Given current limitations in the availability of consistent surveys over time and small sample sizes, it is difficult to ascertain to what extent these differences reflect model performance or inconsistencies in survey-based estimates arising from differing definitions and sampling variability.

For all estimates, we calculate a corresponding estimate of model error (*SI Appendix*, section 3B). We do this by regressing the absolute value of the residual (model error) against the set of all observable features, following (23). This model is then used to calculate an estimate of model error for each subnational unit. Our predicted model error is smallest in low-adoption settings, indicating our approach is able to identify areas that are lagging behind.

### Within-Country Results.

**Fig. 3.**
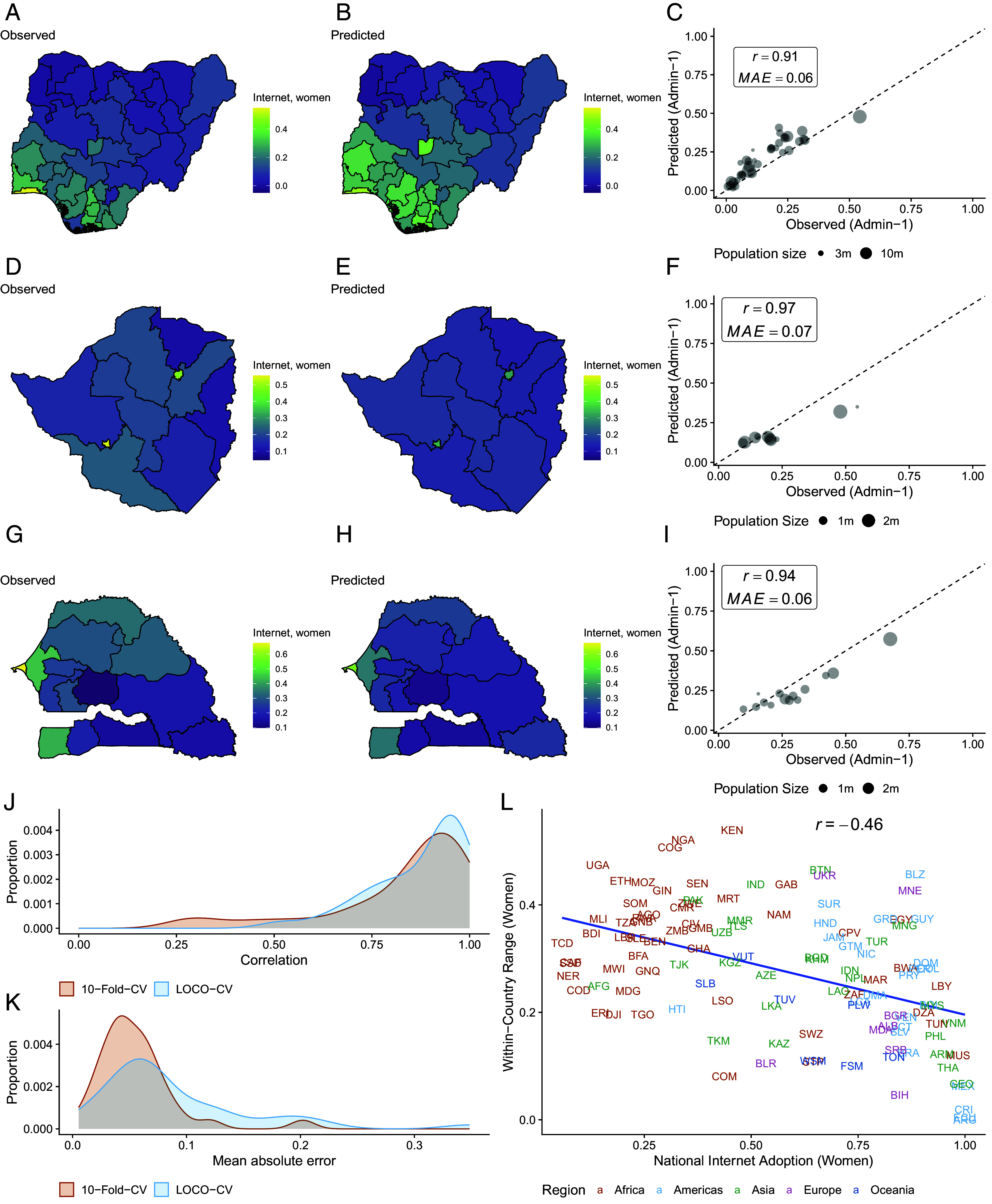
Performance of model predicting internet use among women, assessed using leave-one-country-out cross-validation (LOCO-CV). (*A*–*C*) Observed and predicted estimates of internet adoption for women for Nigeria. (*D*–*F*) Observed and predicted estimates of internet adoption for women for Zimbabwe. (*G*–*I*) Observed and predicted estimates of internet adoption for women for Senegal. (*J* and *K*) Distribution of within-country mean absolute error (MAE) and correlation between observed and predicted values for both standard 10-fold CV and LOCO-CV. (*L*) Relationship between national-level estimates and within-country range (difference between *Top* and *Bottom* subnational units) for female internet penetration.

Fig. 3 illustrates within-country variation in women’s internet adoption using three examples from the African continent. These countries exhibit low overall internet penetration rates, substantial heterogeneity across admin-1 units, and ongoing collective efforts to enhance internet accessibility and quality (e.g., the Zimbabwe National Broadband Plan). Reliable and timely estimates of digital adoption are crucial for assessing the impact and effectiveness of these initiatives.

In Nigeria, 55% of women in the relatively affluent and urban southwestern state of Lagos had accessed the internet in the past 12 mo, while less than 1% of women had accessed the internet in the rural northern state of Kebbi as of 2018 (Fig. 3*A*), highlighting the magnitude of within-country heterogeneity. Within Nigeria, the model-based estimates align closely with the observed DHS ground truth (Fig. 3*B*).

For Zimbabwe (Fig. 3*D*), our model is able to accurately estimate internet adoption in the small geographic provinces of Harare and Bulawayo, which have nearly triple the rate of internet penetration of neighboring regions. In Senegal (Fig. 3*G*), our estimates slightly underpredict overall internet penetration, but closely capture the overall pattern of adoption. Fig. 3*J* shows the within-country distribution of the Pearson Correlation Coefficient (*r*) and Fig. 3*K* shows the within-country distribution of mean absolute error between our predictions and ground truth using both standard 10-fold cross-validation and LOCO-CV. After demeaning the values by country and pooling all subnational units, correlations between observed and predicted values range from 0.49 to 0.81 (*SI Appendix*, Fig. S34). These correlations reflect the models’ ability to capture subnational variation within countries in digital adoption and gender gaps. For a comparison of our model-based estimates and ground truth for each country, see *SI Appendix*, Fig. S26. Finally, Fig. 3*L* shows the within-country range in internet adoption among women at different levels of national adoption for our January 2025 estimates, indicating that within-country variation is generally larger at lower levels of adoption. This pattern is consistent across other adoption indicators (*SI Appendix*, Fig. S29).

**Fig. 4.**
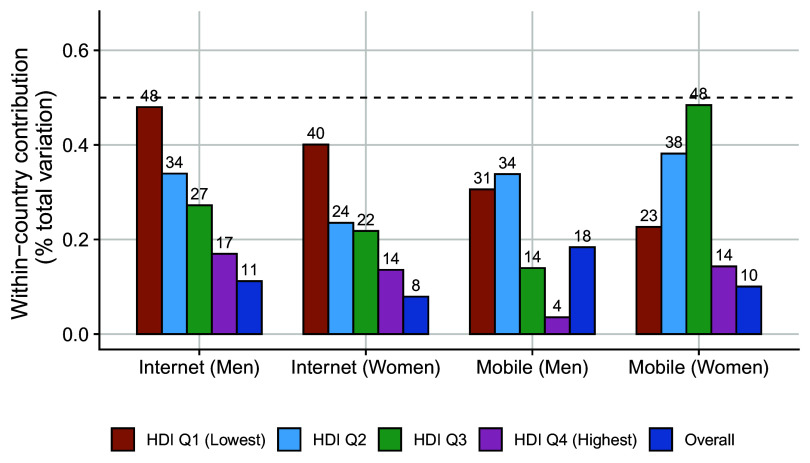
The relative contribution of within-country variation in internet and mobile adoption to total variation by human development index (HDI) quantile. The dashed line represents equal contribution of within-country and between-country variation to overall inequality.

Fig. 4 shows the proportion contribution of within-country inequality in internet and mobile adoption to total inequality within different human development index (HDI) quantiles in the distribution of 117 focal LMICs, based on January 2025 estimates (further details described in *SI Appendix*, section 3D). Subnational inequality accounts for a large proportion of the overall variation in adoption, especially at lower levels of human development. For internet adoption in countries with the lowest levels of human development, the within-country inequality accounts for 40% (women) and 48% (men) of the overall inequality.

**Fig. 5.**
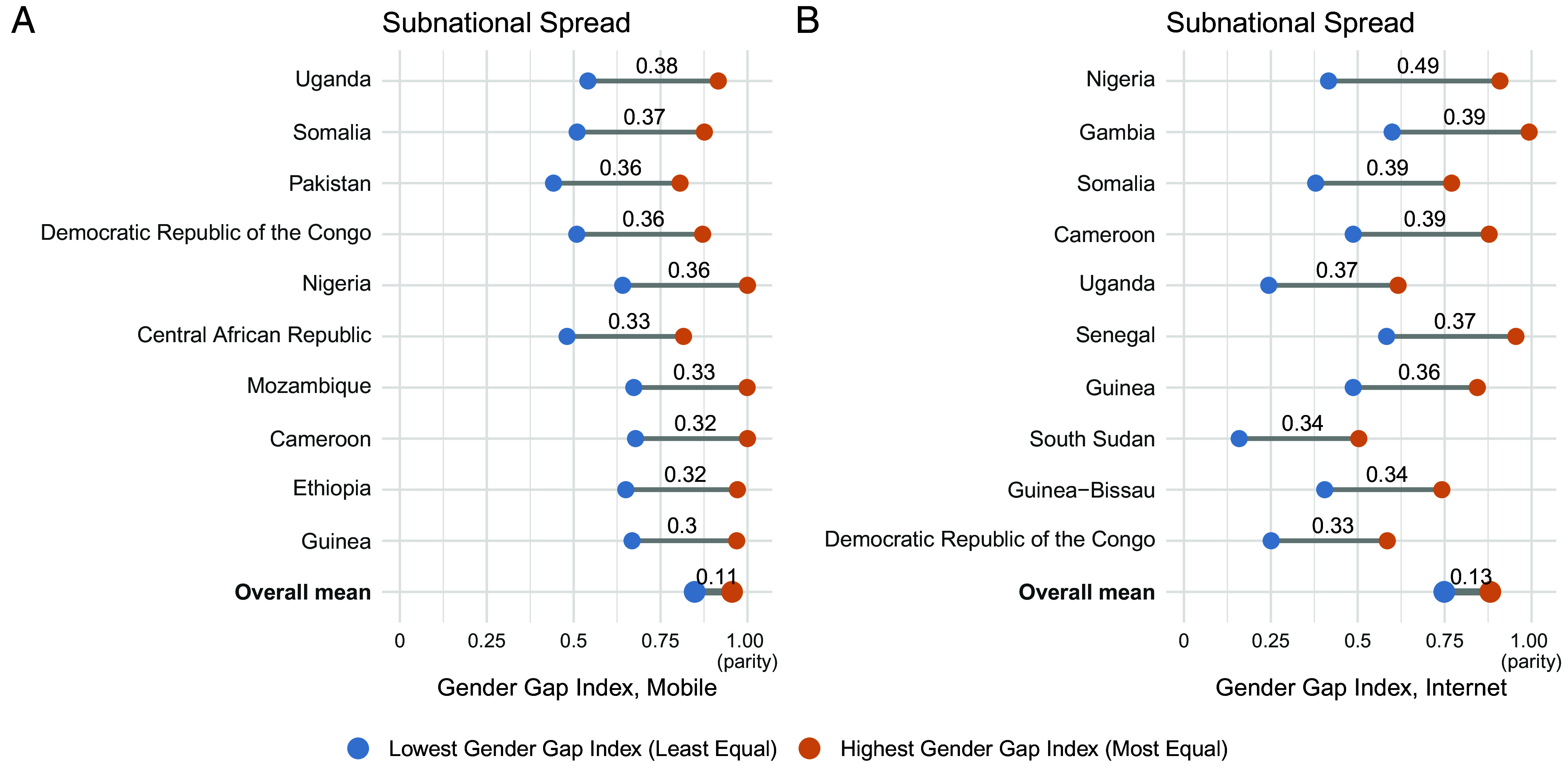
The top 10 countries with the largest spread between their lowest and highest subnational unit with respect to the digital gender gap index (female-to-male ratio), organized in descending order by spread size for mobile (*A*) and internet (*B*). The *Bottom* bar shows the average *Top-Bottom*subnational spread across all countries.

Our estimates highlight substantial disparities in digital adoption and gender gaps between the *Top* and *Bottom* subnational units. Fig. 5 illustrates the countries with the largest subnational disparities in internet and mobile gender gaps as of January 2025. The *Bottom* bar shows the average *Top*-*Bottom* subnational disparity across all countries, which for the internet gender gap is 0.13 and mobile gender gap 0.11. In Nigeria, for example, the country with the largest subnational disparity in the internet gender gap index, the gender gap between the region closest to gender parity (Lagos, 0.92) and the region farthest from gender parity (Katsina, 0.43) is 0.49. The largest within-country disparities for internet and mobile adoption levels are shown in *SI Appendix*, Fig. S20. Overall, internet adoption exhibits larger subnational disparities than mobile adoption. Across all digital indicators of adoption levels and gender gaps, the largest subnational disparities are concentrated in Africa, where historically uneven development, ethno-cultural differences, and investment efforts have resulted in highly disparate subnational regions (27). Outside of Africa, large subnational disparities occur in India and Pakistan.

### Trends in Digital Adoption and Gender Gaps.

**Fig. 6.**
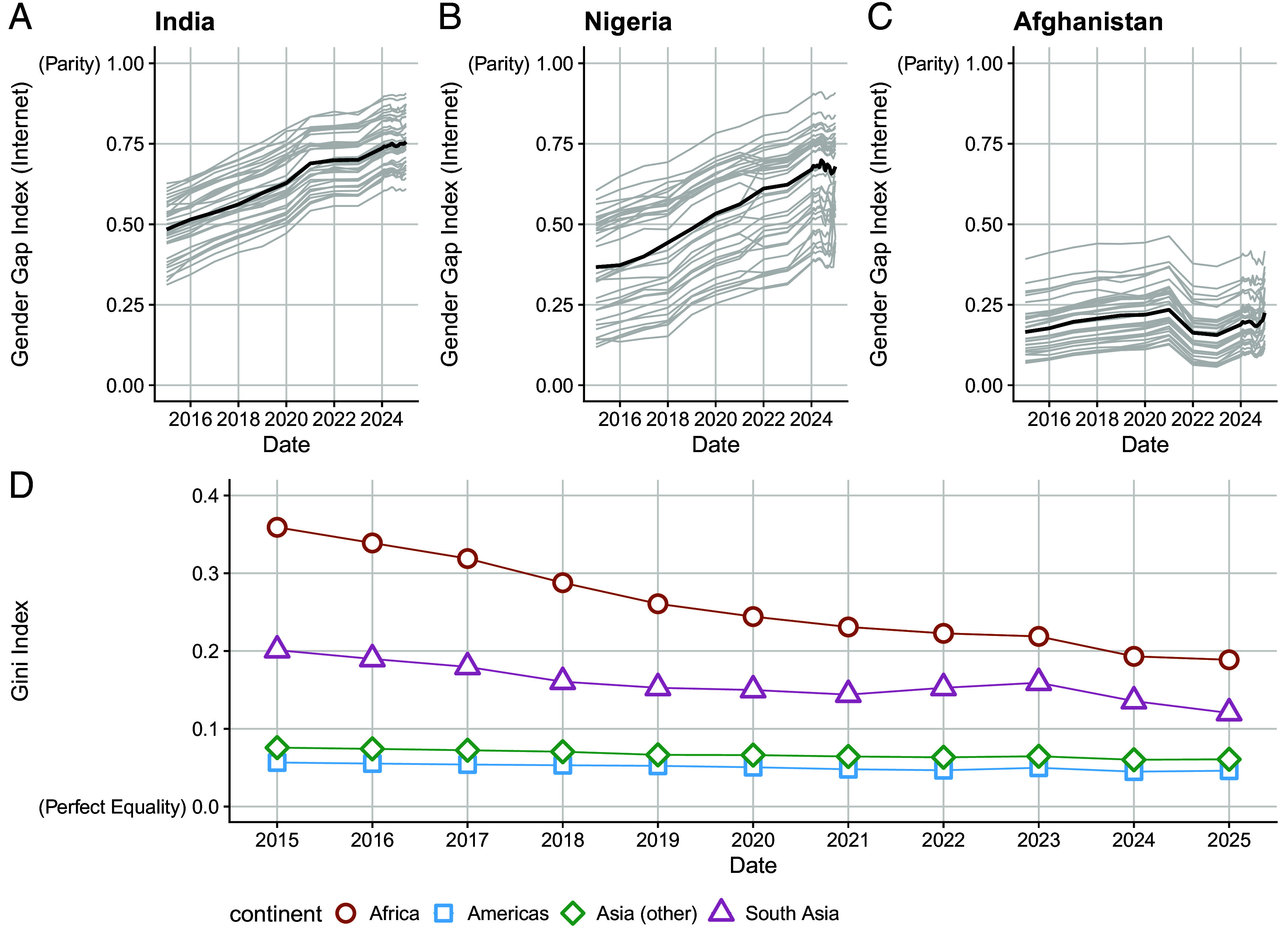
(*A*–*C*) Trends over time in India, Nigeria, and Afghanistan for the internet gender gap index (female to male ratio). The light gray lines show subnational change over time, and the dark shaded line shows the median trend for each country. (*D*) Change in within-country inequality for the internet gender gap, as measured by the subnational GINI index.

For each indicator, we produce annual estimates from 2015 to 2023 and monthly estimates beginning January, 2024 through the present. Based on this, we can assess how average within-country inequality in internet adoption has changed over time. Fig. 6*D* shows the relative within-country inequality in the gender gap as defined by the GINI index by continent. The GINI index is a widely used measure of inequality ranging from 0 to 1, with higher values corresponding to higher levels of inequality. Over time, inequality in subnational units within countries has been declining. In particular, much progress has been made in Africa, with inequality within subnational units declining between 2015 and 2025. Progress has been made in Asia and the Americas over time, but from lower starting levels of within-country inequality. Much of this is explained by increasing adoption: As countries move toward near universal adoption in all subnational units, inequality declines.

Fig. 6 shows trends over time in the internet gender gap index across three example countries with contrasting trends. Both Nigeria and India have made substantial progress in closing the gender gap across all of their subnational units, as indicated by the increasing female-to-male ratio of internet use. Yet meaningful gender gaps remain in both countries, and progress is not universal. Afghanistan, for example, has faced stalled progress, and gender inequality with respect to internet adoption has increased, primarily due to stalled internet adoption for women, as revealed by the worsening female-to-male ratio. The model is able to detect this decrease in internet adoption for women beginning in 2021, which coincides with the year of the Taliban’s Offensive and return to power in the country.

### Feature Sets.

**Fig. 7.**
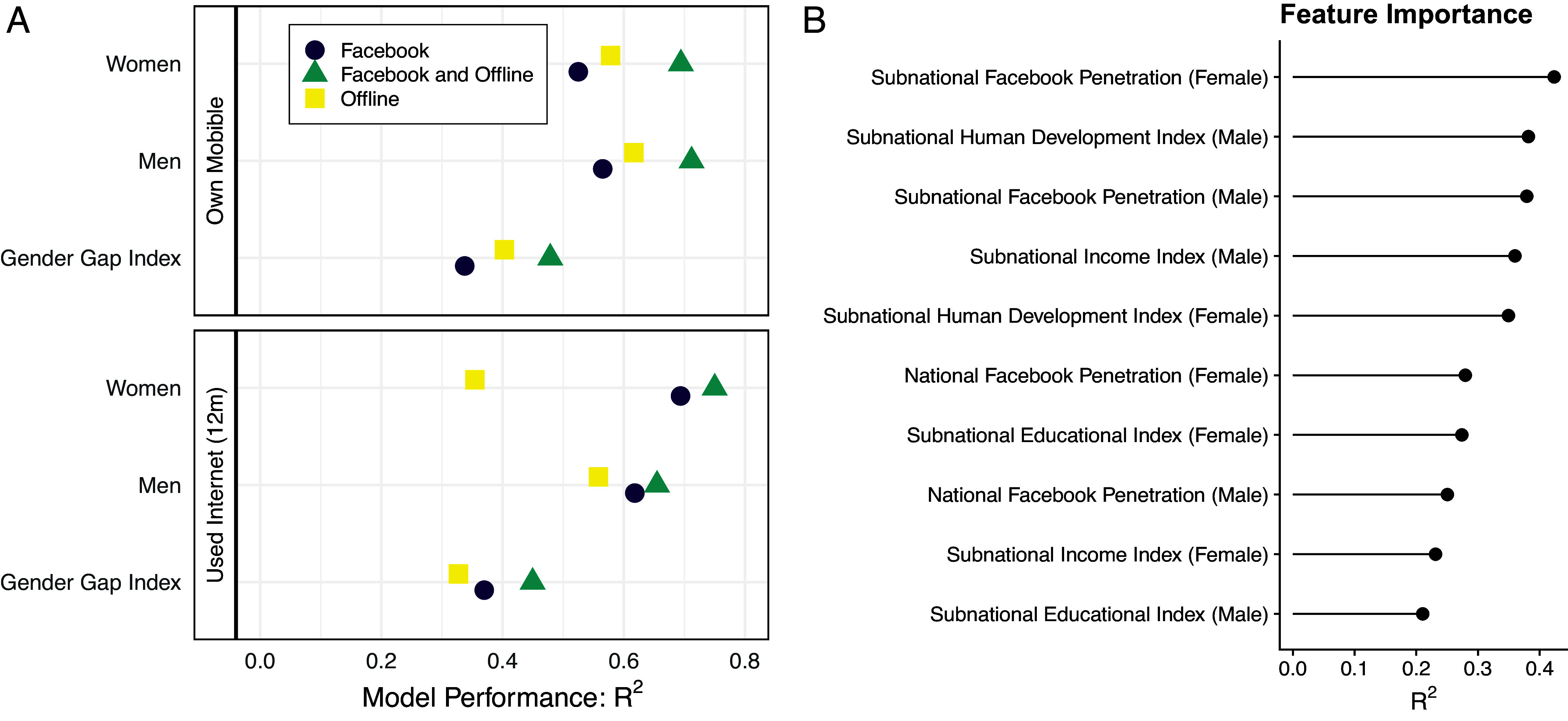
Model performance and feature importance. (*A*) The $R^{2}$ values from leave-one-country-out cross-validation (LOCO-CV) using Facebook predictors, offline predictors, and both Facebook and offline predictors. (*B*) The 10 top features with strongest predictive power for internet penetration for women, as measured by $R^{2}$ for a univariate regression.

Next, we compare the performance of models trained on different feature sets to give insight into the most important features for our models. We test three sets of features: features constructed from Facebook monthly active user counts (“Facebook features”), features derived from geospatial, satellite, and population data (“offline features”), and our full set of features. Fig. 7*A* shows the $R^{2}$ value (coefficient of determination) based on models using each different set of features and LOCO-CV.

Several insights emerge from this figure. First, for mobile phone indicators, models trained using only Facebook features performed the worst, while for internet indicators, models trained using only offline features had the poorest performance. In general, Facebook features are stronger predictors of internet use than mobile phone use, likely due to the direct relationship between internet and Facebook usage. Second, including Facebook features substantially improves model accuracy. The increase in $R^{2}$ value was smallest for the mobile gender gap (0.07) and largest for internet adoption for women (0.40) between the offline model and the combined model with Facebook and offline features. This suggests that combining Facebook and offline features is a promising approach for estimating digital adoption and gender gaps, particularly for internet outcomes. Finally, offline features are stronger predictors of the mobile gender gap, whereas Facebook features better predict the internet gender gap. Offline predictors only modestly improved the performance of the model predicting gender gaps in internet adoption, suggesting that offline predictors may proxy for access and adoption, but do not as effectively capture the relative disadvantage of women compared with men in internet access.

Fig. 7*B* shows the most important features in our model predicting internet adoption for women; *SI Appendix*, Fig. S32 shows the most important features for all indicators. Given the number of highly correlated predictors (e.g., Facebook penetration for men and Facebook penetration for women), we calculate feature importance as the $R^{2}$ for a univariate regression, assessed using LOCO-CV. These results show the factors that are most strongly associated with digital adoption.

The subnational Facebook features, along with proxies of overall economic development and human development at the subnational level, are the best predictors of overall internet adoption for both men and women. Other important features include the subnational education index and national-level Facebook penetration features. The importance of the human development index and its components is consistent with prior research modeling digital gender gaps at the national level (17, 18). While subnational income indices of the HDI are stronger predictors of gender-specific levels of adoption, the educational, human development, and gender development indices are more important for predicting gender gaps. This suggests that while digital adoption levels reflect overall economic development, digital gender gaps are more closely tied to levels of educational attainment and gender equality.

## Discussion

Access to digital technology is an increasingly important dimension of population inequality, and acknowledged as a key indicator within the global SDGs, specifically SDG 4 on education, SDG 5 on gender equality and women’s empowerment, and SDG 17 on revitalizing global partnership for sustainable development (*SI Appendix*, section 2). However, consistent and reliable subnational estimates of internet or mobile adoption are lacking, particularly in LMICs. Here, we demonstrate an approach for estimating subnational levels of internet adoption and mobile phone ownership by gender by applying machine learning algorithms to Facebook user counts, geospatial data, development indicators, and population composition data. Our approach enables us to expand subnational estimates from 525 regions in 33 LMICs, for which DHS ground truth is available, to 2,075 regions in 117 LMICs from 2015 to 2025.

Our results highlight the importance of focusing on the subnational context. In over 40 countries, the gap between the highest and lowest subnational units with respect to female internet adoption exceeds 30 percentage points, with an average gap of 28 percentage points. This subnational variation is especially pronounced in countries with very low levels of human development, where within-country inequality can account for over 40% of total inequality. This underscores the importance of subnational resolution for a more complete understanding of inequality in digital adoption, especially in countries with the lowest overall levels of human development. Our estimates provide a valuable lens for researchers and policy makers through which to assess areas that stand to benefit from the increasing rollout of digital programs and services within global development policies, as well as those at risk of being left behind. Similarly our method enables us and others to track how digital inequalities are shaped by external sociopolitical events, as indicated by the Afghanistan case where our model is able to detect a decrease in women’s internet adoption after 2021 with the resurgence of the Taliban.

There are several promising avenues for further research to address some of our study’s limitations. First, while Facebook is currently the world’s largest social media platform, and is especially dominant in many LMICs, its popularity may decline in the future. This approach could be expanded to include social media data from additional platforms, though the ability to do so is contingent on public access to platform user count data. Second, our estimates are at the first administrative level. This represents a large improvement over national-level estimates, but some potential policy use cases may demand more geographic granularity. If reliable digital adoption estimates and social media user count data become available at the admin-2 level, our methods can be extended to a finer geographic resolution. Third, our training data are limited geographically, especially in Central and South America. However, our models perform well when compared to external survey-based estimates from the LSMS, indicating strong external validity on unseen data (*SI Appendix*, Fig. S3). Further, our estimates demonstrate strong and consistent correlations with independent data from the MICS, including in countries in Central and South America (*SI Appendix*, Fig. S6). Finally, while we provide systematic subnational predictions of trends of digital adoption over time, rigorous validation is limited by data availability. We recommend caution in interpreting unit-level temporal changes. We call upon more data collection efforts within global surveys to monitor digital inequalities at subnational levels.

Our approach addresses the call to usher in a “data revolution for sustainable development” by integrating social media, geospatial, and population data to monitor progress on the SDGs with better geographical and temporal resolution (28). We contribute to a growing body of work that emphasizes how machine learning approaches together with nontraditional sources of data can provide large-scale, cost-effective, and complementary measurement approaches to survey and field-based approaches for SDG monitoring (17, 24) and poverty and economic inequality mapping (22, 23, 29, 30). We show how these innovations can be applied to map gender-disaggregated indicators at finer geographical resolution, which is crucial for reducing inequalities both within- and between-countries. While reliable ground truth data are essential for training our models, the incorporation of higher-frequency social media and geospatial data enables us to provide a contemporary estimate of digital gender gaps. Moving forward, our continuous Facebook collections and regular updates will allow us track progress on digital gender inequalities across all LMICs with a monthly resolution (“nowcasting”). Alongside the geographical and temporal breadth, this subnational pipeline can also be usefully applied to assess the impacts of sociopolitical changes, policies, and interventions over time in specific cases and contexts.

## Materials and Methods

To produce our estimates, we use three main sources of data: ground truth from DHS, offline features, and Facebook features. Our Facebook features are constructed from the Facebook monthly active user counts obtained from the Facebook Marketing API. Our offline features are constructed from population density data, satellite imagery, and subnational indices on human development, education, and income. No ethical approval was required for the study as we use entirely secondary data sources in the public domain that are preaggregated or aggregated from anonymized datasets. To train and calibrate our models, we use ground-truth data on internet use and mobile phone ownership from DHS surveys in 33 LMICs. A full list of features and ground truth outcomes is shown in *SI Appendix*, Table S1 and steps outlining feature construction are provided in *SI Appendix*, section 1.

### Ground Truth Data on Internet and Mobile Access.

Our ground-truth data come from 33 DHS surveys conducted between 2015 and 2023 covering 525 subnational units (31, 32). We include surveys from DHS Phase 7 onward, when questions on digital adoption were first added to the DHS questionnaire. There are several reasons DHS surveys are an excellent source of ground truth data for measuring digital adoption. First, DHS surveys are subnationally representative at the admin-1 level and have a well-established and vetted survey design. Second, unlike many censuses which ask about digital adoption at the household level, DHS surveys collect information at the individual level about digital adoption for both men and women. The individual-level data allow us to calculate gender-specific rates of digital adoption for internet and mobile technologies. Finally, across countries, the DHS program uses harmonized survey questions and sampling design, allowing for cross-national comparisons.

We use DHS microdata to obtain estimates of the percent of men and women aged 15 to 49 who own a mobile phone and have accessed the internet. Internet adoption is defined as having used the internet in the past 12 mo, and mobile phone ownership is defined as having personal ownership of a mobile phone. We focus on internet use in the past 12 mo, rather than having ever used the internet, because we are interested in measuring how many people have reliable access to the internet. We also calculate the gender gap index, defined as

[1] $$\begin{array}{c} \text{Gender Gap Index}=\frac{{\text{I}}_{f}/{\text{I}}_{m}}{{\text{Pop}}_{f}/{\text{Pop}}_{m}}, \end{array}$$

where for a specific indicator *I* (e.g., mobile phone ownership or internet use in the past 12 mo), ${\text{I}}_{f}$ is the number of female users aged 15 to 49, ${\text{I}}_{m}$ is the number of male users aged 15 to 49, ${\text{Pop}}_{f}$ is the total population of women aged 15 to 49, and ${\text{Pop}}_{m}$ is the total male population aged 15 to 49.

### Facebook Monthly Active Users.

To obtain counts of Facebook monthly active users (MAU), we query the public Facebook Marketing API. The Facebook Marketing API provides estimates of the number of daily or monthly active users disaggregated by characteristics such as gender, age, and device type (e.g., Android, iOS) within a given geographic boundary. To query the Facebook Marketing API, we use the pysocialwatcher package (33), and collect counts of monthly active users by gender and device type at the admin-1 level.^*^

We use these MAU counts to construct several different Facebook features. Our primary features are Facebook penetration by gender, defined as the proportion of women (or men) aged 18+ who used Facebook in the past month within a given admin-1 unit. Additionally, we create features corresponding to both the gender-specific gaps (female-to-male ratios) and the fraction of users who accessed Facebook through different access devices (e.g., iOS device). Finally, we include three national-level Facebook features on adoption by gender and gender gaps. For the full set of Facebook features used in our models, see *SI Appendix*, Table S1.

### Temporal Alignment of Features with Ground Truth.

The DHS data we use spanned the years 2015–2023, a period under which digital adoption increased. To the extent possible, we temporally aligned our features with the year of our ground truth observations as closely as possible. For instance, to align our features temporally with ground truth from the 2018 Nigeria DHS, we calculate the population-weighted nightlights feature for Nigeria using nightlights data from 2018 and population data from 2018. When perfect temporal agreement between feature and observed ground truth is not feasible, we used the closest available year to the DHS survey year. For our Facebook features, we constructed national-level features from our regular data collections spanning 2019–2025. Since the Facebook Marketing API cannot retrieve historical MAU counts, we linearly imputed national MAU counts back to 2015 to align temporally with the year of our ground truth DHS surveys. Our subnational Facebook features are based on ongoing collections beginning in April 2024. To achieve temporal alignment, we rescaled the 2024 subnational MAU counts using an adjustment factor, calculated as the ratio of the national MAU counts for the year of interest to the national MAU counts in 2024 (see *SI Appendix*, section 1 for details). To further capture changes over time in our models, we include a feature corresponding to the relative year a DHS survey was conducted.

### Machine Learning Approach.

We use a machine learning approach for prediction. We predict each of the six indicators separately using both Facebook and offline features. Flexible machine learning algorithms are appealing in this setting because of their ability to detect interactions, model higher-order effects, and better handle multiple, highly correlated predictors (34). Machine learning approaches have been applied for similar prediction settings for LMICs, such as for small-area estimation of wealth and poverty (21, 23).

We use ensemble Superlearning—also known as weighted ensembling or stacking—a method for combining multiple machine learning algorithms into a single algorithm (35). The motivation behind ensemble Superlearning is that a weighted combination of different algorithms may outperform any single algorithm by smoothing out limitations of any specific algorithm. The ensemble Superlearner algorithm selects the best weighted combination of algorithms using a cross-validation procedure to minimize overfitting risk (36). For our ensemble Superlearner, we use a library of widely used machine learning algorithms: random forests, generalized linear regression, gradient boosting machines, lasso regression, elastic net regression, polynomial splines regression, ridge regression, and extreme gradient boosting machines (see *SI Appendix*, Table S4 for a description of each algorithm and its weight toward the final ensemble Superlearner algorithm).

In total, we model six different outcomes: adoption levels by gender and gender gaps for both internet penetration and mobile phone ownership. We also generate an accompanying estimate of uncertainty for each prediction (*SI Appendix*, section 3B). For more detailed technical information on our machine learning approach, see our completed REFORMS checklist (37), a resource for promoting transparency and reproducibility in machine learning science.

### Estimating Trends.

Using the temporally aligned features, we trained models to predict digital adoption and gender gaps annually from 2015 to 2023 and monthly from January 2024 onward. Temporal variation was captured through year-specific features and the inclusion of a relative-year covariate. Because our training data spans a wide time range and includes temporal alignment of predictors, the model learns both spatial and temporal patterns in digital adoption. This enables us to generate consistent time series of subnational estimates, which we use to assess trends in adoption levels, gender gaps, and within-country inequality over time. However, estimating and validating trends remains inherently challenging due to limited temporal coverage of ground truth surveys and the lack of standardized validation data across years.

### Cross-Validation.

To evaluate the performance of our model, we use both standard 10-fold cross-validation (10-fold CV) and leave-one-country-out cross-validation (LOCO-CV). For 10-fold CV, we randomly split our sample into ten separate folds. We trained our models on ninefolds and made predictions on a single hold-out fold; we repeated this process for each fold. We use the predictions on all held-out folds to estimate several model performance metrics.

For LOCO-CV, we split the sample into 33 separate folds defined by country. Holding out all subnational units in a given country (“hold-out partition”), we fit our models on the rest of our dataset (“training partition”). We then use our models to predict on the held-out subnational units of that country. This process is iterated for each country in the dataset, ensuring that every country’s subnational units serve as a hold-out set. We use the predictions on all held-out units to estimate model performance metrics. By holding out data from a single country during training, LOCO-CV tests the model’s capability to handle intercountry variability and minimizes overfitting risks specific to individual countries. LOCO-CV addresses concerns of geographical independence, providing a more stringent assessment of the model’s geographical robustness.

We use these two separate cross-validation designs as they provide different perspectives. The LOCO-CV imitates a setting where we have no ground truth data for an entire country, while standard cross-validation is helpful for approximating how our model would perform in settings where ground truth data is available for most, but not all, subnational regions in a country. In comparison to 10-fold CV, LOCO-CV predictions show more conservative estimates of model performance (*SI Appendix*, Fig. S21).

### External Validation.

To assess external validity and generalizability over time, we benchmarked our predictions against 21 LSMS surveys (38) and 24 MICS surveys (39, 40), none of which were used in model training. Despite differences in survey design, reference periods, and question wording, our estimates aligned closely with observed values at the admin-1 level, showing high correlations and low mean absolute errors. To evaluate each model’s ability to capture temporal dynamics, we compare predicted and survey-based changes in digital adoption using repeated surveys using DHS, MICS, and LSMS within countries. Despite measurement limitations, the results indicate that the model captures meaningful variation in change over time. Nonetheless, we recommend caution when interpreting year-to-year changes for individual admin-1 units, as our ability to rigorously validate these estimates is limited. For a more detailed discussion and validation of trends, see *SI Appendix*, section 3F.

### Performance Metrics.

We use several different model performance metrics to evaluate model performance. First, we use $R^{2}$, the coefficient of determination. Given a set of observed values $\left\{y_{1},y_{2},\ldots ,y_{n}\right\}$ and a set of predicted values $\left\{{\hat{y}}_{1},{\hat{y}}_{2},\ldots ,{\hat{y}}_{n}\right\}$, the $R^{2}$ value is defined as

[2] $$\begin{array}{c} R^{2}=1-\frac{\sum_{i=1}^{n}{\left(y_{i}-{\hat{y}}_{i}\right)}^{2}}{\sum_{i=1}^{n}{\left(y_{i}-\overset{¯}{y}\right)}^{2}}, \end{array}$$

where $y_{i}$ is the observed value for the $i^{\mathrm{th}}$ observation, ${\hat{y}}_{i}$ is the predicted value for the $i^{\mathrm{th}}$ observation, $\overset{¯}{y}$ is the mean of the observed values, and *n* is the total number of observations. The $R^{2}$ value, or coefficient of determination, quantifies the proportion of variance in the dependent variable explained by the model. An $R^{2}$ value of 0 means the predictions are no better than using the mean of the outcome, whereas a value of 1 signifies perfect predictions.

As an alternative metric for assessing model fit, we use MAE:

[3] $$\begin{array}{c} MAE=\frac{1}{n}\sum_{i=1}^{n}\left|y_{i}-{\hat{y}}_{i}\right|. \end{array}$$

The MAE provides an absolute measure of the average prediction error in the dependent variable’s units, with a lower MAE indicating better model accuracy. Using both $R^{2}$ and MAE is advantageous: While $R^{2}$ offers a relative measure of fit, MAE yields a direct interpretation of prediction error magnitude and is more robust to outliers.

## Supplementary Material

Appendix 01 (PDF)

## Data Availability

Aggregated data at first administrative level and code data have been deposited in Open Science Framework (https://doi.org/10.17605/OSF.IO/5E8WF) (41) and GitHub (https://github.com/OxfordDemSci/dgg_subnational).

## Footnotes

^*^GADM, the Database of Global Administrative Areas, is a publicly available database of country administrative areas. When boundaries are available in the Facebook Marketing API that match the GADM-1 boundaries, we use the default Facebook boundaries. When no boundary is available in the Facebook Marketing that matches the GADM-1 boundaries (30%), we create custom shapefiles to match the GADM-1 boundaries.
