# Supplementary material for: Mapping subnational gender gaps in internet and mobile adoption using social media data
Source: Proc Natl Acad Sci U S A. 2025 Oct 14;122(42):e2416624122. doi: 10.1073/pnas.2416624122 (PMC12557523; doi:10.1073/pnas.2416624122)
Supplement: Supplementary file 1 — Appendix 01 (PDF) [file pnas.2416624122.sapp.pdf]

Supporting Information for

Mapping subnational gender gaps in internet and mobile adoption using social media data

Casey F. Breen, Masoomali Fatehkia, Jiani Yan, Xinyi Zhao, Douglas R. Leasure, Ingmar Weber, and Ridhi Kashyap

Casey F. Breen and Ridhi Kashyap.
E-mail: casey.breen@demography.ox.ac.uk and ridhi.kashyap@nuffield.ox.ac.uk

This PDF file includes:

Supporting text
Figs. S1 to S34
Tables S1 to S8
SI References

Supporting Information Text

1. Feature and ground truth construction

We used several different data sources to construct the features and ground truth measures of digital adoption for our modeling pipeline. A full list of features and ground truth outcomes is shown in Table S1. An overview of the processing steps taken to create different features is shown in Table S2.

1A. Constructing Facebook features. We collected Facebook monthly active user (MAU) counts using the Facebook (Meta) Marketing API. This API, which is publicly accessible, is designed to provide advertisers with tools to help them advertise across Meta platforms. We used the "Ad Account Delivery Estimate" endpoint, which allows users to query MAU counts in a given location with specific attributes (e.g., gender, age, access device type, etc.). The Marketing API only provides current MAU counts and cannot be used to query MAU counts from the past.

To systematically query the Facebook Marketing API, we used an adapted version of the publicly available pysocialwatcher package (1). This software automates repeated queries to the Facebook Marketing API based on a user-provided set of locations and attributes. To construct the Facebook features for our models, we collected MAU counts for each country and admin-1 region by gender (female and male) and access type (iOS, Wi-Fi, and 4G+ mobile network). We collected these data with a daily temporal resolution beginning in 2019 for national-level collections and April 2024 for admin-1-level collections.

We used these MAU counts to construct several different features. First, we calculated overall subnational Facebook penetration features by gender by combining averaged MAU counts with current population estimates from Worldpop. Second, we used these penetration features to calculate the female to male ratio in Facebook penetration. Finally, we calculated the gender-specific fraction of overall users who access Facebook through different means (e.g., fraction of women who accessed Facebook through Wi-Fi). We construct these features both at the national and admin-1-level.

Given the stable, positive trend in national-level MAU counts in most countries, we fit generalized linear models (GLM) with a log link and a linear trend to our national MAU counts from 2019 to 2024. We used these models to impute MAU counts back to 2015. To achieve temporal alignment for subnational features, we rescaled the April 2024 subnational MAU counts using an adjustment factor defined as the ratio of the national MAU count in a given period to the national MAU count in April 2024:

$$MAU_{subnational,year} = MAU_{subnational,2024} \times \underbrace{\frac{MAU_{national,year}}{MAU_{national,2024}}}_{\text{adjustment factor}} . \qquad [1]$$

The result is a longitudinal set of Facebook MAU counts from 2015 to the present at the admin-1 and national level. In our models, we use both the national-level and subnational-level features to capture both overall national adoption and subnational heterogeneity.

The full set of Facebook features used in our model is shown in Table S1. We treat Facebook access method (e.g., WiFi, 4G+, etc.) features as time invariant. We currently have ongoing daily collections at both the subnational and national level. We plan to update the models monthly to incorporate the most current Facebook features to predict contemporary levels and gaps in digital adoption ("nowcast").

1B. DHS processing. We calculated all ground truth estimates of internet and mobile adoption using DHS surveys. We restricted to DHS surveys between 2015 and 2023 that have digital adoption information available for both men and women. For most DHS surveys, the universe of eligible respondents is men and women aged 15–49. We exclude the 2018 Indonesia DHS survey, which only includes married men aged 25–54. In total, we used 33 DHS surveys corresponding to 1,568,617 interviews (Table S3).

We used the GPS location of each DHS clusters to map individuals onto their corresponding admin-1 units. All DHS estimates of digital adoption were calculated using survey weights.

1C. Offline features. The offline features were selected to ensure complete coverage across all LMICs. Additionally, we restricted to features that were measured consistently and harmonized across countries, which constrained the number of offline features included in the model. To construct population estimates by gender and age, we use data from Worldpop's 1km X 1km unstructured grids (2). These data are available from 2015-2020. We calculated a population-density metric, which we standardized using Z-scores.

We used a series of development and gender-equality indicators from the Global Data Lab (3). Specifically, we use indicators capturing human development, gender development, income, and education. We include human development and gender development features at both the national and subnational level to capture overall levels and deviations from national values.

The nightlights data come from the Earth Observation Group (4, 5). We took the mean nightlights value within each subnational unit, and standardized it using Z-scores.

1D. Handling missing values. We selected a parsimonious set of features to ensure broad coverage across LMICs. For the 525 admin-1 units used to train our machine learning model, there were only 8 missing values, all for the gender development index in Guinea. In the full set of 2,075 subnational units, there were no missing values for our key Facebook features. For units with missing offline features (approximately 9% of subnational units), we imputed missing values for a feature using the nearest

available non-missing year. If no data from adjacent years were available, we used the median overall value of the continent the country was in.

We do not make predictions for countries where no available Facebook MAU counts are available: American Samoa, China, Cuba, Fiji, French Southern Territories, Kosovo, Marshall Islands, Mayotte, North Korea, Papua New Guinea, Russia, Réunion, Saint Helena, Ascension, and Tristan da Cunha, Seychelles, Sudan, and Western Sahara.

**Variable Name**	**Type**	**Source**	**Country (N)**	**Subnational (N)**	Temporal Alignment
Used Internet Age 15-49 Women (%)	Ground truth	DHS	33	525	—
Owns Mobile Age 15-49 Wom (%)	Ground truth	DHS	33	525	—
Used Internet Age 15-49 Men (%)	Ground truth	DHS	33	525	—
Owns Mobile Age 15-49 Men (%)	Ground truth	DHS	33	525	—
Used Internet Age 15-49 FM Ratio (%)	Ground truth	DHS	33	525	—
Owns Mobile Age 15-49 FM Ratio (%)	Ground truth	DHS	33	525	—
Nightlight Mean Z-score	Offline	NASA Earth Observations	117	2075	Aligned to survey year
Population Density Z-score	Offline	Worldpop	117	2075	Aligned to survey year
Subnational Gender Development Index (GDI)	Offline	Subnational Dev. Database	117	2075	Aligned to survey year
Subnational Human Development Index (HDI) Men	Offline	Subnational Dev. Database	117	2075	Aligned to survey year
Subnational Human Development Index (HDI) Women	Offline	Subnational Dev. Database	117	2075	Aligned to survey year
Educational Index Females	Offline	Subnational Dev. Database	117	2075	Aligned to survey year
Educational Index Males	Offline	Subnational Dev. Database	117	2075	Aligned to survey year
Income Index Females	Offline	Subnational Dev. Database	117	2075	Aligned to survey year
Income Index Males	Offline	Subnational Dev. Database	117	2075	Aligned to survey year
Human Development Index (HDI) National	Offline	Subnational Dev. Database	117	2075	Aligned to survey year
Gender Development Index (GDI) National	Offline	Subnational Dev. Database	117	2075	Aligned to survey year
Continent	Offline	Constructed	117	2075	Static
Years since 2015	Offline	Constructed	117	2075	Constructed relative to survey yea
FB Penetration 13+ Male 2024	Facebook	FB Marketing API	117	2075	Aligned to survey year
FB Penetration 13+ Female 2024	Facebook	FB Marketing API	117	2075	Aligned to survey year
FB Age 18+ Gender Gap 2024	Facebook	FB Marketing API	117	2075	Aligned to survey year
iOS Age 18+ Female Fraction 2024	Facebook	FB Marketing API	117	2075	Aligned to survey year
iOS Age 18+ Male Fraction 2024	Facebook	FB Marketing API	117	2075	Aligned to survey year
WiFi Age 18+ Female Fraction 2024	Facebook	FB Marketing API	117	2075	Aligned to survey year
WiFi Age 18+ Male Fraction 2024	Facebook	FB Marketing API	117	2075	Aligned to survey year
4G+ Age 18+ Female Fraction 2024	Facebook	FB Marketing API	117	2075	Aligned to survey year
4G+ Age 18+ Male Fraction 2024	Facebook	FB Marketing API	117	2075	Aligned to survey year
FB Penetration 18+ Male 2024 (National)	Facebook	FB Marketing API	117	2075	Aligned to survey year
FB Penetration 18+ Female 2024 (National)	Facebook	FB Marketing API	117	2075	Aligned to survey year
FB Age 18+ Gender Gap (National)	Facebook	FB Marketing API	117	2075	Aligned to survey year

Table S1. List of features and ground truth measures used in the analysis and their source. The temporal alignment column indicates how each feature was aligned in time with the ground truth data.

Data	Data source	Processing Steps
Ground truth estimates of digital adoption	DHS Program	1. Map DHS clusters onto their corresponding admin-1 units using GPS coordinates.<br>2. Calculate gender-specific, population-weighted measures of mobile phone ownership and internet use (past 12 months). Then calculate the digital gender gap indices at the admin-1-level.
Population estimates	WorldPop	1. Obtain Worldpop estimates of population counts by sex and age in each admin-1 unit.<br>2. Aggregate population counts to match the target population (age 15-49).<br>3. Calculate the population density.
Development indicators	Global Data Lab	1. Match the Global Data Lab (GDL) units with the admin-1 units. For matching, we first use exact name matching. If this fails, we perform fuzzy name matching with manual verification. Finally, for units still not matched, we perform geo-matching.<br>2. Calculate Global Data Labs (GDL) variables in each admin-1 unit.
Nightlight data	Earth Observation Group	1. Resample the population raster data.<br>2. Average and weight the nightlight value in each admin-1 unit using the population estimates from WorldPop.
Facebook monthly active user counts	Facebook marketing API	1. Obtain monthly active user (MAU) counts by gender and device type.<br>2. Construct population-weighted measures of Facebook adoption by gender and device type using population estimates from Worldpop.

Table S2. Overview of data processing steps.

Country	Country Code	Start Year	End Year	# Women	# Men
Angola	AO	2015	2016	14379	5684
Armenia	AM	2015	2016	6116	2755
Benin	BJ	2017	2018	15928	7595
Burkina Faso	BF	2021	2021	17659	7720
Burundi	BU	2016	2017	17269	7552
Cambodia	KH	2021	2022	19496	8825
Cameroon	CM	2018	2019	14677	6978
Côte d'Ivoire	CI	2021	2021	14877	7591
Ethiopia	ET	2008	2008	15683	12688
Gabon	GA	2019	2021	11043	6894
Gambia	GM	2019	2020	11865	4636
Guinea	GN	2018	2018	10874	4117
Haiti	HT	2016	2017	15513	9795
India	IA	2019	2021	724115	101839
Kenya	KE	2022	2022	32156	14453
Liberia	LB	2019	2020	8065	4249
Madagascar	MD	2021	2021	18869	9037
Malawi	MW	2015	2016	24562	7478
Mali	ML	2018	2018	10519	4618
Mauritania	MR	2019	2021	15714	5673
Mozambique	MZ	2022	2023	13183	5380
Nepal	NP	2022	2022	14845	4913
Nigeria	NG	2018	2018	41821	13311
Pakistan	PK	2017	2018	15068	3691
Rwanda	RW	2019	2020	14634	6513
Senegal	SN	2019	2019	8649	3365
Sierra Leone	SL	2019	2019	15574	7197
South Africa	ZA	2016	2016	8514	3618
Tanzania	TZ	2022	2022	15254	5763
Timor-Leste	TL	2016	2016	12607	4622
Uganda	UG	2016	2016	18506	5336
Zambia	ZM	2018	2019	13683	12132
Zimbabwe	ZW	2015	2015	9955	8396
Total				1,236,870	331,747

Table S3. The 33 DHS surveys used to construct ground truth estimates of digital adoption.

2. Sustainable development goals

The United Nations sustainable development goals (SDG) are a set of 17 goals and targets to guide international development policy. Originally established in 2015, the SDGs were established to reduce poverty, hunger, AIDS, and discrimination against women and girls (6). Each sustainable development goal has a set of accompanying targets and indicators for tracking development. The estimates produced in this study most directly contribute to monitoring and tracking the following indicators:

- Goal 5: Achieve gender equality and empower all women and girls
  - 5.b.1 Proportion of individuals who own a mobile telephone, by sex
- Goal 4: Ensure inclusive and equitable quality education and promote lifelong learning opportunities for all
  - 4.4.1 Proportion of youth and adults with information and communications technology (ICT) skills, by type of skill
- Goal 17: Strengthen the means of implementation and revitalize the Global Partnership for Sustainable Development
  - 17.8.1 Proportion of individuals using the Internet

A full list of sustainable development goals are available on the UN Website.

3. Modeling

3A. Superlearner weights. The set of machine learning algorithms included in our ensemble Superlearner models and their respective weights are presented in Table S4. Each algorithm's weight represents its contribution to the overall ensemble Superlearner predictions. These weights are estimated using non-negative least squares regression; for more details on practical considerations of implementing a Superlearner, see Phillips et al. 2023 (7).

The algorithms that are most heavily weighted in our final ensemble models are random forest, gradient boosting machines (GBM), and ridge regression. The high weights on random forest and GBM suggest that tree-based models, well suited for capturing non-linear relationships and interactions, often perform best for these prediction tasks.

		Internet			Mobile		
Algorithm	Description	Women	Men	Ratio	Women	Men	Ratio
glm	Generalized Linear Model	0.00	0.00	0.00	0.00	0.00	0.00
lasso	Lasso Regression	0.00	0.00	0.00	0.08	0.00	0.00
ridge	Ridge Regression	0.62	0.45	0.59	0.37	0.06	0.31
elastic_new	Elastic Net with 50% L1 Ratio	0.00	0.00	0.00	0.00	0.00	0.08
poly_spline	Polynomial Spline	0.00	0.00	0.00	0.11	0.13	0.06
random forest	Random Forest with 100 Trees	0.00	0.16	0.25	0.19	0.27	0.00
gbm	Gradient Boosted Machine	0.18	0.40	0.12	0.25	0.54	0.54
xgb	Extreme Gradient Boosting	0.22	0.00	0.03	0.00	0.00	0.00
SuperLearner	Ensemble Model	–	–	–	–	–	–

Table S4. Full set of machine learning algorithms used in the Superlearner and their relative contribution to the final Superlearner model.

3B. Quantifying uncertainty. To estimate uncertainty for each subnational unit, we regressed the absolute residual against a set of all observable variables for each subnational unit with available auxiliary estimates from DHS surveys. This is a standard approach that has been used in past efforts using machine learning for small-area estimation (8). We used a non-negative least squares regression to ensure the resulting estimates are non-negative. We fit separate models by indicator to predict the absolute residual size for all subnational units. Qualitatively, our predicted absolute error is largest in high-adoption settings and smallest in low-adoption settings (Fig. S1). The relative error, defined as the absolute error divided by the predicted value, is largest in low-adoption settings.

A

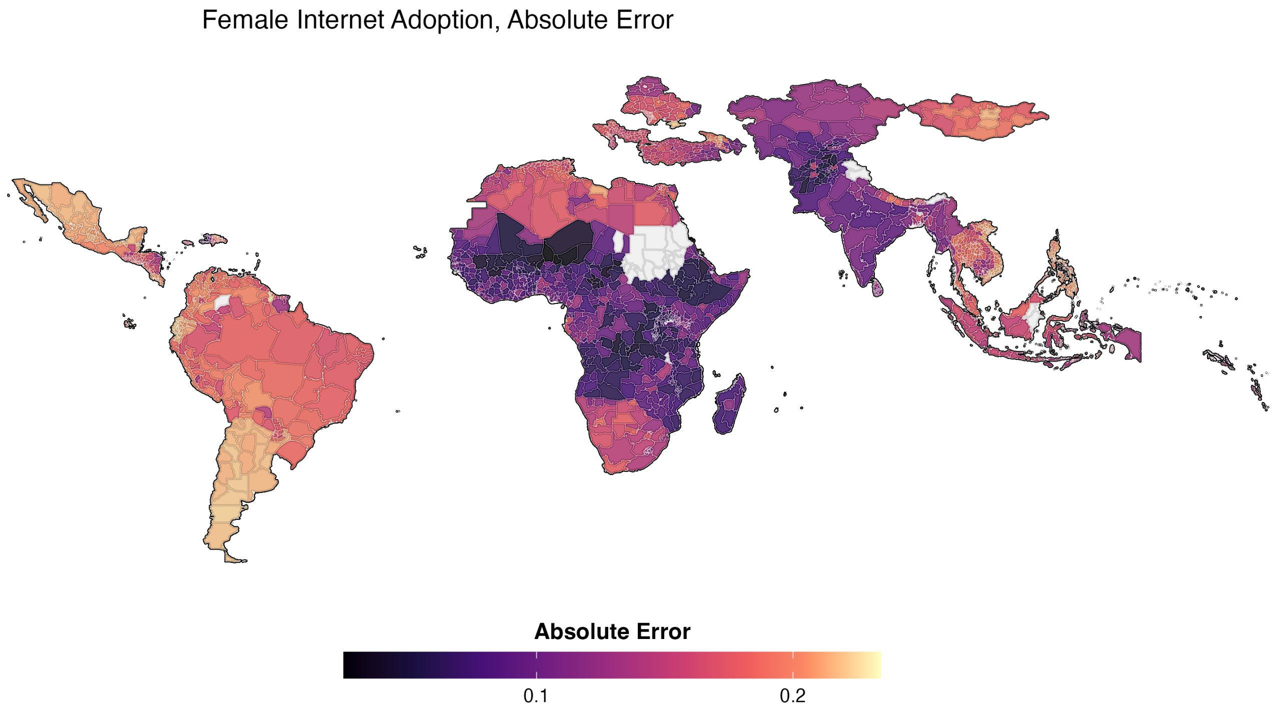

B

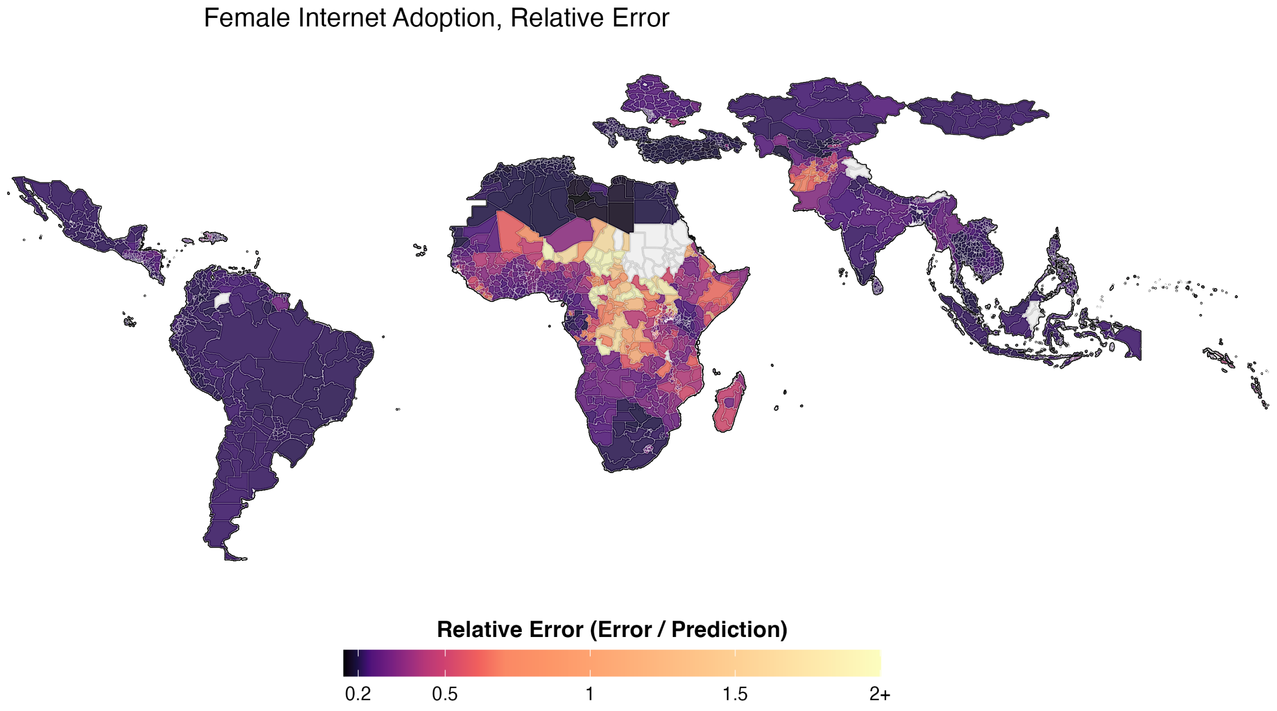

Fig. S1. *(A)* The absolute predicted residual error in each subnational region. A value of 0.1 corresponds to 10 percentage points. *(B)* The relative predicted error, defined as the absolute error divided by the predicted value, in each subnational region.

3C. Bootstrap analysis of uncertainty in DHS ground truth. Our underlying ground truth data, which come from DHS surveys, are subject to sampling variability, especially at the admin-1 level. To understand how this uncertainty affects our analysis, we conduct a bootstrap analysis. Specifically, we repeatedly resample individual survey respondents with replacement within subnational units to create 10,000 synthetic bootstrap samples. We then compare our original estimates of digital adoption to the estimates from our 10,000 bootstrapped samples.

This analysis quantifies the uncertainty in our digital adoption estimates due to sampling variability. As shown in Table S5, there is an upper bound on the R^2 values attainable given the noise in the ground truth data. Notably, maximum R^2 values are consistently lower for the survey-based gender gap index estimates than for overall adoption levels, reflecting higher sampling variation in the former.

Category	Q10	Q50	Q90	Mean
Mobile Women	0.930	0.982	0.996	0.966
Mobile Men	0.786	0.928	0.984	0.886
Mobile Gender Gap Index	0.687	0.908	0.981	0.843
Internet Women	0.960	0.988	0.997	0.981
Internet Men	0.919	0.974	0.993	0.963
Internet Gender Gap Index	0.407	0.843	0.959	0.709

Table S5. Percent of total variation explained (R^2) from 10,000 bootstrap resamples

3D. Decomposing within and between country variance. To assess the relative contribution of within-country and between country variation to overall inequality, we used mean log deviation. Mean log deviation has appealing decomposability properties, and has been used elsewhere to quantify the additional inequality revealed at the subnational level (9). The mean log deviation (MLD) for all subnational units is defined as:

$$\text{MLD} = \frac{1}{N}\sum_{i=1}^{N} \log\left(\frac{\mu}{r_i}\right), \quad [2]$$

where N is the number of subnational units, μ is the overall mean rate (e.g., mean internet penetration rate), and r_i is the rate for a given subnational unit i. The MLD can be decomposed into within-country and between-country components:

$$\text{MLD} = \text{MLD}_W + \text{MLD}_B. \quad [3]$$

The overall mean rate μ can be expressed as:

$$\mu = \sum_{k=1}^{K} \frac{N_k}{N}\mu_k, \quad [4]$$

where K is the number of countries, N_k is the number of subnational units in country k, and μ_k is the mean rate for country k. The within-country MLD for each country k is:

$$\text{MLD}_k = \frac{1}{N_k}\sum_{i\in k} \log\left(\frac{\mu_k}{r_i}\right). \quad [5]$$

The overall within-country MLD is a weighted sum of the within-country MLDs:

$$\text{MLD}_W = \sum_{k=1}^{K} \frac{N_k}{N}\text{MLD}_k. \quad [6]$$

The between-country MLD is calculated based on the mean rates of the countries:

$$\text{MLD}_B = \sum_{k=1}^{K} \frac{N_k}{N} \log\left(\frac{\mu}{\mu_k}\right). \quad [7]$$

The resulting estimates of MLD_B and MLD_k indicate how much of the total inequality in the rate (e.g., internet penetration rate) is due to within-country disparities versus between-country disparities.

3E. Validation against external estimates. To assess external validity, we benchmark our estimates against two independent survey-based sources of subnational digital adoption estimates: the Living Standards Measurement Study (LSMS) and the Multiple Indicator Cluster Surveys (MICS). None of these surveys were used in model training, allowing us to assess model performance against external ground truth. Comparisons between our model-based estimates and these external surveys are imperfect due to inconsistent question wording, differences in reference periods (e.g., internet use in the past 12 vs. past 3 months), and sampling variation due to small sample sizes after subnational disaggregation (10). We benchmark against the MICS and LSMS surveys separately, addressing harmonization and comparability challenges specific to each.

The Living Standards Measurement Study (LSMS) surveys are high-quality, nationally representative surveys conducted by the World Bank (11). Each survey captures detailed information on income, expenditures, demographics, health, and other socioeconomic indicators, such as digital adoption. The majority, but not all, surveys collect individual-level measures of mobile phone ownership and internet use. Each LSMS survey provides geographic identifiers that enable analysis at the admin-1 level. In total, we benchmark against 21 LSMS surveys at the admin-1 level, as listed in Table S6.

Country	Country Code	Survey Year	# Women (Internet)	# Men (Internet)	# Women (Mobile)	# Men (Mobile)
Burkina Faso	BFA	2018	10665	8751	10665	8751
Burkina Faso	BFA	2021	11307	9297	11307	9297
Côte d'Ivoire	CIV	2021	14723	13108	14723	13108
Ethiopia	ETH	2021	0	0	2578	2908
Guinea-Bissau	GNB	2018	10942	9615	10942	9615
Guinea-Bissau	GNB	2021	10816	9841	10816	9841
Cambodia	KHM	2019	0	0	1241	1104
Mali	MLI	2018	9566	7996	9566	7996
Mali	MLI	2021	9866	8399	9866	8399
Malawi	MWI	2016	0	0	12223	10868
Malawi	MWI	2019	0	0	11528	10404
Malawi	MWI	2020	0	0	2211	2085
Niger	NER	2018	7530	5978	7530	5978
Niger	NER	2021	8565	6547	8565	6547
Nigeria	NGA	2015	5696	5147	5702	5151
Nigeria	NGA	2018	6366	5797	5201	5119
Nigeria	NGA	2023	6548	6087	4884	4824
Senegal	SEN	2018	16256	12718	16256	12718
Senegal	SEN	2021	16340	12287	16340	12287
Togo	TGO	2018	6370	5489	6370	5489
Togo	TGO	2021	6667	5628	6667	5628
Total			158223	132685	185181	158117

Table S6. The 21 LSMS surveys used for external validation.

To measure mobile phone ownership, LSMS generally uses the same definition as our study (and DHS surveys): whether an individual personally owns a mobile phone. However, across LSMS surveys, definitions can vary subtly. For instance, in the Nigerian LSMS surveys, questions about mobile phone ownership are framed in terms of access to a mobile phone rather than explicit individual-level ownership. We exclude these surveys from our mobile phone benchmarking exercises. For internet adoption, the LSMS uses a slightly different measure: whether an individual has internet access. This contrasts with the definition used in our study (and DHS surveys) of whether an individual has used the internet in the past 12 months. Having access to the internet and having used the internet in the past 12 months are highly related yet distinct.

To align with the age universe of our study and the DHS, we restrict LSMS respondents to those aged 15–49. To minimize noise due to sampling variation, we only present comparisons for subnational units with at least 150 relevant observations (e.g., 150 or more women when estimating mobile phone adoption among women).

For background, we first compare DHS and LSMS estimates at the subnational level in countries where both surveys were conducted. As shown in Fig. S2, we see general agreement between the estimates of digital adoption. However, the LSMS surveys systematically underestimate the internet gender gap index relative to DHS surveys. This discrepancy likely stems from the surveys' slightly different definitions of internet use: having access to the internet does not equate directly into internet use.

Fig. S3 benchmarks our subnational estimates with those from the LSMS surveys. Our model-based estimates have strong overall agreement with the LSMS estimates. However, the LSMS surveys again systematically underestimate the internet gender gap relative to both our predictions. This mirrors the disagreement between the LSMS and DHS estimates.

For background, we first compare DHS and LSMS estimates at the subnational level in countries where both surveys were conducted. As shown in Fig. S2, we see general agreement between estimates of digital adoption. However, the LSMS surveys systematically underestimate the internet gender gap index relative to DHS surveys. This discrepancy likely stems from the surveys' slightly different definitions of internet use: having access to the internet does not equate directly into internet use. Figure S3 benchmarks our subnational estimates with those from the LSMS surveys. Our model-based estimates have strong overall agreement with the LSMS estimates. However, the LSMS surveys again systematically underestimate the internet gender gap relative to both our predictions. This mirrors the disagreement between the LSMS and DHS estimates.

Fig. S3 benchmarks our subnational estimates with those from the LSMS surveys. Our model-based estimates have strong overall agreement with the LSMS estimates. However, the LSMS surveys systematically underestimate the internet gender gap relative to both our predictions and ground truth from the DHS. This mirrors the disagreement between the LSMS and DHS estimates.

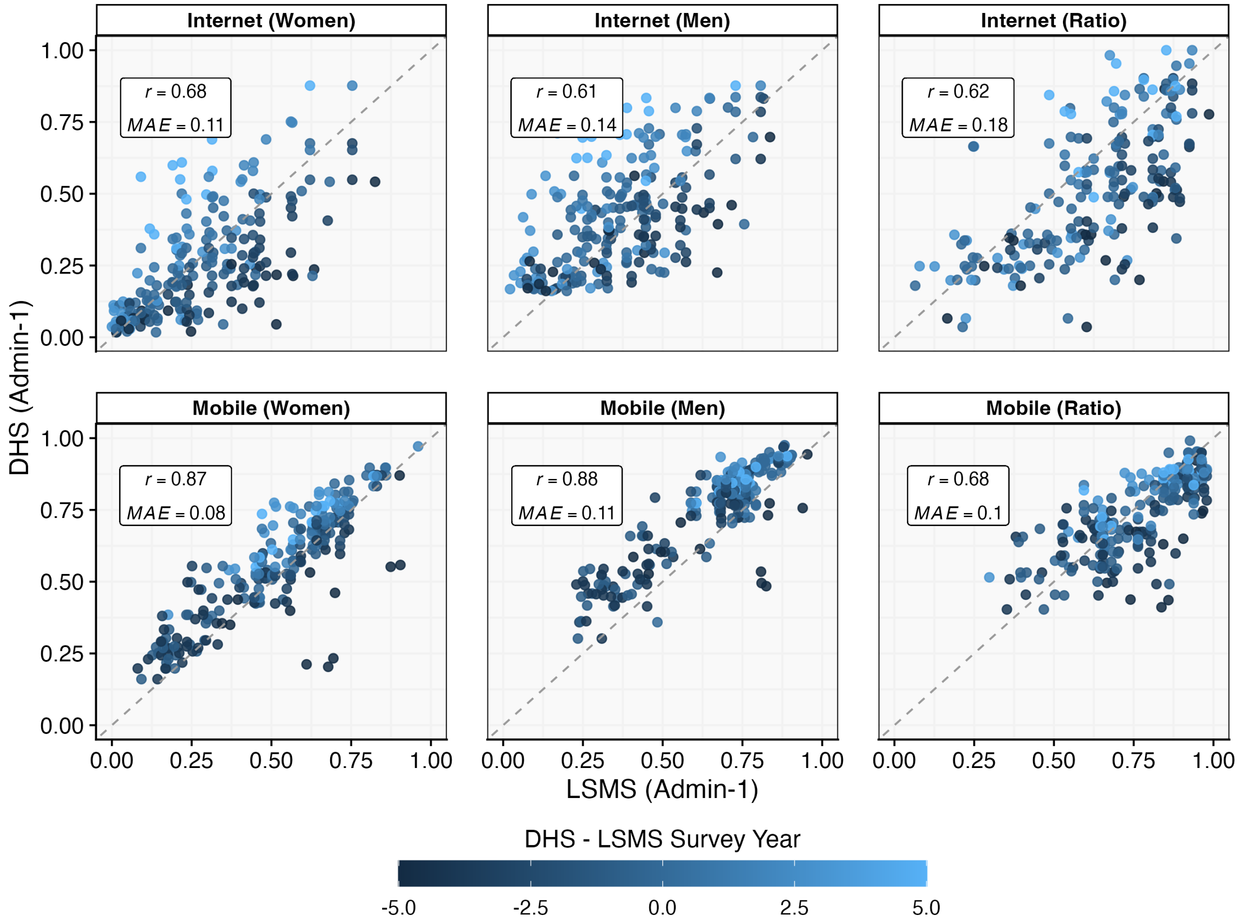

Fig. S2. Comparison of DHS vs. LSMS estimates of adoption levels and gaps. The r denotes the Pearson correlation coefficient and MAE denotes mean absolute error.

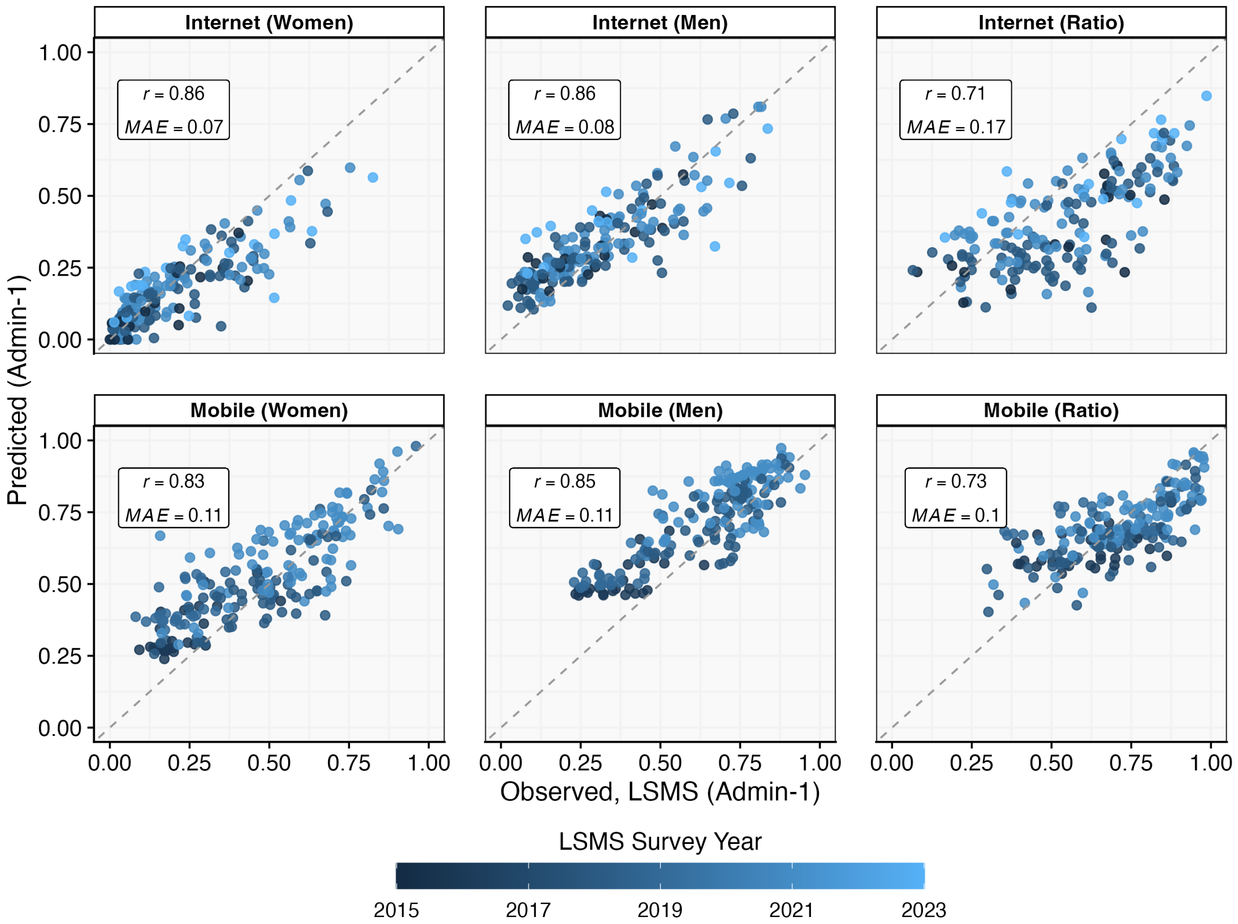

Fig. S3. Predicted values vs. observed out-of-sample values from LSMS surveys. All comparisons are at the admin-1 level.The r denotes the Pearson correlation coefficient and MAE denotes mean absolute error.

Benchmark against MICS surveys The Multiple Indicator Cluster Surveys (MICS) surveys are global household surveys developed by UNICEF to collect internationally comparable data on health and well-being (12, 13). MICS Round 6 surveys generally collect individual-level information on internet use and mobile phone adoption. A subset of these surveys includes admin-1 geographic identifiers, enabling subnational comparisons. We restrict the MICS surveys to the same age range as used in our study and in the DHS, men and women between age 15-49. The full set of 24 MICS surveys is shown in Table S7.

Digital adoption is measured similarly across MICS and DHS (and our study), with nearly identical survey questions. However, MICS and DHS differ slightly in how they ask about internet use: MICS uses a 3-month recall window for internet use, while DHS (and this study) uses a 12-month recall window. To reconcile this, we constructed an adjustment factor that converts the 3-month estimates to the 12-month estimates. Using a subset of surveys that ask about internet usage in both the past 3 months and the past 12 months, we estimate a simple linear correction at the subnational level. Specifically, we adjust the 3-month estimates by 1.0153 to obtain the corresponding 12-month estimate.

Country	Country Code	Survey Year	# Women (Internet)	# Men (Internet)	# Women (Mobile)	# Men (Mobile)
Afghanistan	AFG	2022	44341	0	44341	0
Benin	BEN	2021	18436	7916	18436	7916
Bangladesh	BGD	2019	64377	0	64377	0
Congo - Kinshasa	COD	2017	21756	6113	21756	6113
Comoros	COM	2022	6945	2850	6945	2850
Cuba	CUB	2019	8849	3700	8843	3699
Guinea-Bissau	GNB	2018	10946	2805	10945	2805
Guyana	GUY	2019	5887	2214	5887	2212
Iraq	IRQ	2018	30660	0	30660	0
Jamaica	JAM	2022	4890	0	4889	0
Kyrgyzstan	KGZ	2018	5742	0	5742	0
Kyrgyzstan	KGZ	2023	5629	0	5629	0
Kiribati	KIR	2020	4150	2083	4150	2083
Nigeria	NGA	2021	38810	17347	38810	17347
Nauru	NRU	2023	651	328	651	328
Sierra Leone	SLE	2017	17873	7415	17873	7415
Suriname	SUR	2018	7000	2828	6998	2827
Eswatini	SWZ	2021	2007	1658	2007	1658
Turks & Caicos Islands	TCA	2019	824	364	824	364
Chad	TCD	2019	22564	6931	22567	6931
Turkmenistan	TKM	2019	7558	0	7558	0
Tonga	TON	2019	2903	1232	2903	1232
Vanuatu	VUT	2023	3412	1389	3412	1389
Zimbabwe	ZWE	2019	10130	4179	10130	4179
Total			346340	71352	346333	71348

Table S7. The 24 MICS surveys used for external validation.

In Fig. S4, we benchmark our admin-1 estimates against MICS estimates. Across six digital adoption indicators, we find strong agreement between our model-based estimates and the MICS estimates. In Fig. S5, we show subnational results for three Latin American countries—Guyana, Jamaica, and Suriname. Even in these higher-adoption settings in South and Central America, our estimates closely match the MICS ground truth.

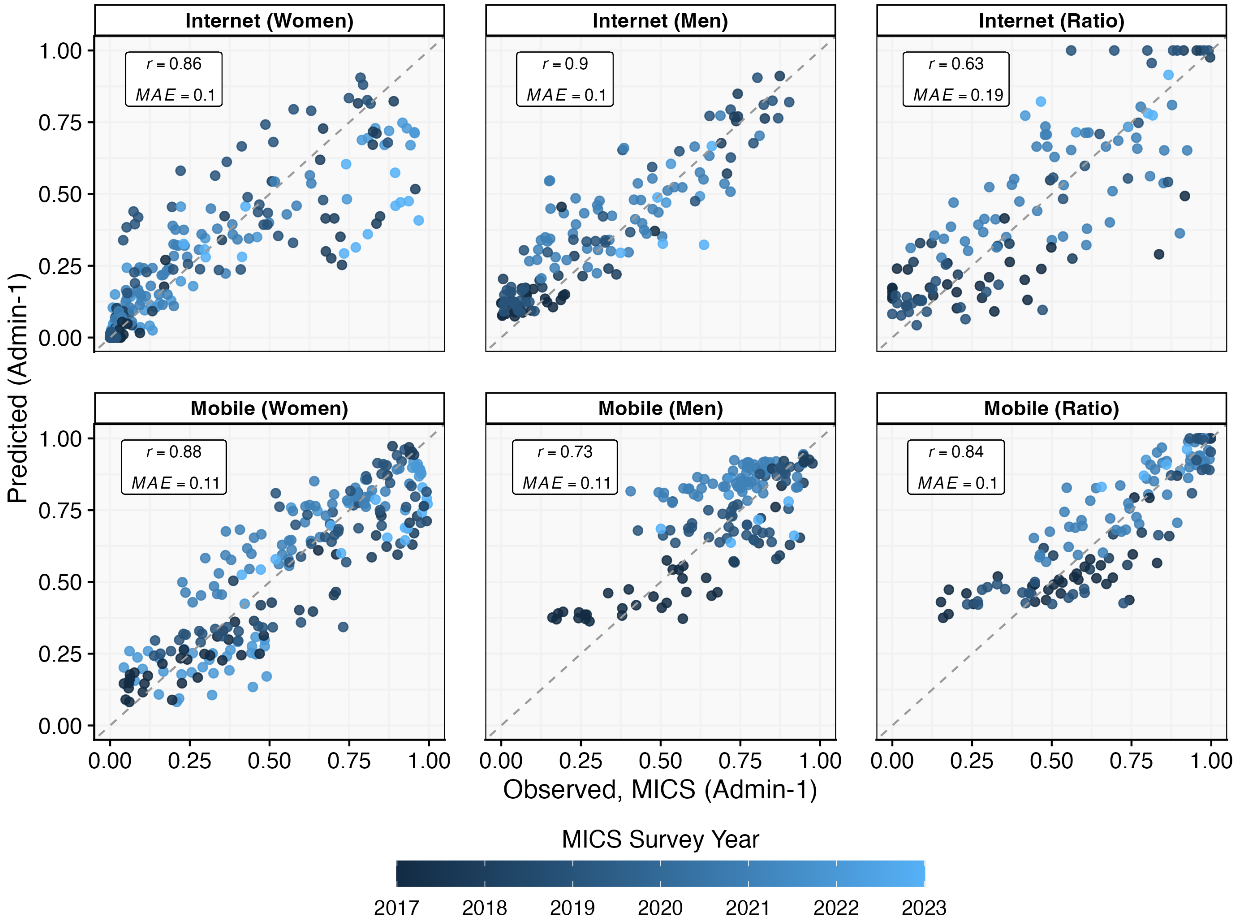

Fig. S4. Comparison of admin-1-level estimates with independent ground truth data from Multiple Indicator Cluster Surveys (MICS).

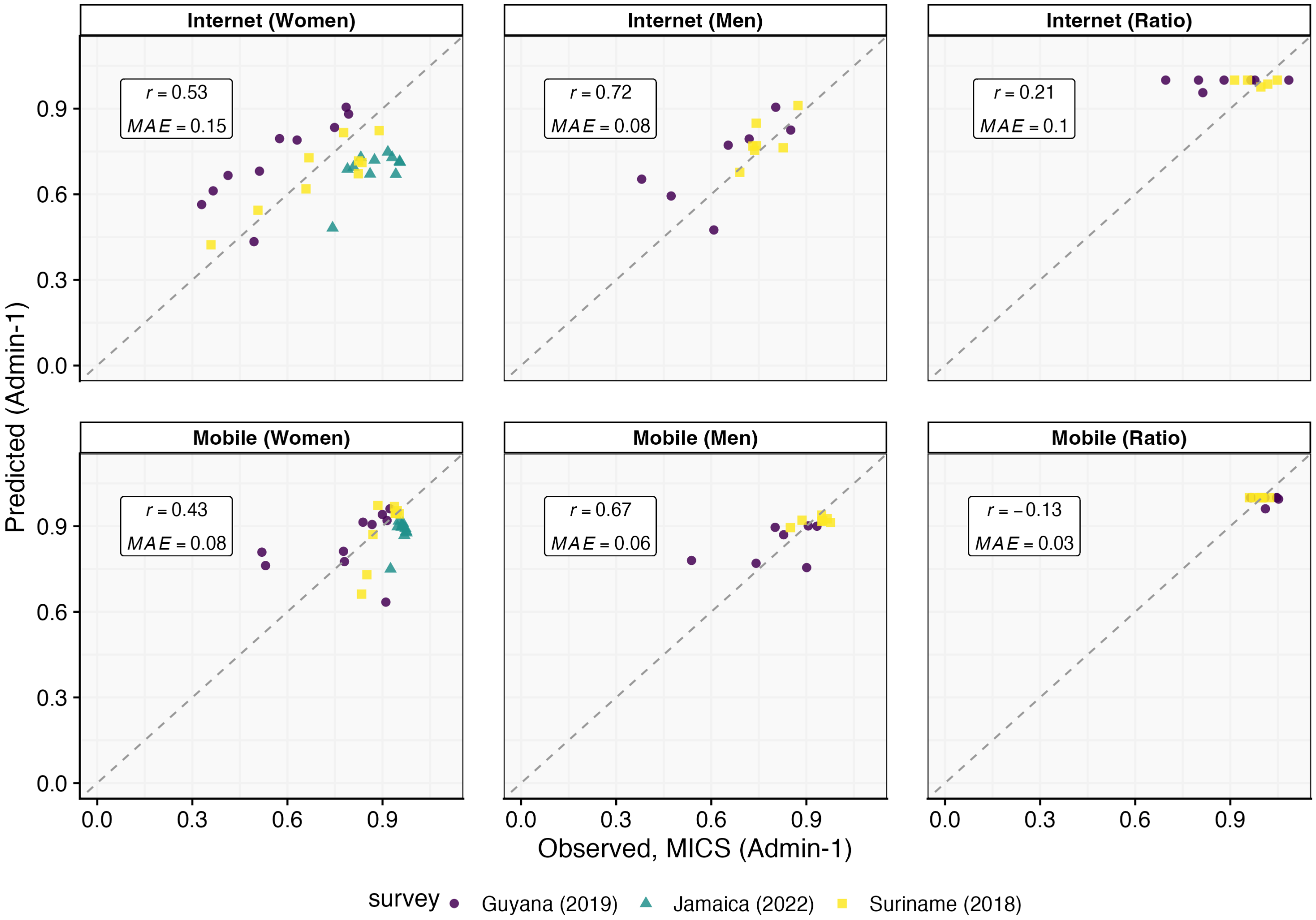

Fig. S5. Comparison of subnational-level estimates with independent ground truth data from Multiple Indicator Cluster Surveys (MICS) for three high-adoption countries in Central and South America. The correlations are modest because of the limited variation in ground truth values, especially for the mobile and internet gender gap indices, where most subnational estimates cluster near 1.0.

Finally, we benchmark at the national-level MICS indicators in Fig. S6. Our aggregated national-level predictions are highly correlated at the national-level with low mean absolute errors (MAE), indicating no systematic national-level over- or underestimation

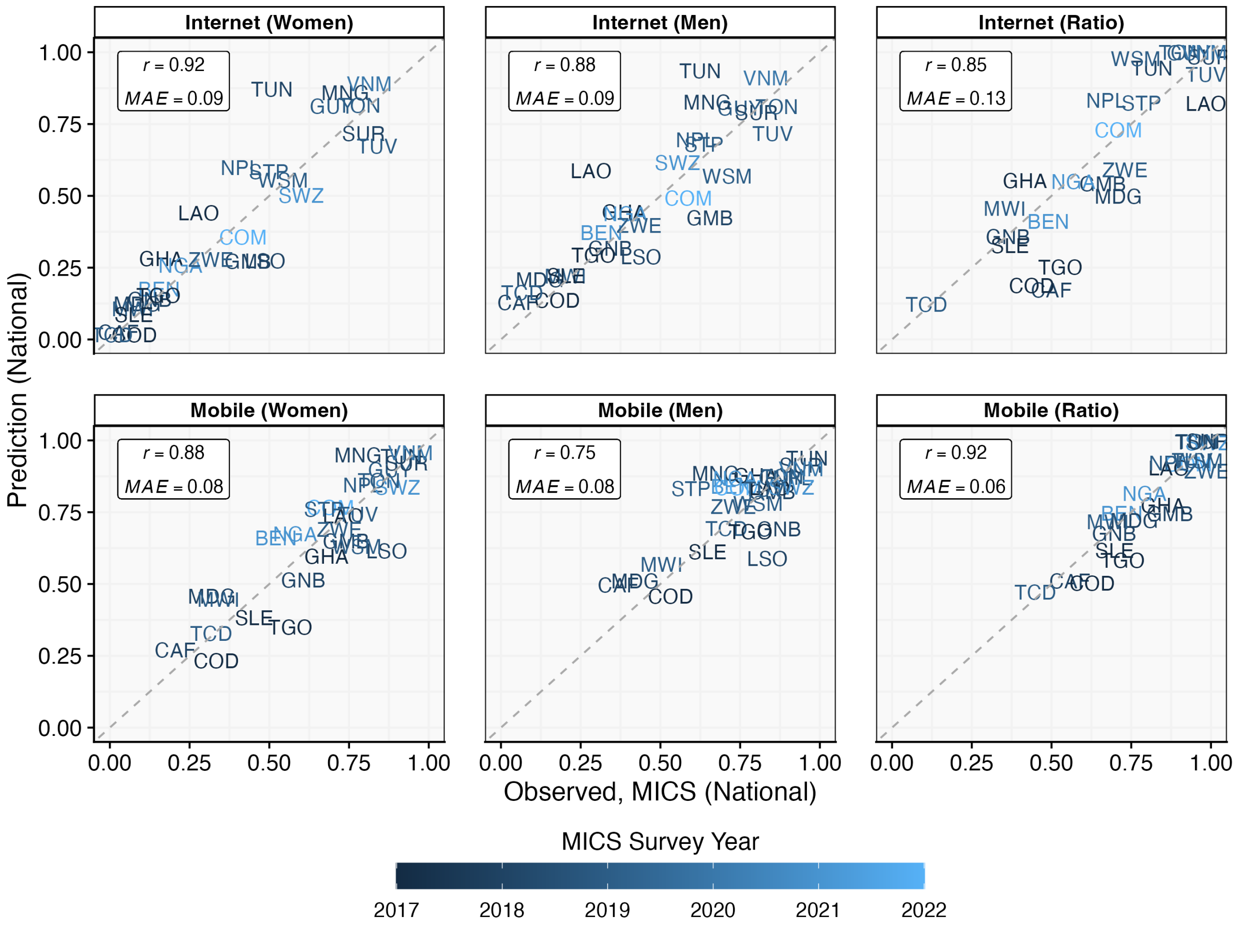

Fig. S6. Comparison of national-level estimates with independent ground truth data from Multiple Indicator Cluster Surveys (MICS) for recent MICS surveys (2019 onwards). MICS surveys are nationally representative, offering valuable independent estimates for comparison. National-level predictions are population-weighted averages of subnational predictions.

3F. Validation of trends. We conducted several analyses to validate our models' ability to generalize across time and space and to capture trends in digital adoption. First, to assess temporal generalization, we examined trends in mean error and mean absolute error over time for each model. Our leave-one-country-out cross-validation (LOCO-CV) exercise shows no change in performance metrics over time (Fig. S7), indicating stable performance and no temporal bias. We replicate this analysis using external LSMS surveys in Fig. S8, again finding no evidence of temporal bias. These results support the ability of our models to generalize over time.

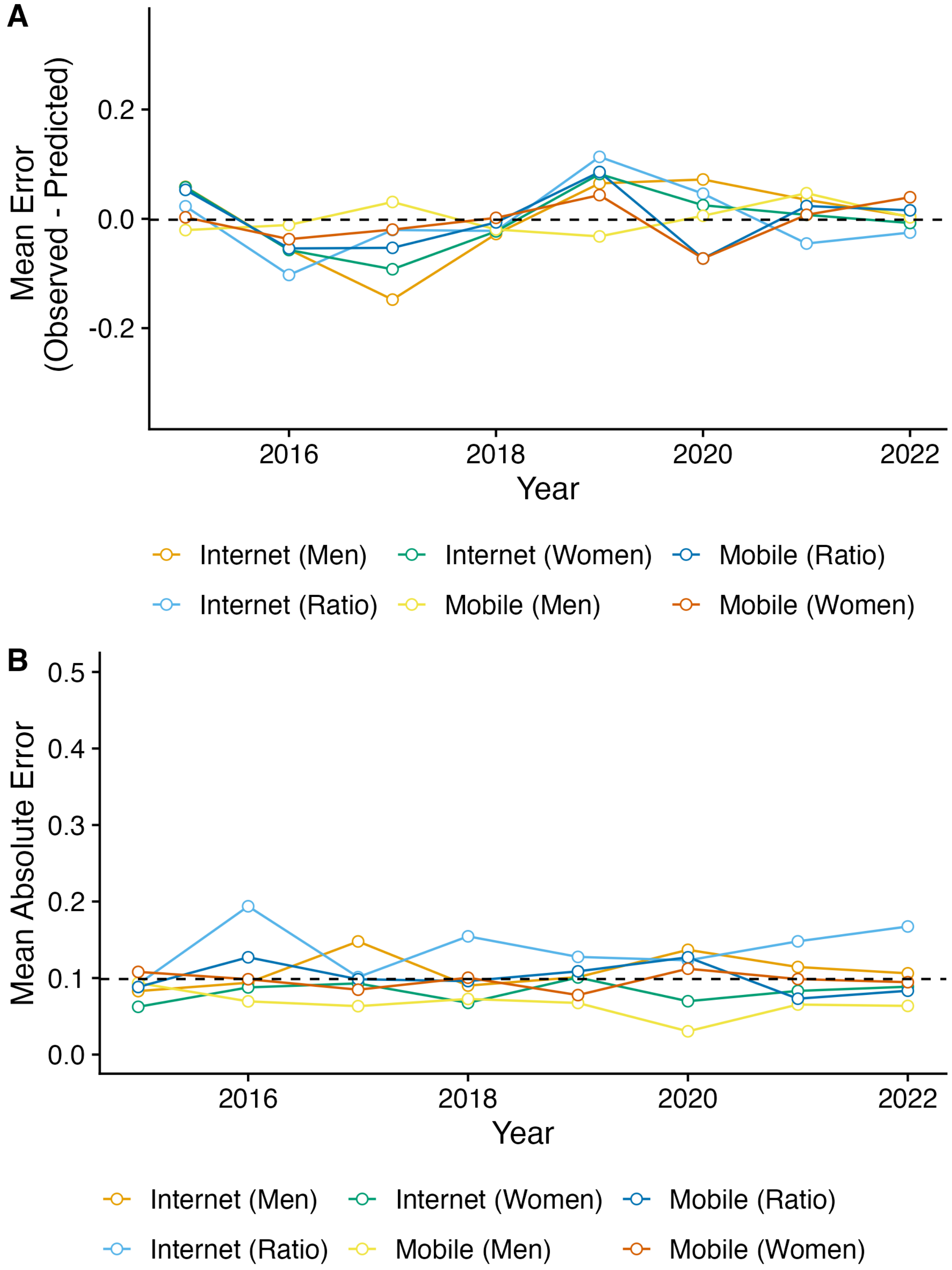

Fig. S7. (*A*) Mean error, defined as the mean of the observed minus predicted values, by year and indicator, assessed using LOCO-CV. (*B*) Mean absolute error, defined as the mean of the absolute difference between observed and predicted values, by year and indicator, also assessed using LOCO-CV. Dashed black lines represent the average error (Panel *A*) and mean absolute error (Panel *B*) across all indicators and years.

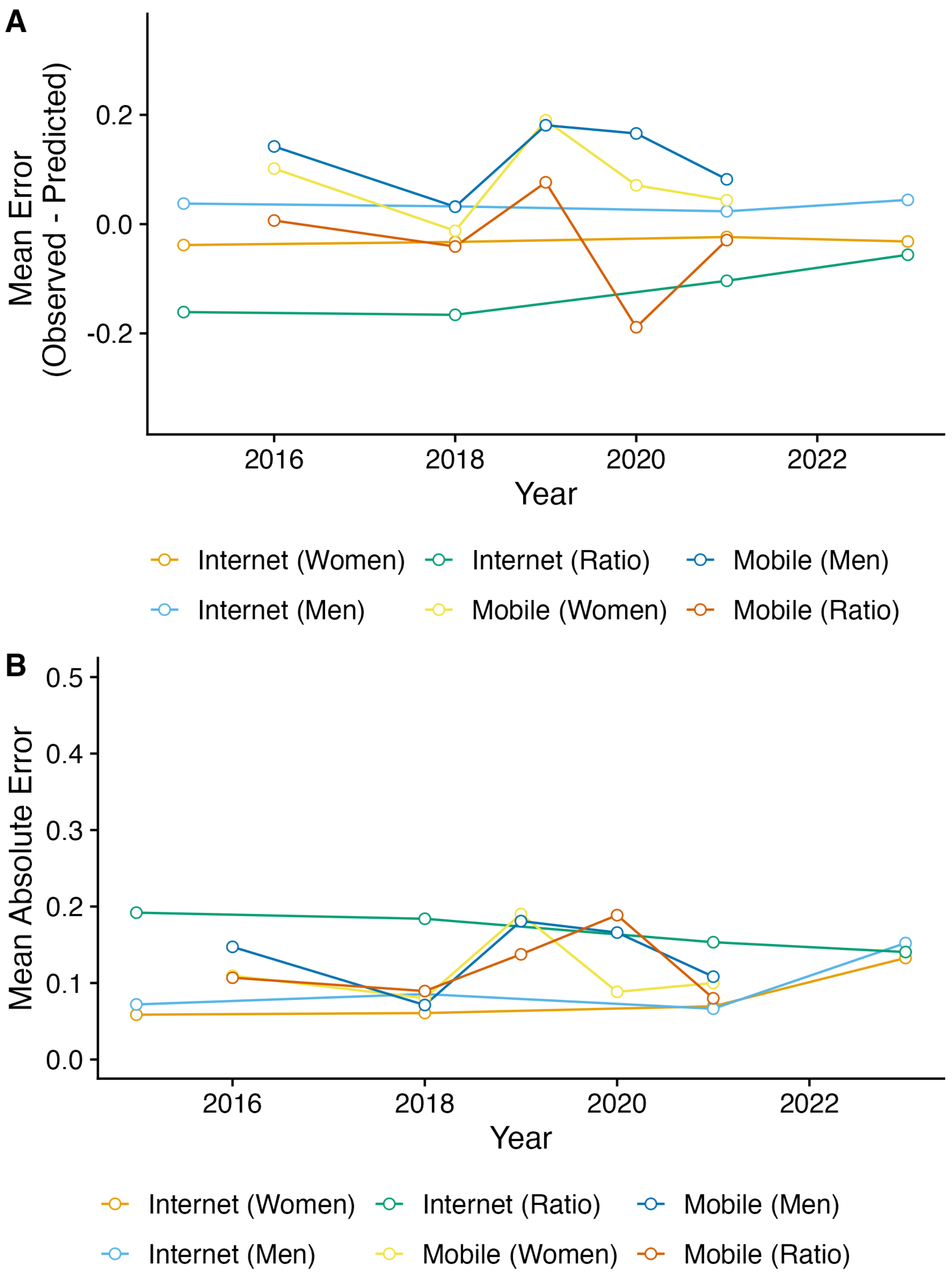

Fig. S8. Mean error, defined as the mean of the observed minus predicted values, by year and indicator, assessed using LSMS survey ground truth. *(B)* Mean absolute error, defined as the mean of the absolute difference between observed and predicted values, by year and indicator, also assessed using LSMS survey ground truth.

As an additional validation exercise, we compare our model-based estimates of change over time to changes observed across repeated survey waves from DHS, MICS, and LSMS. Table S8 lists all countries with multiple survey waves included in this exercise. As discussed in Section 3E, differences in survey design and question wording, as well as small subnational sample sizes, limit the reliability of survey-based estimates of change over time. Due to these differences, we cannot treat survey-based estimates of change over time as ground truth in this setting in a straightforward way. Given these design differences, sampling variability, and the relatively short time windows, we expect only modest agreement between model-predicted and survey-based estimates.

To illustrate, we compare our model-based estimates to survey-based estimates for Nigeria, a country where DHS, MICS,

and LSMS surveys were conducted after 2015. Fig. S9 plots survey-based estimates against our predicted estimates of internet adoption for women. Our estimated time trends largely align with the survey-based measures. In many cases, the model predictions align more closely with the survey-based estimates than the survey-based estimates do with each other. This highlights the noise inherent in survey-based estimates of change in digital adoption over time.

Fig. S10 compares the change between the first and last available survey estimates to the corresponding model-predicted change at the admin-1 level. Across indicators, the correlation between predicted and survey-based change ranges from 0.21 to 0.38. This moderate correlation is expected given the aforementioned noisiness of multi-year change from survey data. Differences in survey design, sampling variability, and short observation windows constrain the maximum attainable alignment between predicted and survey-based estimates of change over time at the subnational level. In some cases, the survey-based estimates indicate negative change, which likely reflects sampling variability rather than true declines in adoption. These results nonetheless suggest that the model is not merely reproducing static spatial patterns, but is instead learning meaningful temporal dynamics. In other words, the model is sensitive not only to where digital adoption is higher or lower, but also to how adoption is changing over time within specific subnational units.

When multiple surveys were available in a given year, we prioritized DHS surveys. We excluded the 2015 LSMS survey in Nigeria due to its small sample size, and instead estimate change over time using the 2018 Nigeria DHS survey and the 2023 Nigeria LSMS survey.

Country	Country Code	Survey Year	Source
Benin	BEN	2017	DHS
Benin	BEN	2021	MICS
Burkina Faso	BFA	2018	LSMS
Burkina Faso	BFA	2021	DHS
Burkina Faso	BFA	2021	LSMS
Côte d'Ivoire	CIV	2021	DHS
Côte d'Ivoire	CIV	2021	LSMS
Ethiopia	ETH	2016	DHS
Ethiopia	ETH	2021	LSMS
Guinea-Bissau	GNB	2018	LSMS
Guinea-Bissau	GNB	2018	MICS
Guinea-Bissau	GNB	2021	LSMS
Kyrgyzstan	KGZ	2018	MICS
Kyrgyzstan	KGZ	2023	MICS
Cambodia	KHM	2019	LSMS
Cambodia	KHM	2021	DHS
Mali	MLI	2018	DHS
Mali	MLI	2018	LSMS
Mali	MLI	2021	LSMS
Malawi	MWI	2015	DHS
Malawi	MWI	2016	LSMS
Malawi	MWI	2019	LSMS
Malawi	MWI	2020	LSMS
Niger	NER	2018	LSMS
Niger	NER	2021	LSMS
Nigeria	NGA	2015	LSMS
Nigeria	NGA	2018	DHS
Nigeria	NGA	2018	LSMS
Nigeria	NGA	2021	MICS
Nigeria	NGA	2023	LSMS
Nepal	NPL	2016	DHS
Nepal	NPL	2022	DHS
Philippines	PHL	2017	DHS
Philippines	PHL	2022	DHS
Senegal	SEN	2017	DHS
Senegal	SEN	2018	DHS
Senegal	SEN	2018	LSMS
Senegal	SEN	2019	DHS
Senegal	SEN	2021	LSMS
Senegal	SEN	2023	DHS
Sierra Leone	SLE	2017	MICS
Sierra Leone	SLE	2019	DHS
Togo	TGO	2018	LSMS
Togo	TGO	2021	LSMS
Tanzania	TZA	2015	DHS
Tanzania	TZA	2022	DHS
Zimbabwe	ZWE	2015	DHS
Zimbabwe	ZWE	2019	MICS

Table S8. Countries with repeated DHS, MICS, or LSMS surveys with information on individual-level digital adoption for women and men and available admin-1 geographic identifiers.

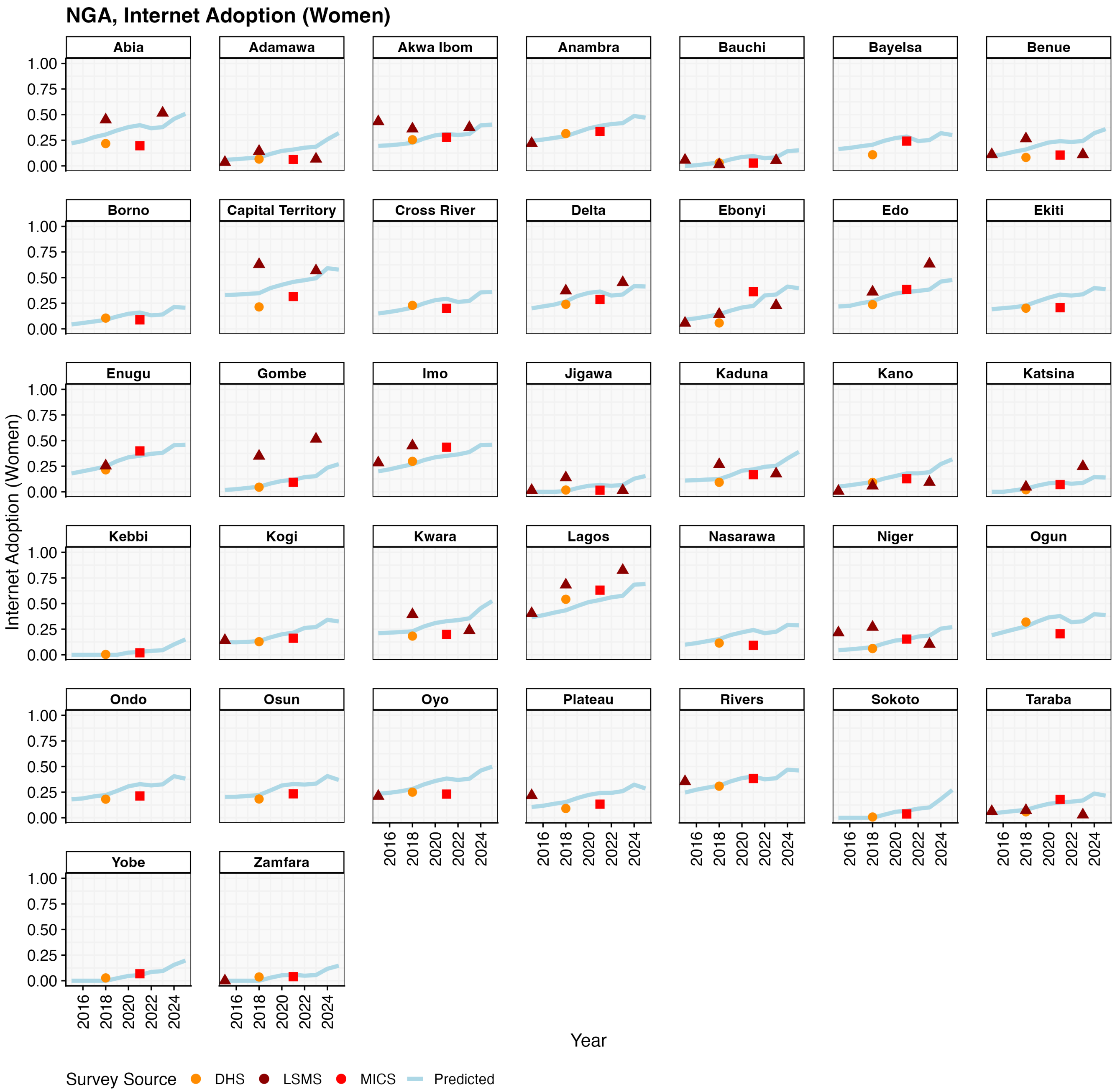

Fig. S9. For admin-1 units in Nigeria, the predicted internet adoption for women (blue line) vs. survey ground truth from DHS (2018), MICS (2021) and LSMS (2015, 2018, 2023). Survey-based estimates are only shown if the admin-1 unit has at least 150 women respondents for a given survey and year.

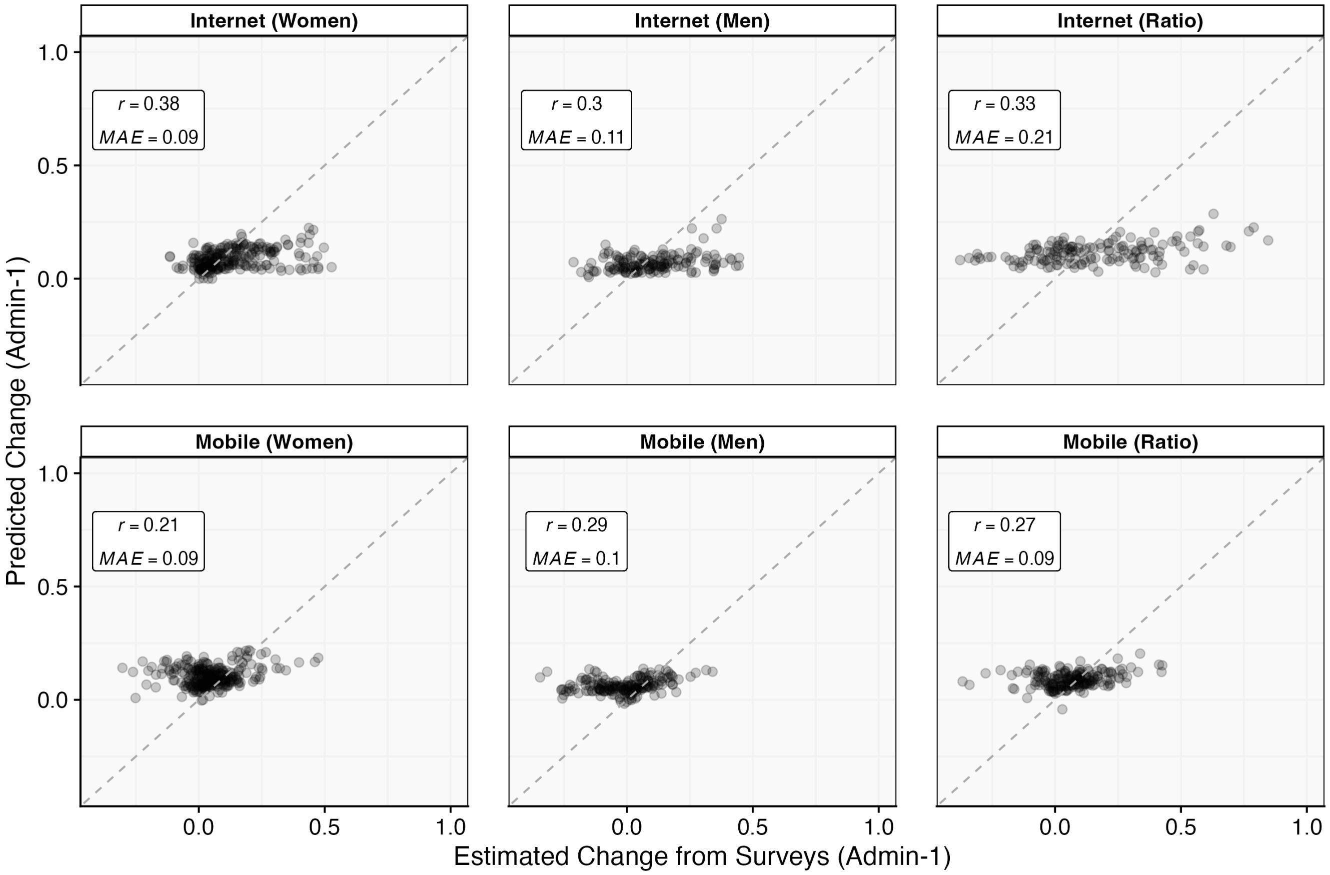

Fig. S10. Survey-based estimates of change over time vs. our model predicted change over time in digital adoption across all countries with multiple survey waves (DHS, MICS, LSMS). Each point represents a subnational (admin-1) unit. Analysis is restricted to units with at least 150 relevant observations in both surveys.
Note: We do not consider survey-based estimates of change over time to be reliable ground truth for benchmarking. Differences in survey design, sampling variability, and short observation windows constrain the maximum attainable alignment. We include these comparisons to assess alignment despite known limitations, but caution against overinterpretation: subnational estimates of multi-year change are subject to substantial noise. Low to moderate correlation is expected and does not reflect the models' inability to capture trends.

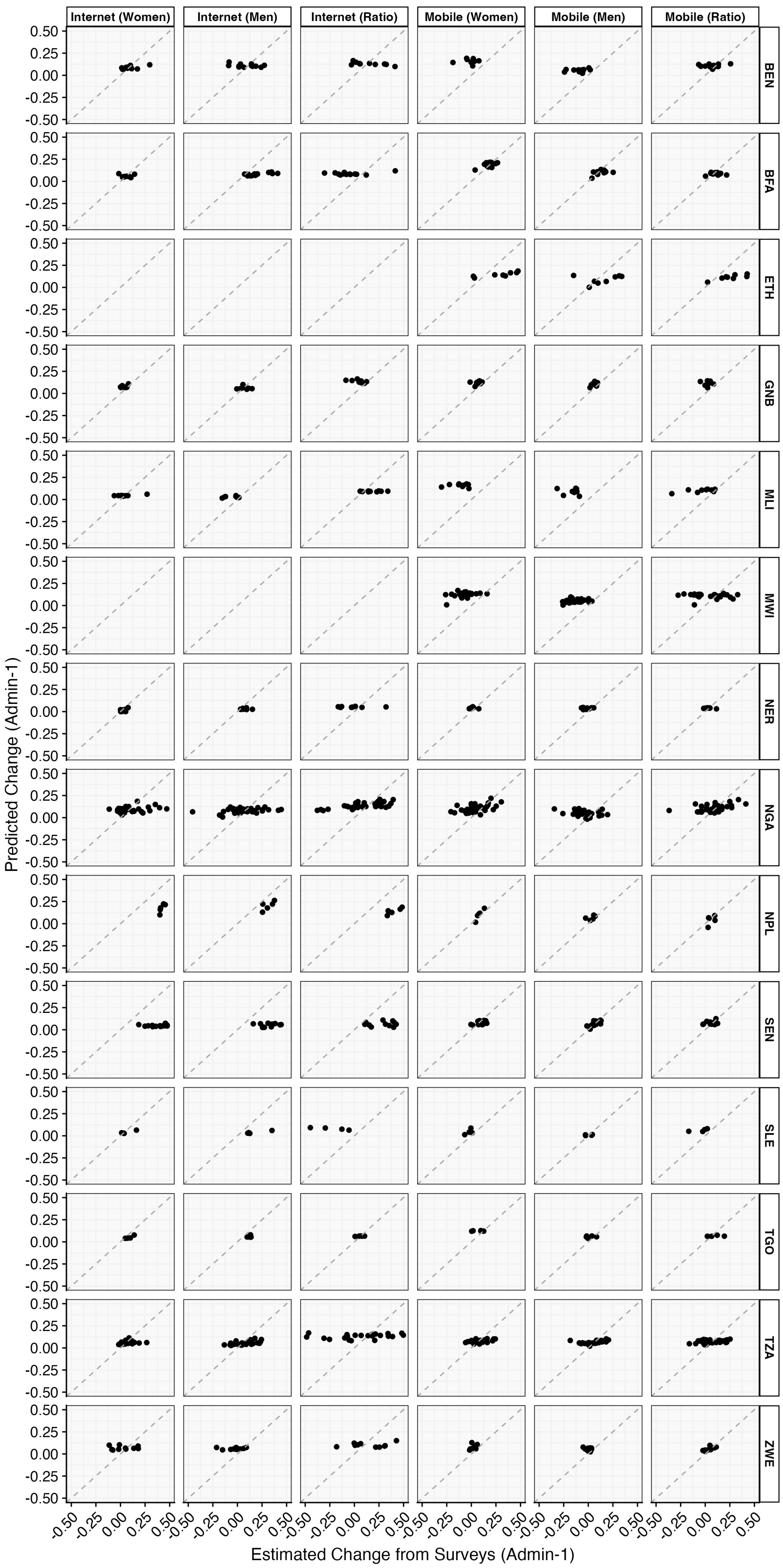

Fig. S11. Survey-based estimates of change over time vs. predicted change over time in digital adoption for countries with multiple survey waves (DHS, MICS, LSMS). Analysis is restricted to admin-1 units with over 150 relevant observations in both surveys.

Note: We do not consider survey-based estimates of change over time to be reliable ground truth for benchmarking. Differences in survey design, sampling variability, and short observation windows constrain the maximum attainable alignment. We include these comparisons to assess alignment despite known limitations, but caution against overinterpretation: subnational estimates of multi-year change are subject to substantial noise. Low to moderate correlation is expected and does not reflect the models' inability to capture trends.

We further benchmark our estimates against those from the 2015 Tanzania DHS survey. Tanzania is a helpful case study for assessing trends as it has two different DHS surveys that measured digital adoption, the first in 2015 and the second in 2022. Our model only uses the most recently available survey year of 2022, allowing for an independent validation against the 2015 estimates. As shown in Fig. S12, our model is able to produce accurate estimates for Tanzania in 2015.

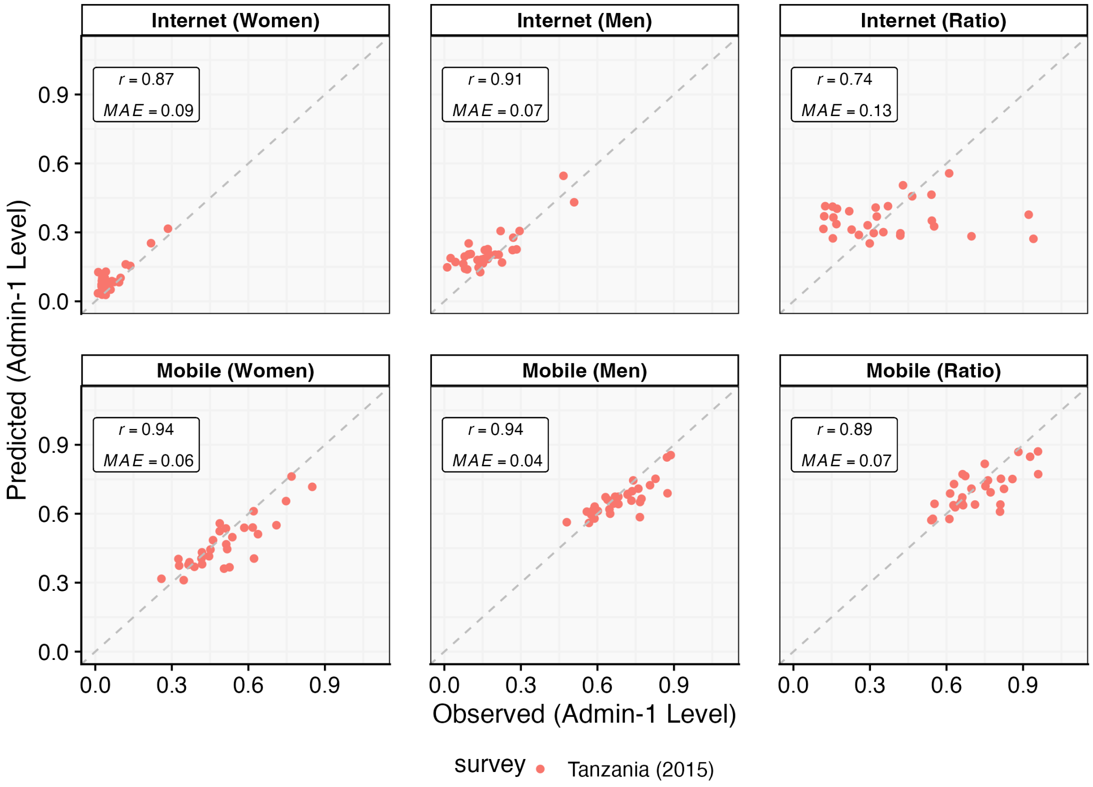

Fig. S12. Predicted vs. observed values for Tanzania in 2015. The 2015 Tanzania survey was not used to train the model, but we see high overall agreement between the estimates of adoption in Tanzania.

3G. Validation of estimates of uncertainty. To validate our uncertainty, we compare our estimated predicted errors against our true observed errors in the LSMS surveys. Fig. S13 shows that across the seven LSMS surveys considered, our observed and predicted errors are largely consistent. Across all indicators, our average observed error (9.2) is very similar to, but slightly smaller than, our average predicted error (9.8). However, like all estimates of uncertainty, our estimates of uncertainty are inherently based on units where we have underlying ground truth data. In regions where ground truth is absent, our uncertainty estimates may not fully capture the true range of predictive errors. Additionally, our estimates assume that the relationships observed in the training data hold in unobserved areas, which may not always be the case due to spatial, temporal, or contextual differences. As a result, while our uncertainty estimates are well-calibrated where validation data exist, their reliability may decrease in areas with limited or no observational data.

Prediction accuracy itself may vary across regions and indicators due to several factors. First, differences in data availability can impact model performance, as regions with sparse or less reliable data may introduce greater uncertainty. Second, regional heterogeneity in digital adoption patterns means that relationships between predictive features and outcomes may differ across contexts, affecting model generalizability. For example, the relationship between internet and Facebook usage can vary across contexts. Third, certain features may have stronger or weaker predictive power depending on local socioeconomic and infrastructural conditions. These factors contribute to variation in both absolute and relative error, influencing the overall accuracy of our estimates.

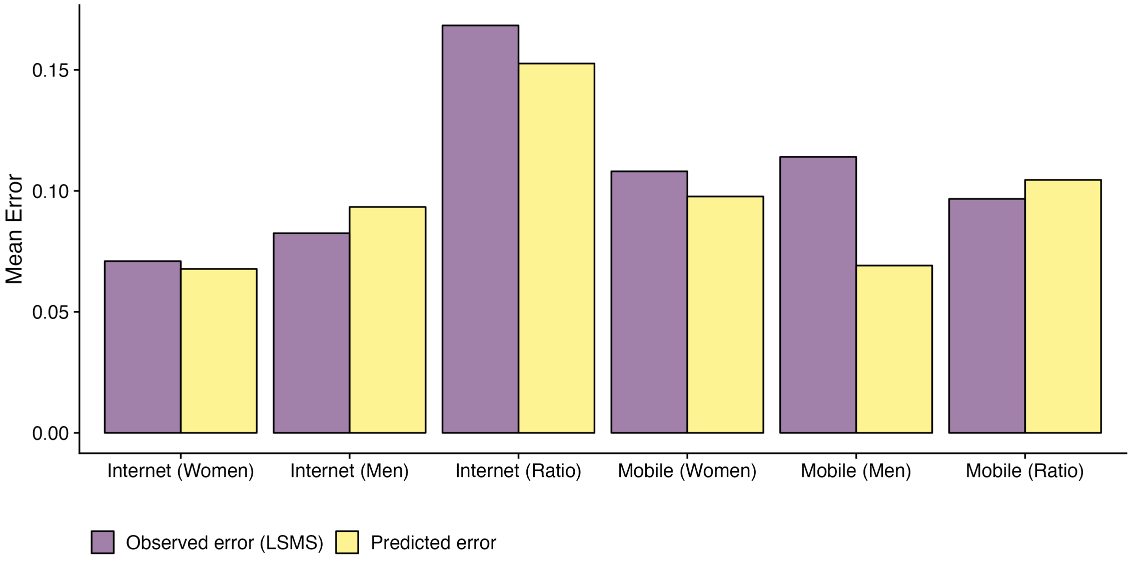

Fig. S13. The mean predicted error and observed error across seven different LSMS countries. Across all indicators, our observed average error (9.2) was slightly smaller than our predicted average error (9.8).

231 ## 4. Maps of subnational gender gaps and adoption levels

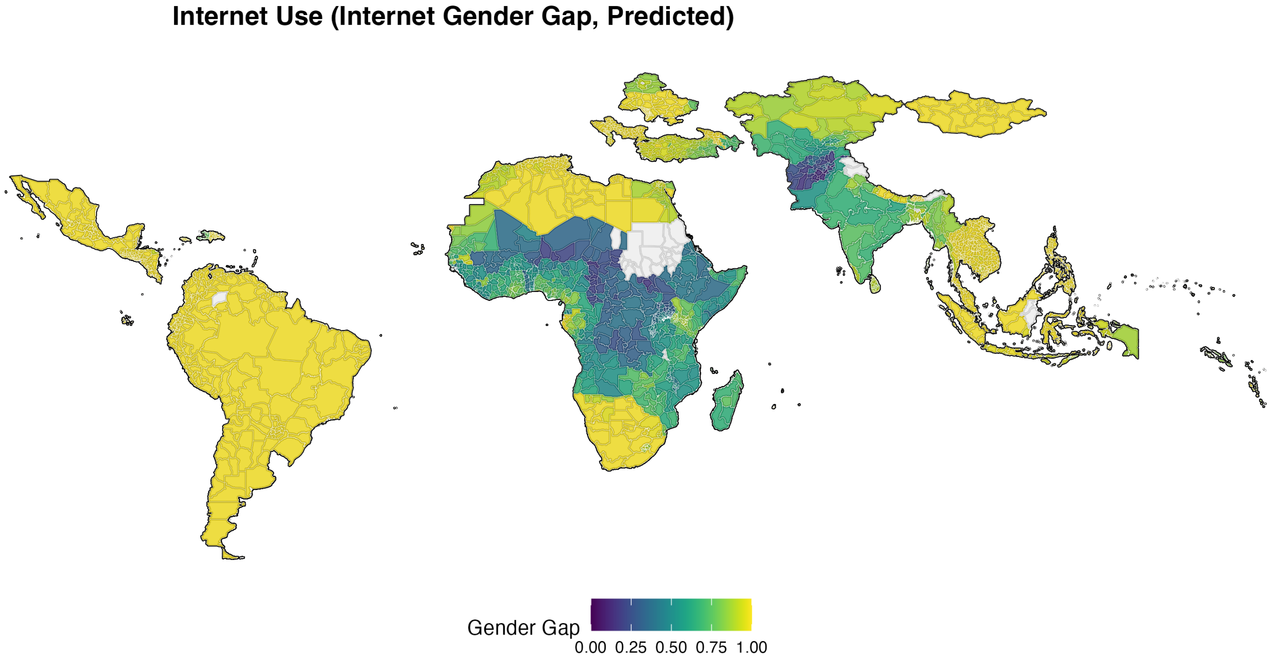

Fig. S14. Estimates of gender gaps in internet use. Map displays estimates from January 2025.

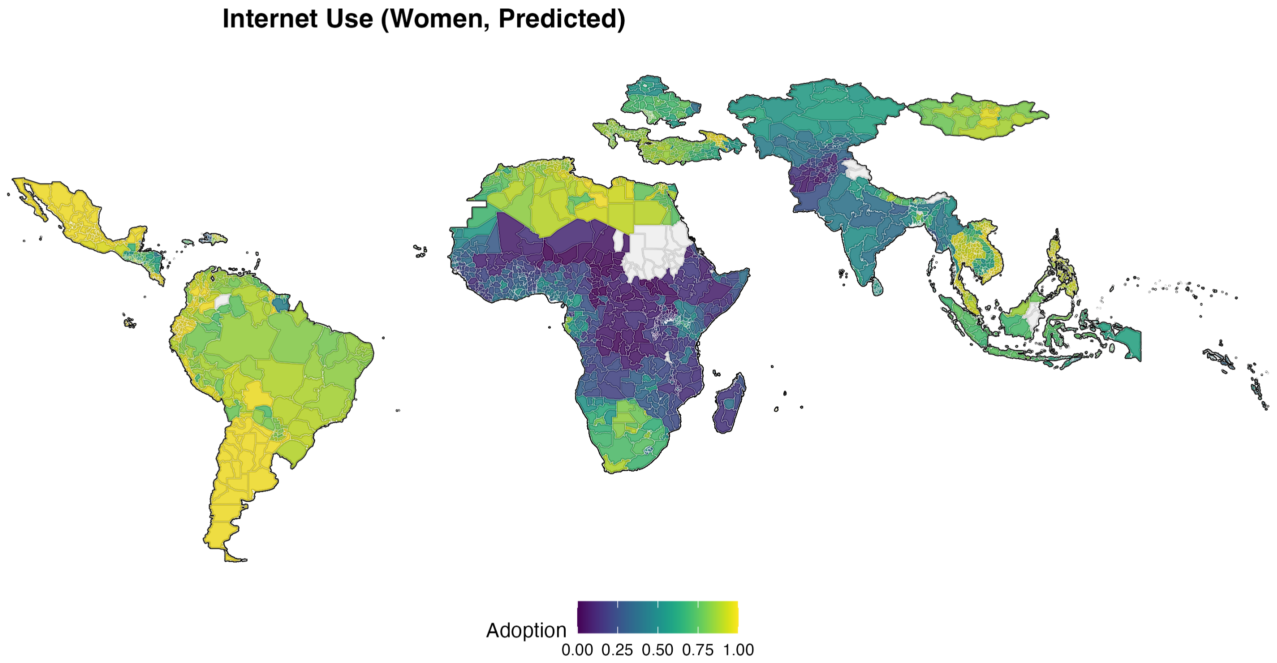

Fig. S15. Estimates of internet adoption for women. Map displays estimates from January 2025.

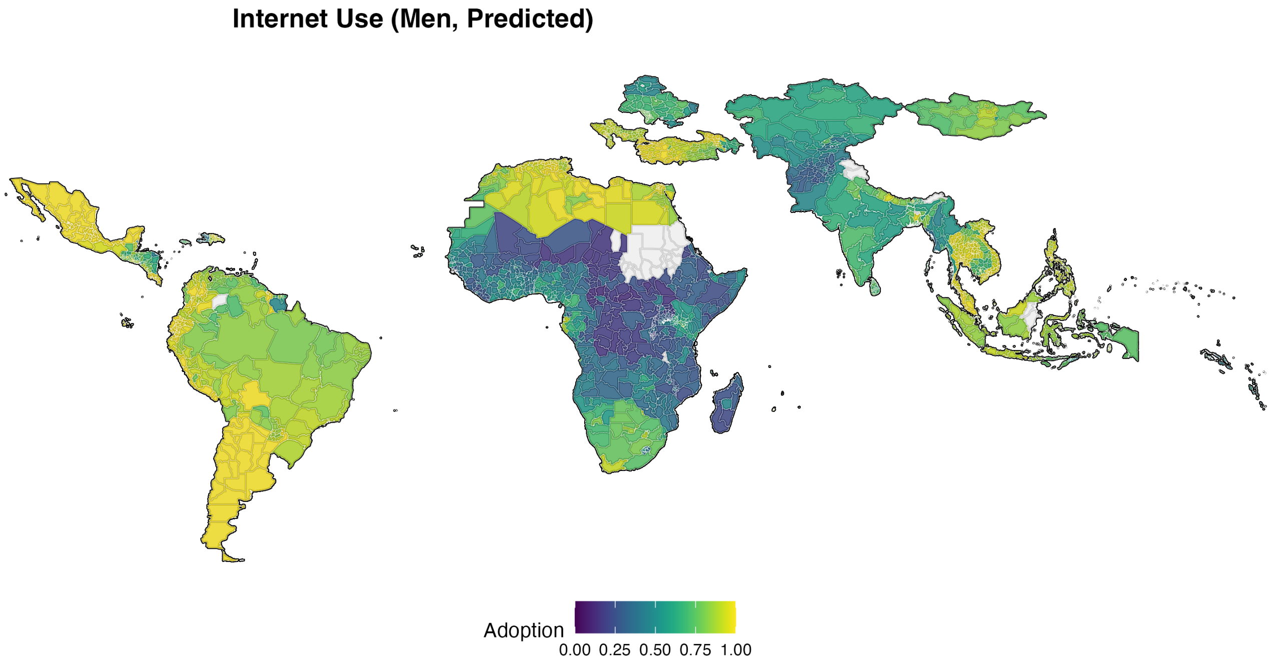

Fig. S16. Estimates of internet adoption for men. Map displays estimates from January 2025.

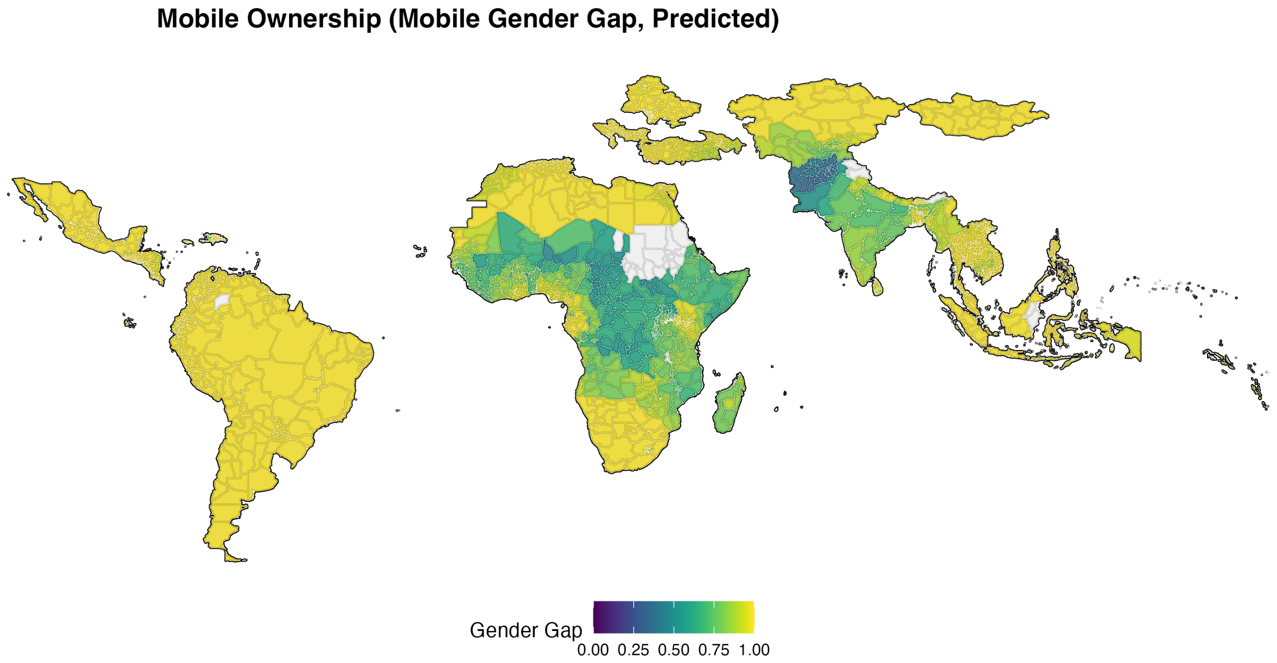

Fig. S17. Estimates of gender gaps in mobile phone ownership. Map displays estimates from January 2025.

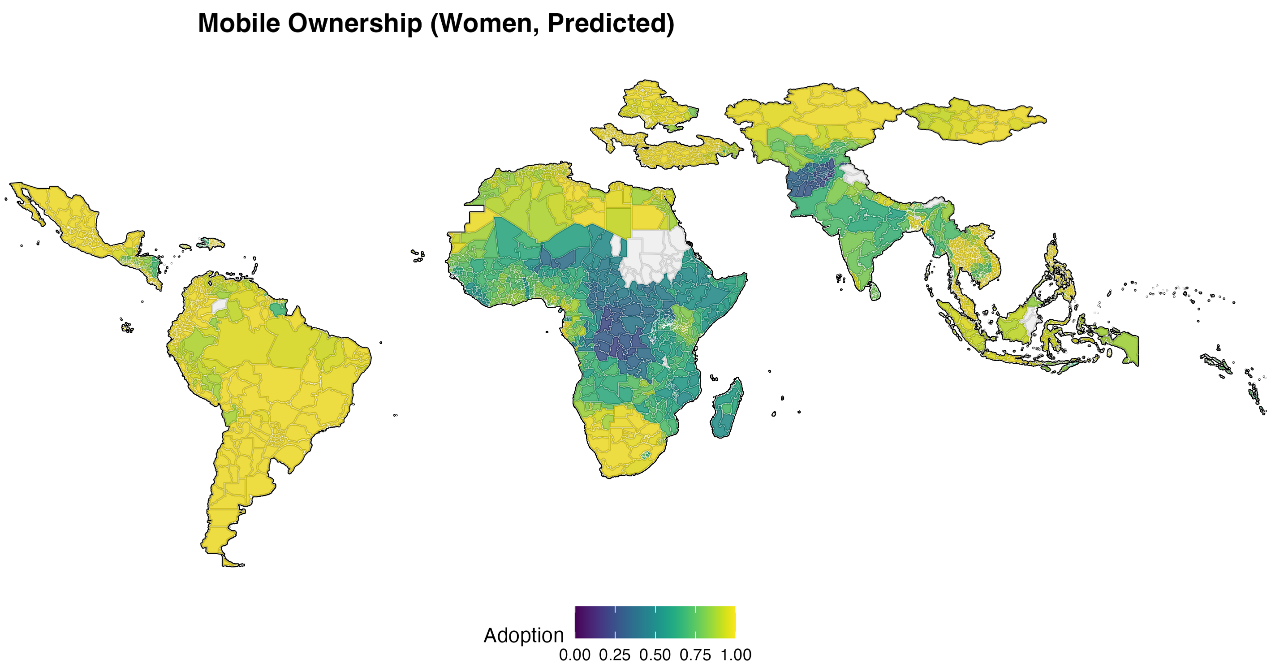

Fig. S18. Estimates of mobile phone adoption for women. Map displays estimates from January 2025.

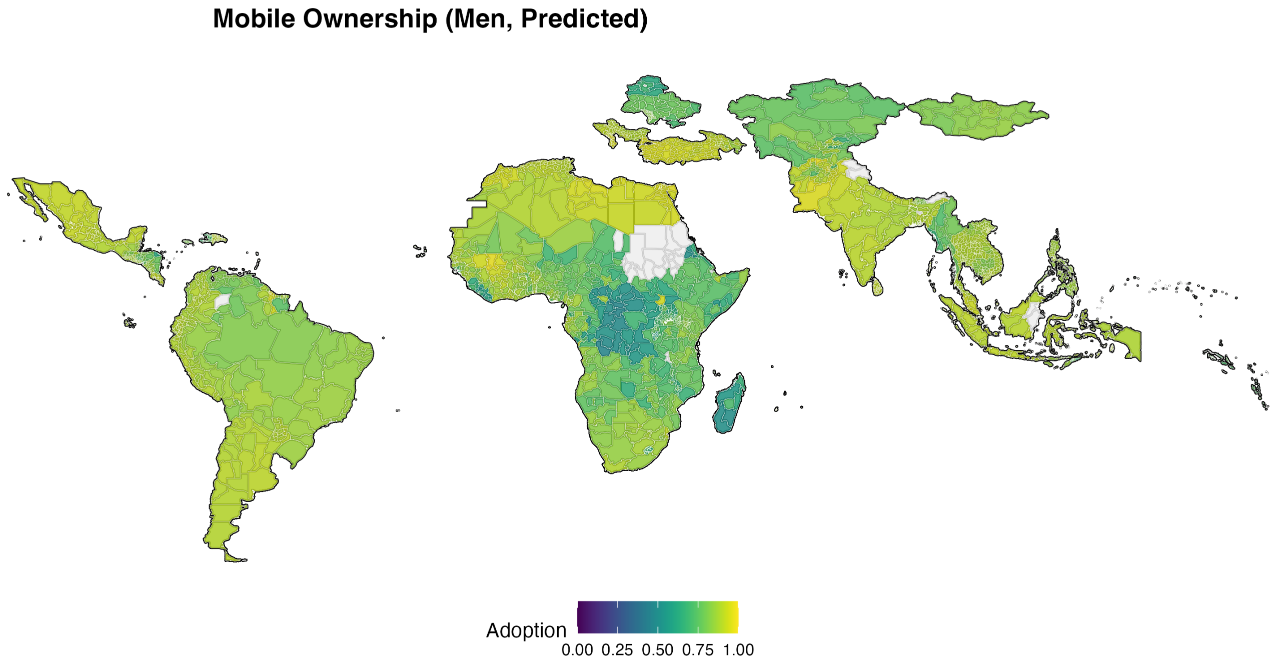

Fig. S19. Estimates of mobile phone adoption for men. Map displays estimates from January 2025.

5. Additional results

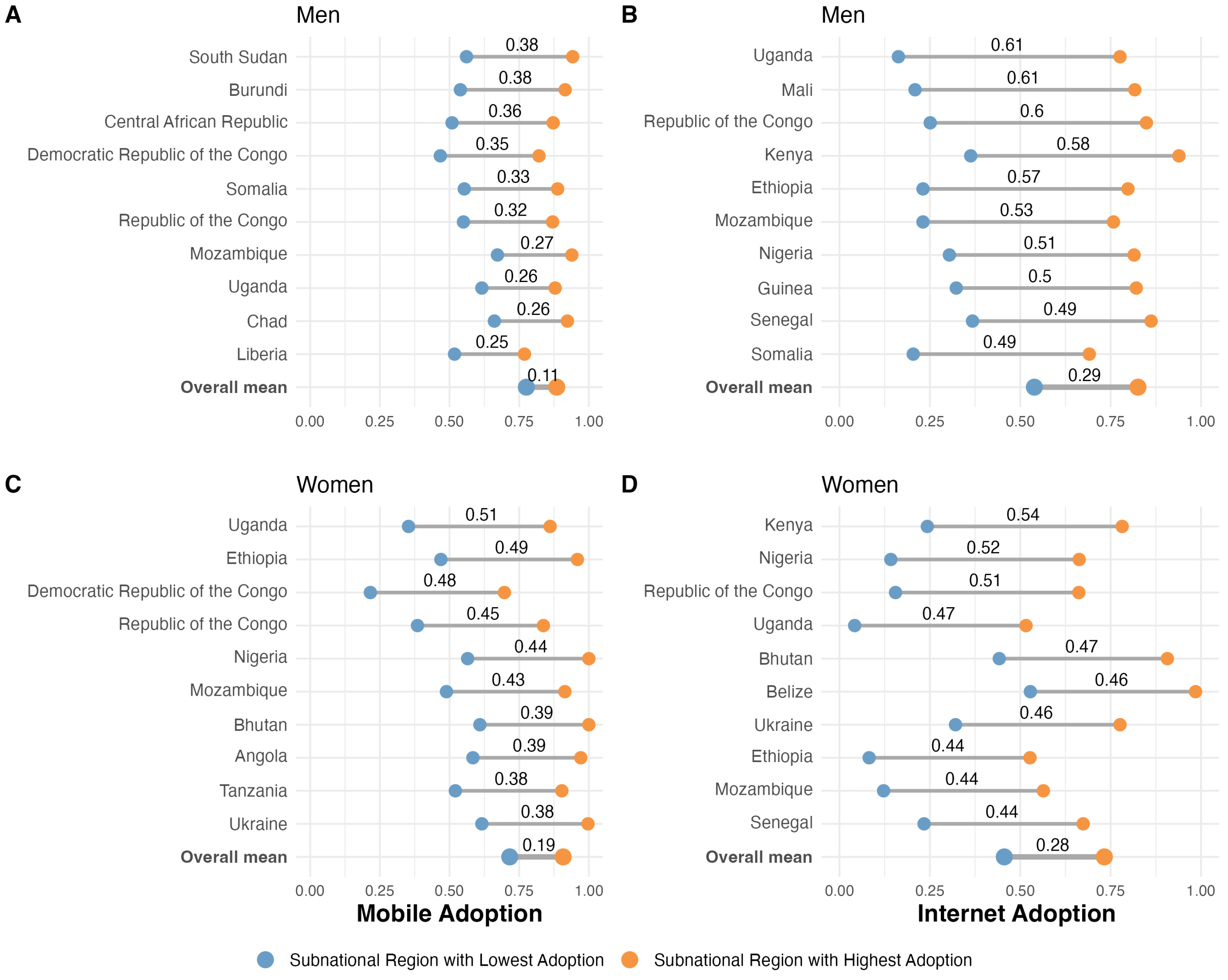

Fig. S20. The top 10 countries with the largest spreads between their lowest and highest subnational unit by gender and digital indicator, organized in descending order by gap size. The bottom bar shows the average top-bottom subnational spread across all countries.

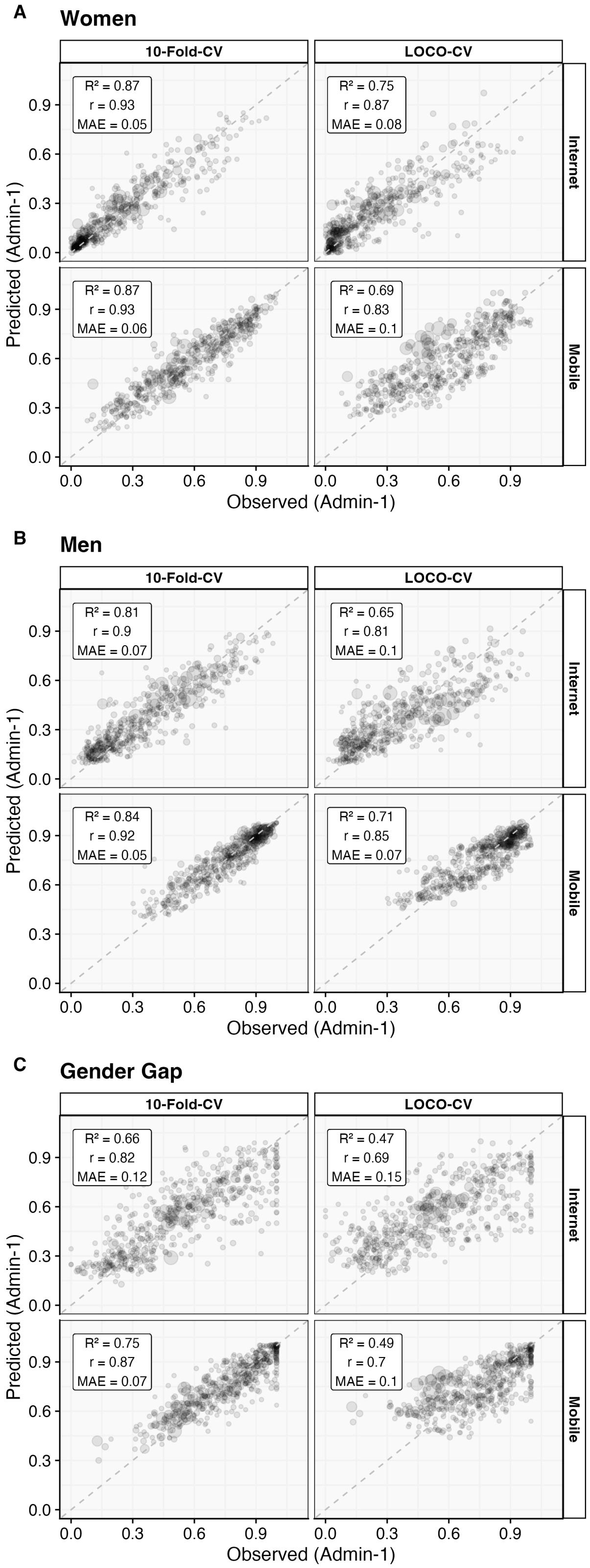

Fig. S21. Scatterplot of observed vs. predicted values under 10-fold cross-validation (10-fold CV) and leave-one-country-out cross-validation (LOCO–CV).

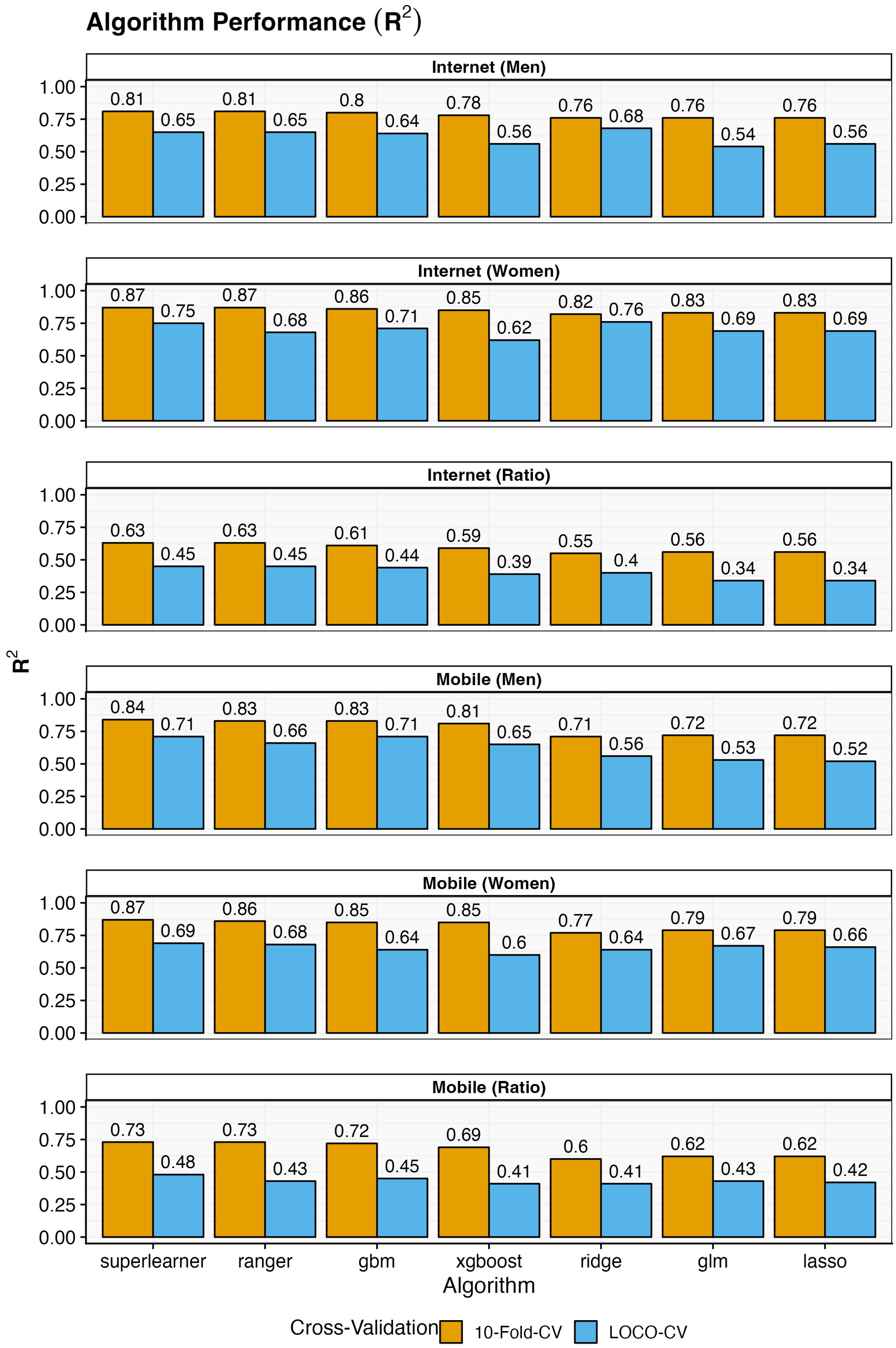

Fig. S22. Performance by algorithm, as measured by R^2 (coefficient of determination) under 10-fold cross-validation (10-fold-CV) and leave-one-country-out cross-validation (LOCO–CV).

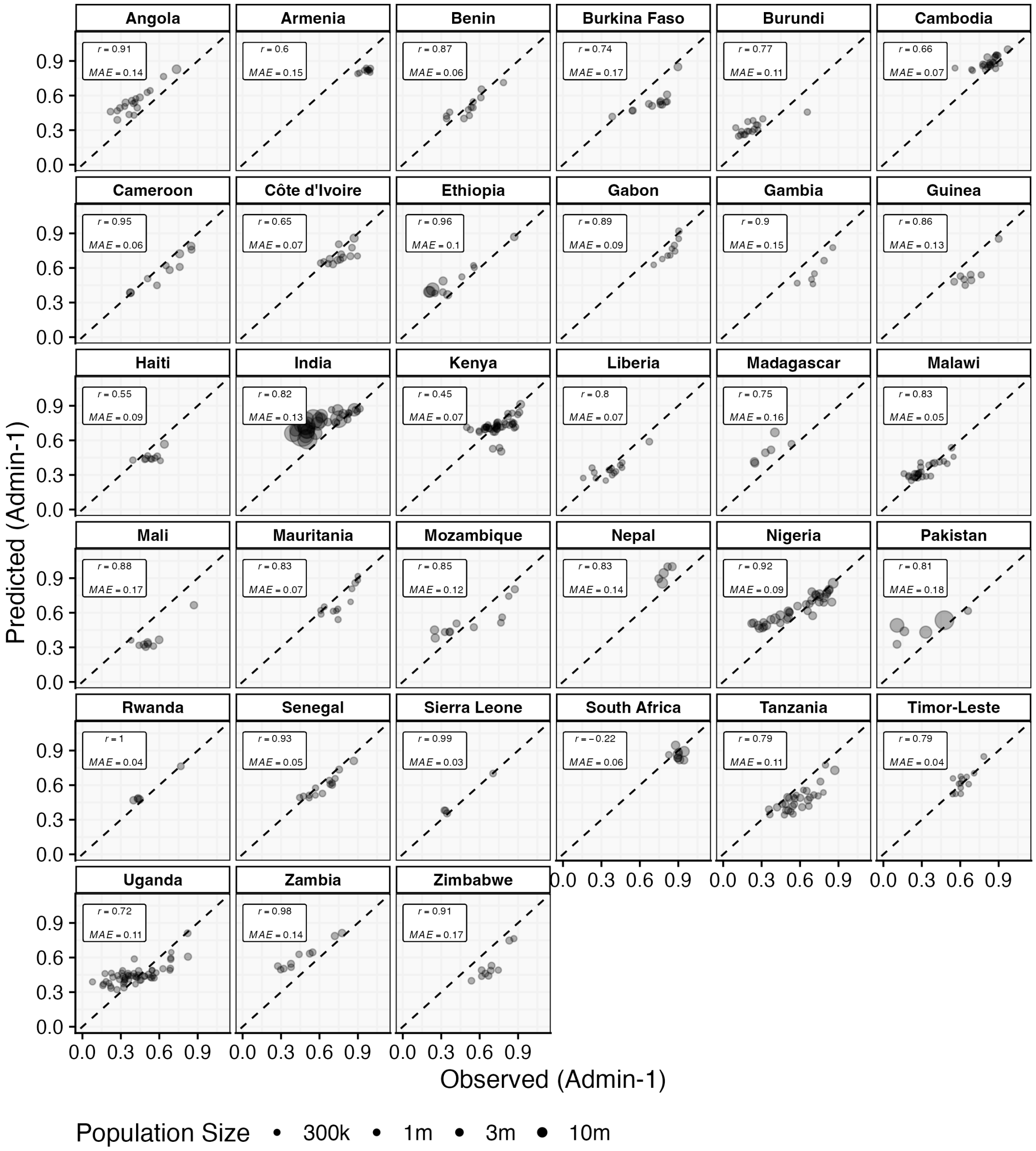

Fig. S23. Scatterplot of observed vs. predicted mobile phone ownership for women by country.

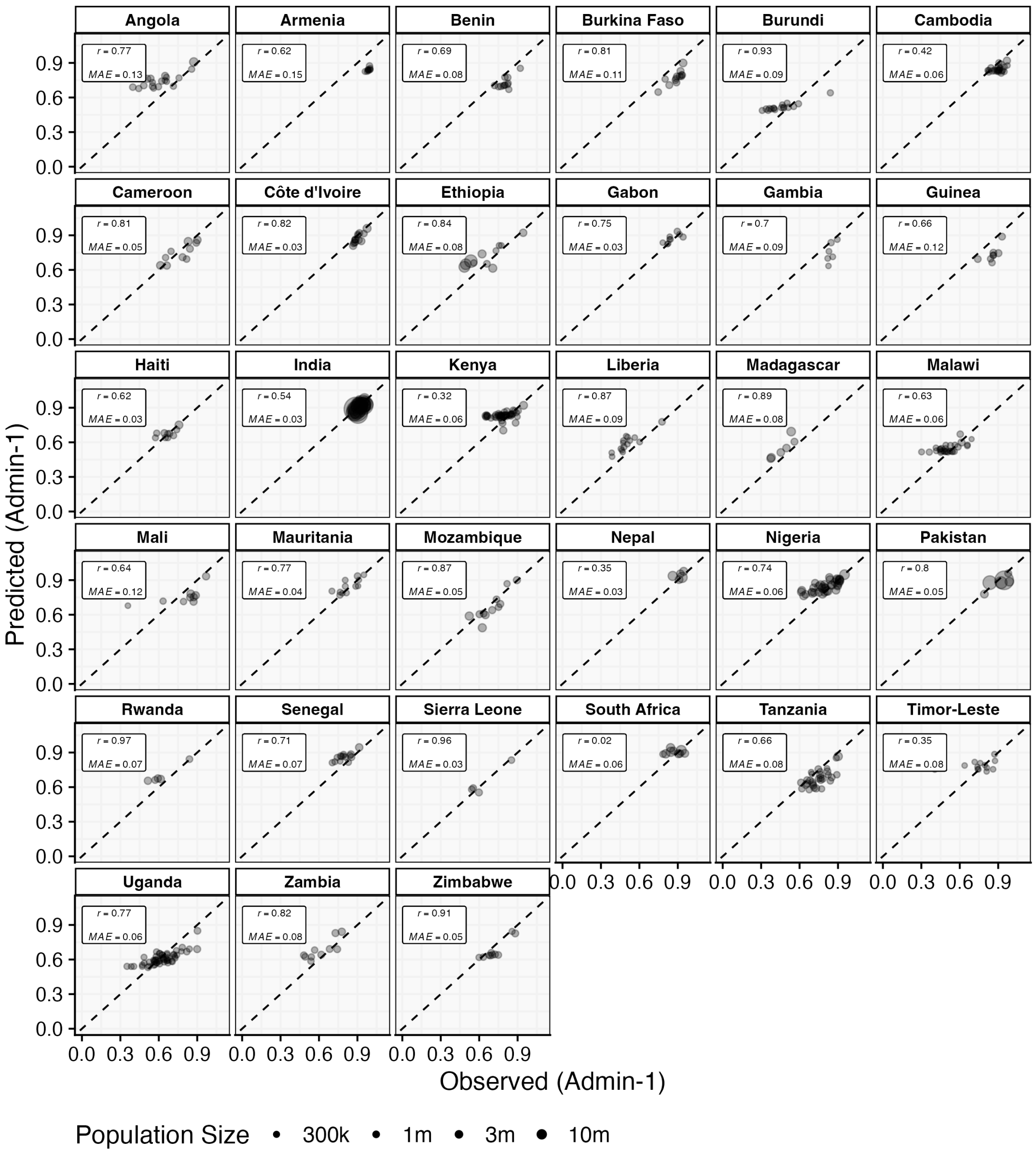

Fig. S24. Scatterplot of observed vs. predicted mobile phone ownership for men by country.

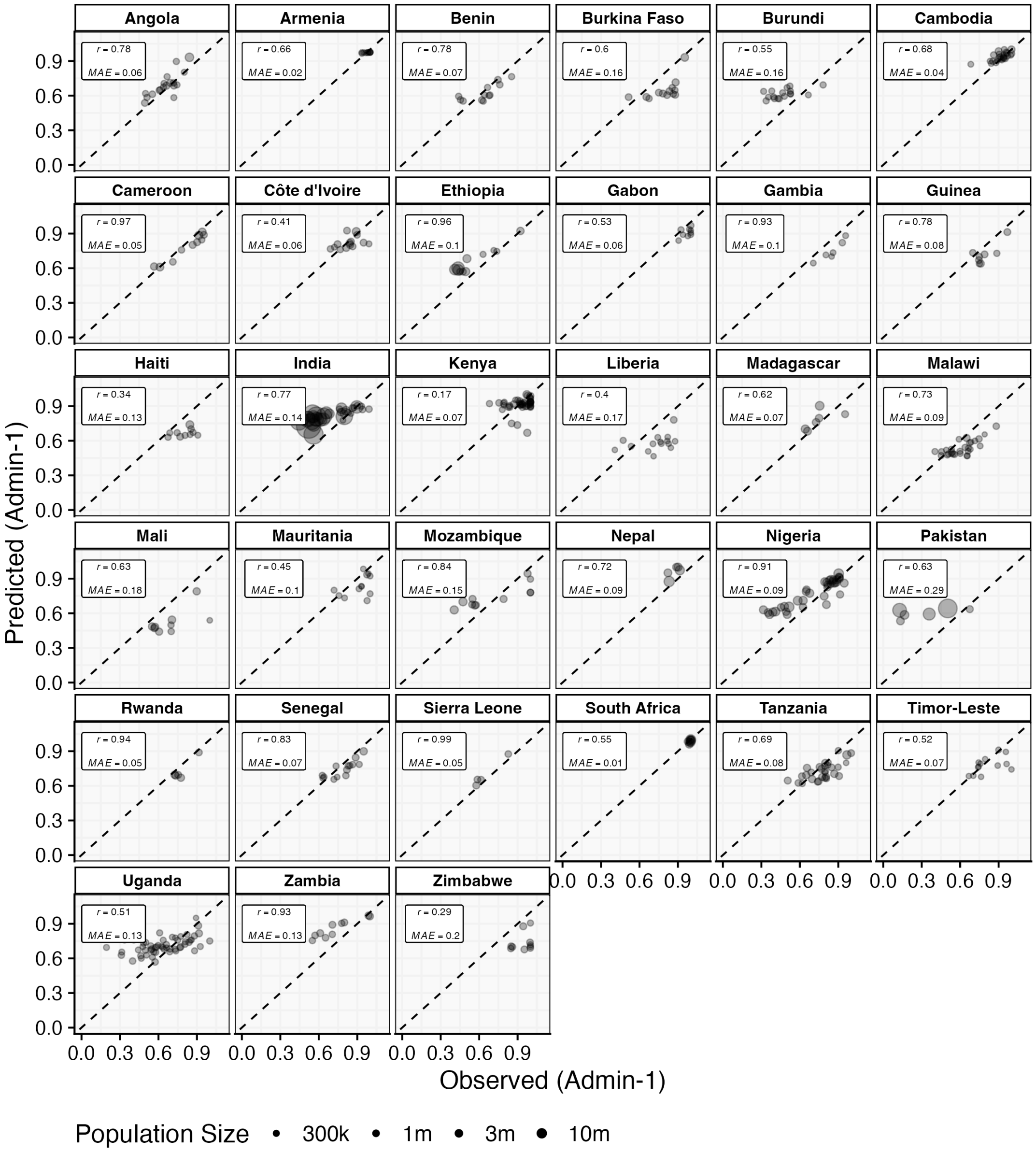

Fig. S25. Scatterplot of observed vs. predicted mobile phone ownership gender gap index by country.

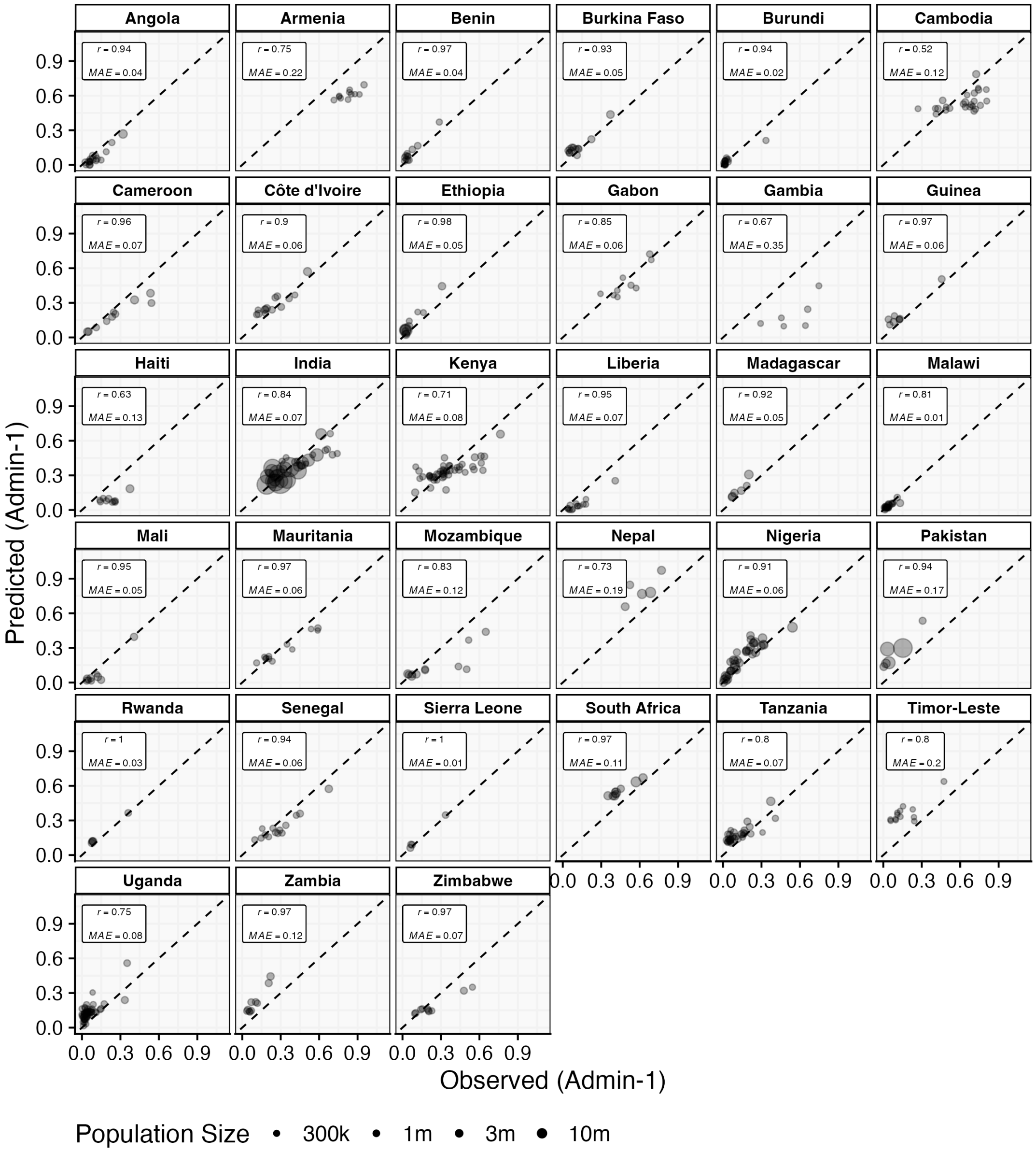

Fig. S26. Scatterplot of observed vs. predicted internet adoption for women by country.

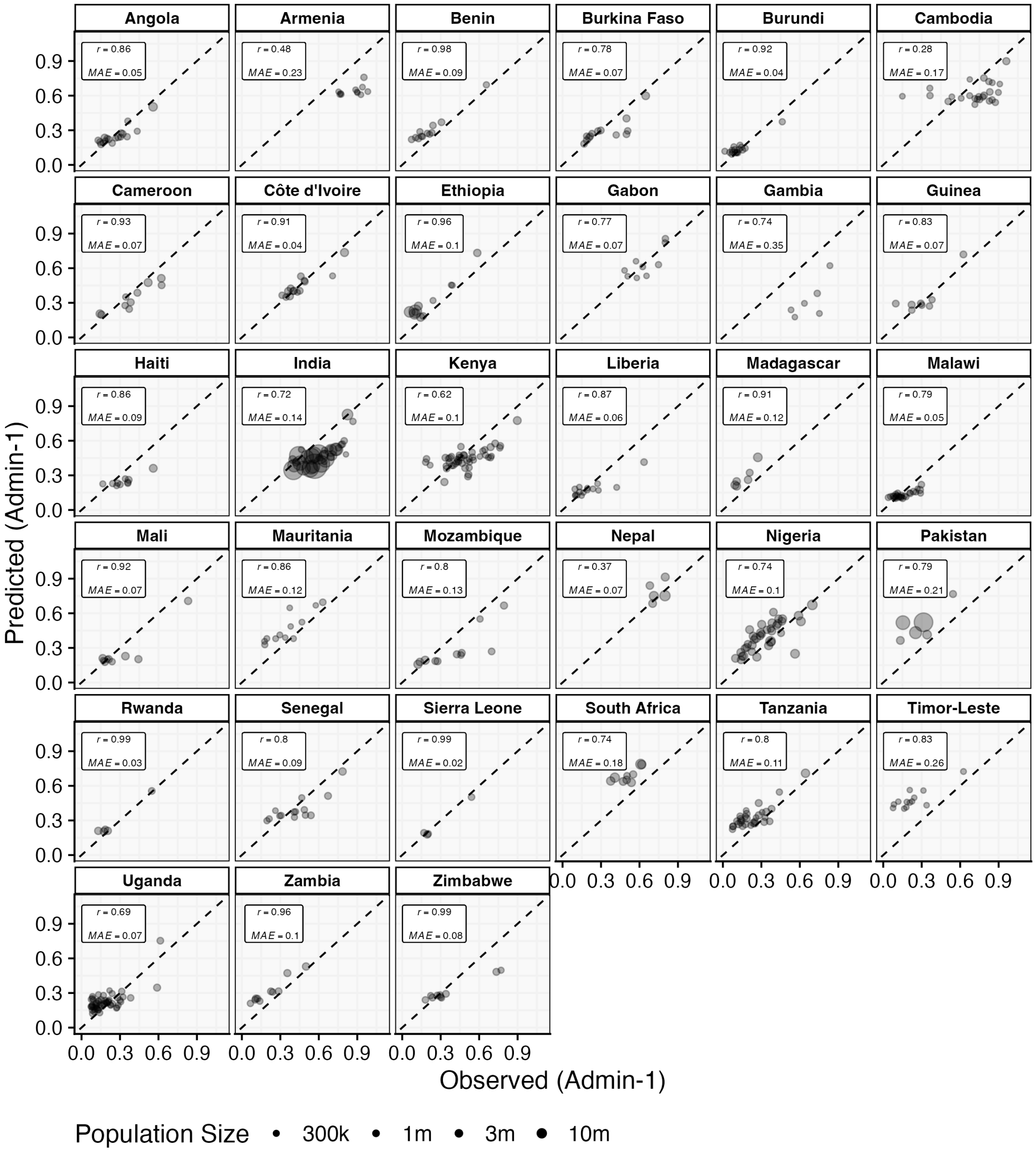

Fig. S27. Scatterplot of observed vs. predicted internet adoption for men by country.

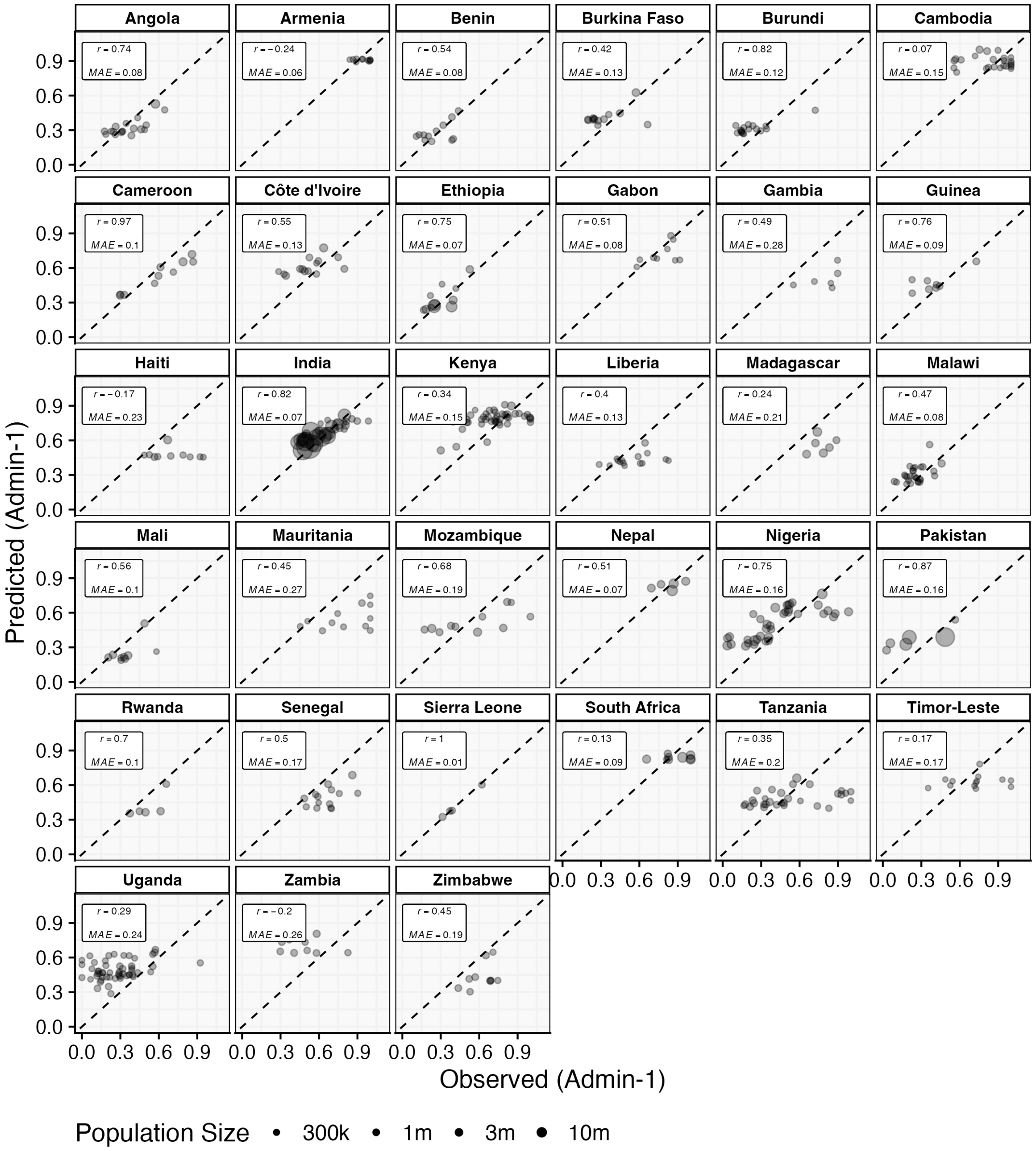

Fig. S28. Scatterplot of observed vs. predicted internet adoption gender gap index by country.

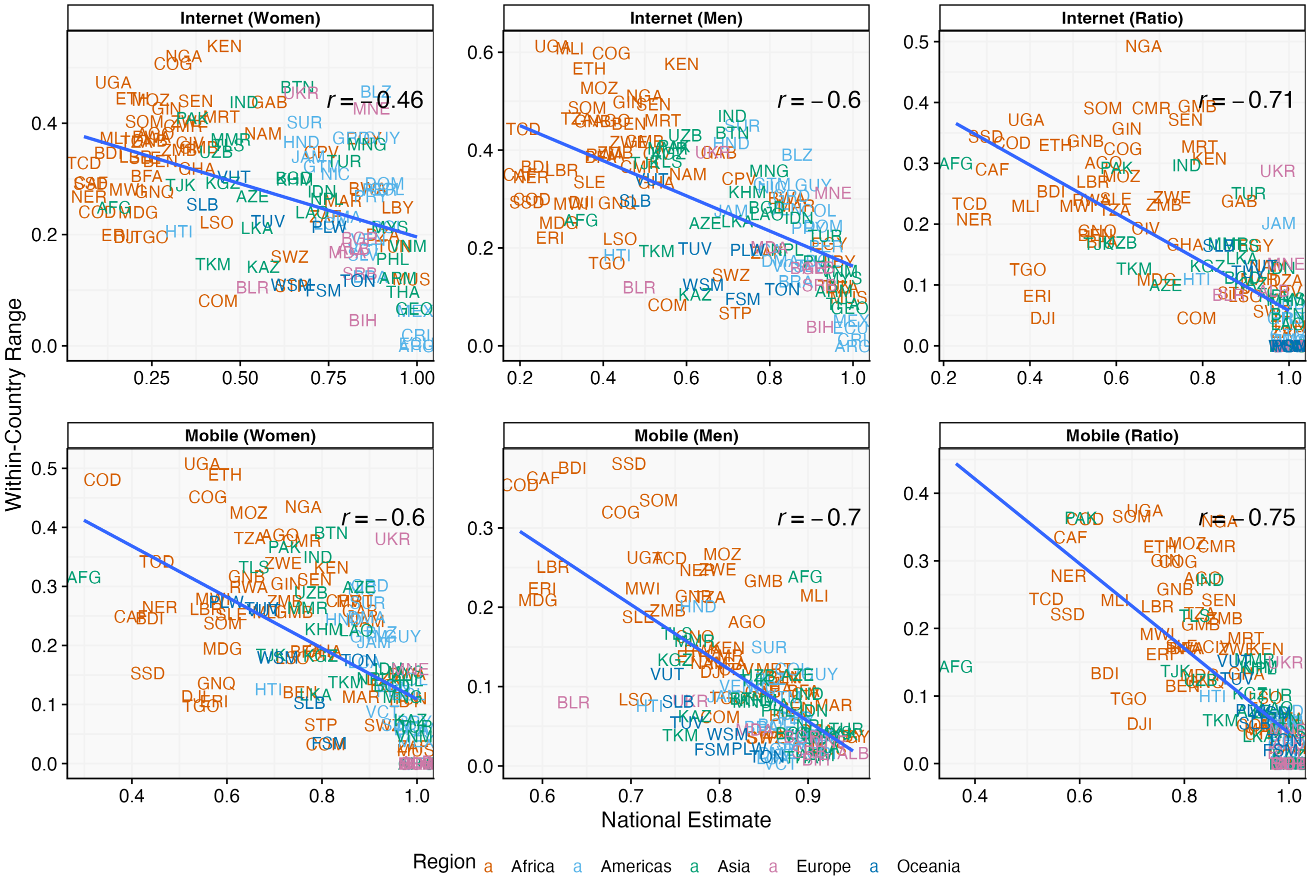

Fig. S29. Relationship between national-level estimates and the within-country spread between the top and bottom region for our six outcomes of interest. Labels show the ISO-3 country codes.

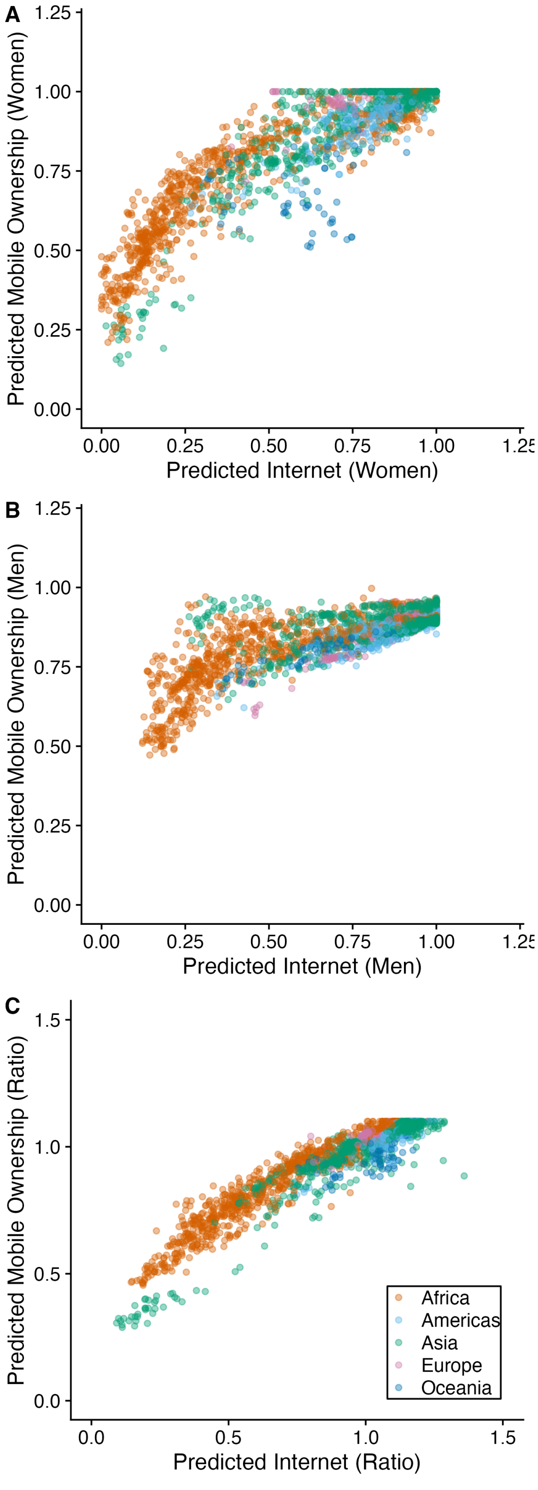

Fig. S30. *(A)* Comparison of predicted internet adoption and mobile phone adoption for women. *(B)* Comparison of predicted internet adoption and mobile phone adoption for men. *(C)* Comparison of predicted internet gender gap index and predicted mobile gender gap index.

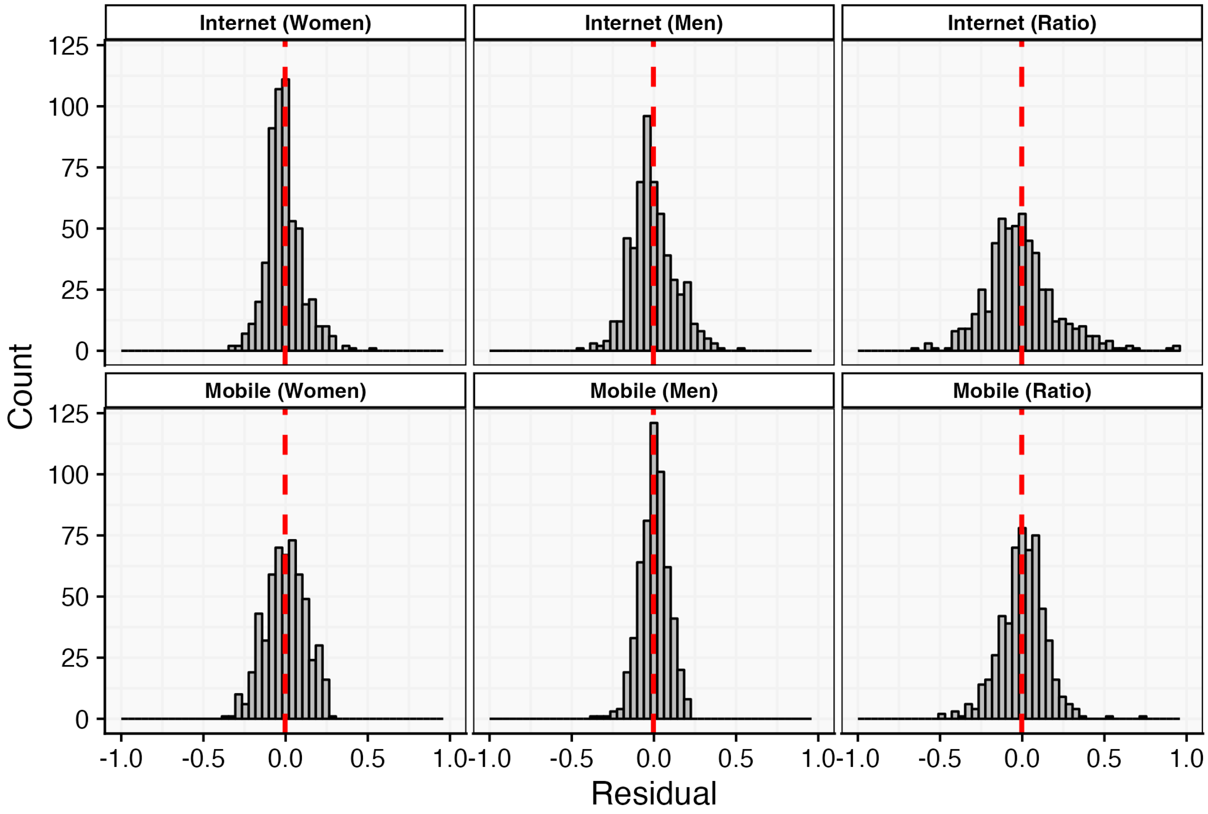

Fig. S31. The residual (observed - predicted) from leave-one-country-out cross-validation (LOCO-CV) for all subnational regions with available ground truth data by indicator. Red dashed line shows mean of residual values.

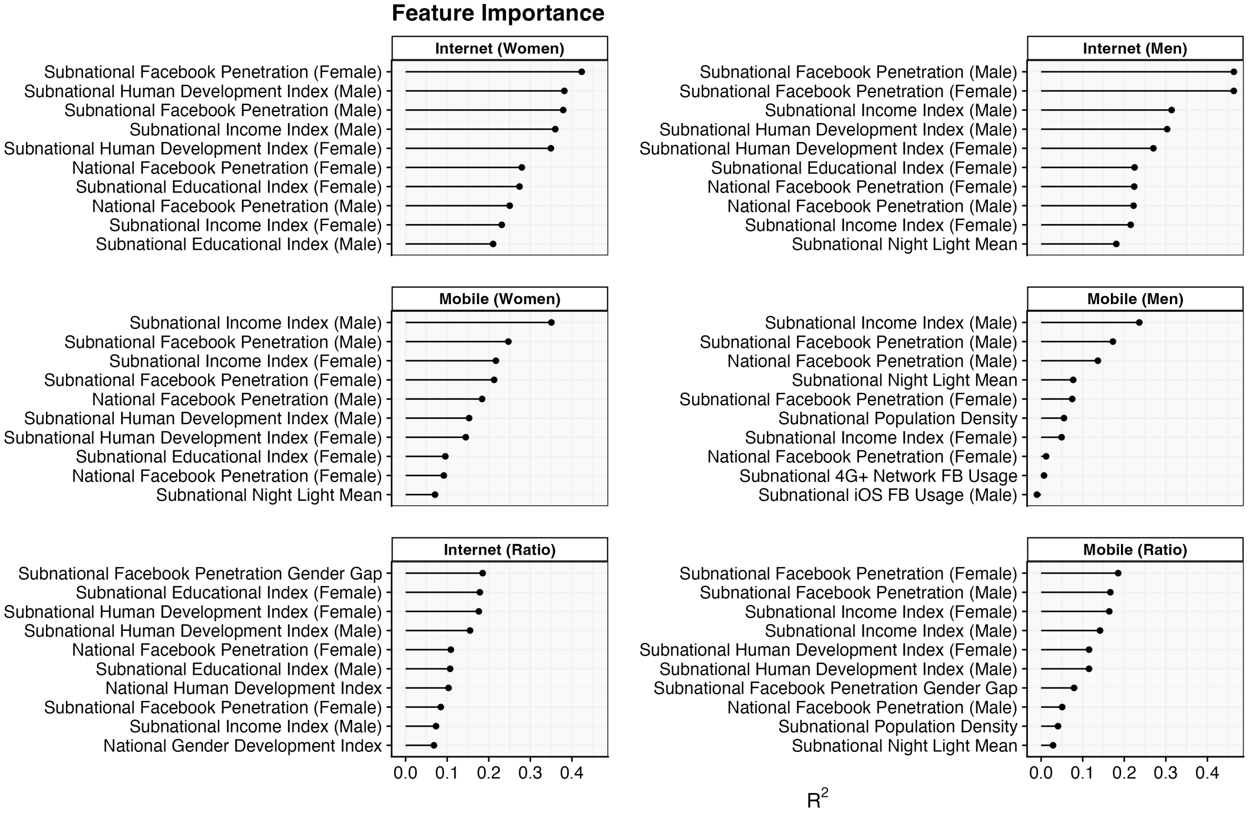

Fig. S32. Feature importance by indicator. Top 10 features are shown. Feature importance is calculated as the R^2 value from a univariate regression.

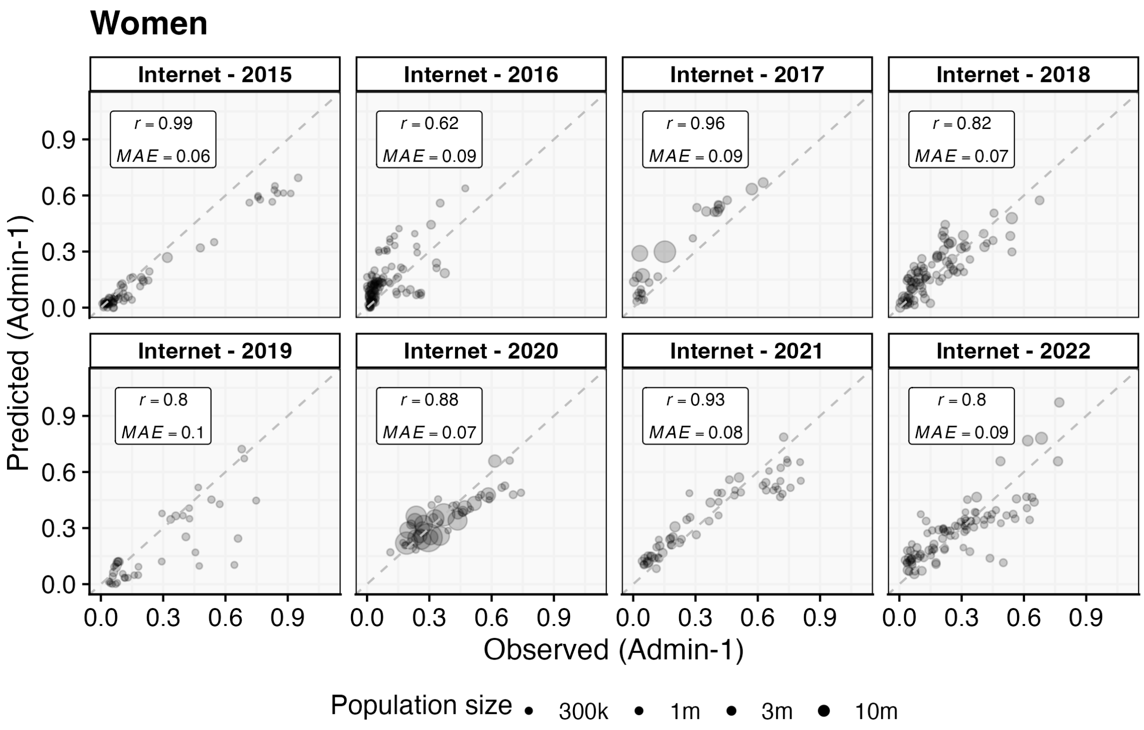

Fig. S33. Predicted vs. observed value for female internet adoption, disaggregated by year. Predictions are based on leave-one-country-out cross-validation (LOCO-CV).

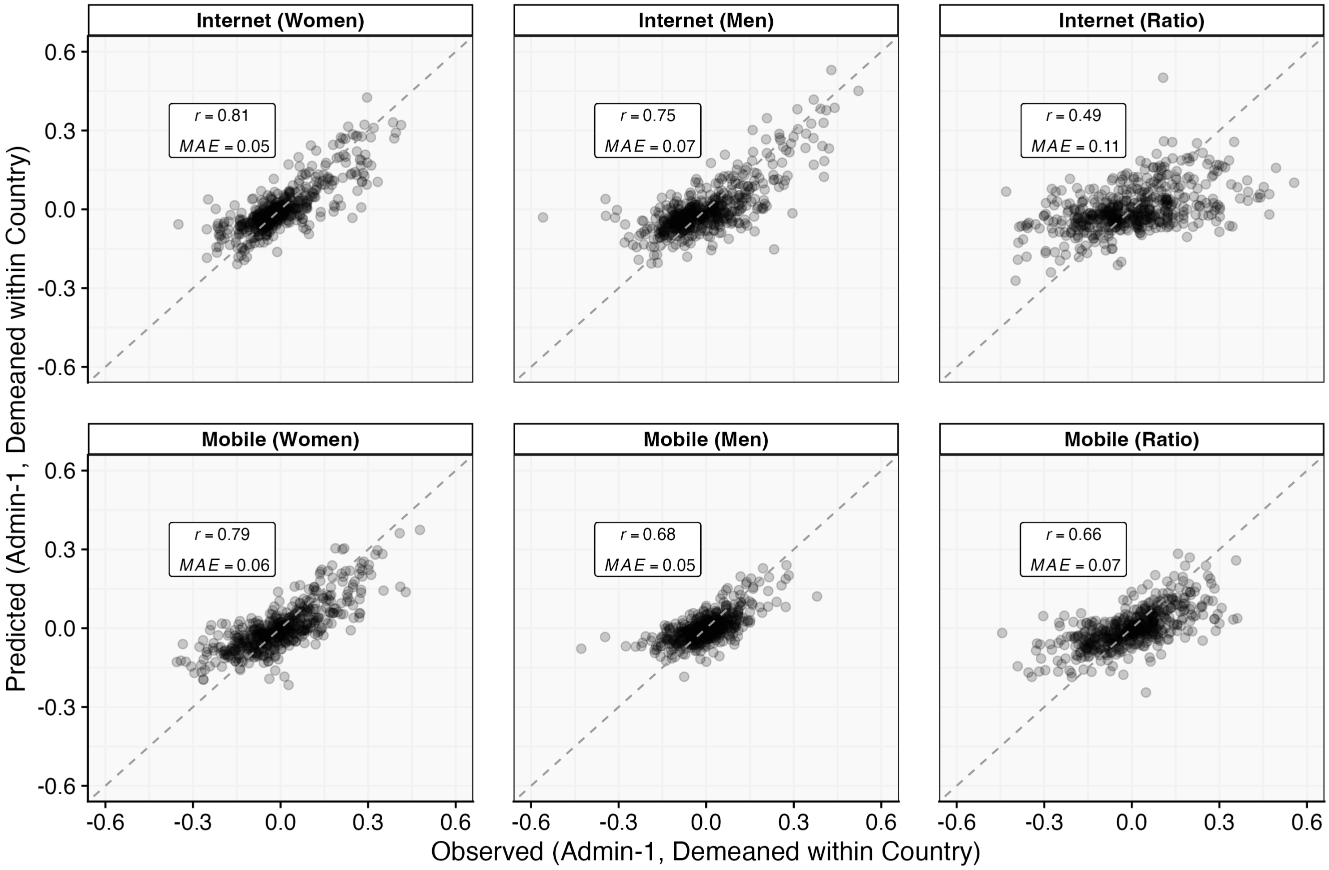

Fig. S34. Predicted vs. observed values, demeaned by country. Predictions are based on leave-one-country-out cross-validation (LOCO-CV).

6. REFORMS Checklist

The REFORMS checklist, developed collaboratively by 19 researchers across the social, computer, and physical sciences, is a resource for promoting transparency and reproducibility in machine learning science (14). We complete this checklist to promote methodological transparency.

6A. Study Goals.

1a. *State the population or distribution about which the scientific claim is made.*

This study makes scientific claims about levels and gaps of digital adoption in 2,075 subnational regions across 117 different low- and middle-income countries (LMICs) over the period 2015 through 2025.

1b. *Describe the motivation for choosing this population or distribution.*

We focus on LMICs settings as overall levels of digital penetration are low and gender gaps disfavoring women are high. We focus on the first subnational level as subnational variation represents a substantial component of overall inequality, especially in countries with the lowest levels of development.

1c. *Describe the motivation for the use of ML methods in the study.*

We use machine learning methods to predict levels and gaps of internet adoption and mobile phone ownership by gender. Machine learning is an appropriate choice for this task because our primary focus is on predictive accuracy rather than model interpretation. Additionally, machine learning algorithms are better suited for handling the complex interactions and non-linear relationships among features. Given that we are interested in estimating trends in digital adoption over time, machine learning models are helpful in their flexibility to capture temporal trends and year-specific shifts in adoption patterns.

6B. Computational Reproducibility.

2a. *Describe the dataset used for training and evaluating the model and provide a link or DOI to uniquely identify the dataset.*

The dataset used to train and evaluate the model contains columns corresponding to model features or outcomes and rows corresponding to different subnational units for each year from 2015 through 2025. The dataset is available from: https://doi.org/10.17605/OSF.IO/5E8WF. For more information on how each feature was constructed, see Section 1.

2b. *Provide details about the code used to train and evaluate the model and produce the results reported in the paper along with link or DOI to uniquely identify the version of the code used.*

To train our machine learning algorithms, we used an ensemble Superlearner (7). We first fit separate models for each of our six outcomes separately using the SL3 package in R (15). All code to train these models is available in our replication package: https://doi.org/10.17605/OSF.IO/5E8WF.

2c. *Describe the computing infrastructure used.*

All computations were carried out on 2023 MacBook Pro with an Apple M2 Pro chip, 16GB memory, and Sonoma 14.1 operating system. We use R version 4.3.1 and package versions recorded in the README file of the replication package.

2d. *Provide a README file which contains instructions for generating the results using the provided dataset and code.*

A README file containing instructions for generating all estimates, figures, and tables presented in the paper is available from: https://doi.org/10.17605/OSF.IO/5E8WF.

2e. *Provide a reproduction script to produce all results reported in the paper.*

The replication scripts for generating all figures and code in the paper are available here: https://doi.org/10.17605/OSF.IO/5E8WF

6C. Data Quality.

3a. *Describe source(s) of data, separately for the training and evaluation datasets, along with the time when the dataset(s) are collected, the source and process of ground-truth annotations, and other data documentation.*

The data for this study come from several different sources. First, our ground truth data came from a series of 33 Demographic and Health Surveys (DHS). The survey datasets were obtained directly from the DHS Program's website on March 1, 2024. To construct our features, we combine data from the following sources: Worldpop, NASA Earth Observation Group, Global Data Lab, and the Facebook Marketing API. For more details on data processing, see Section 1.

In addition, we used data two auxiliary sources for validation: Multiple Indicator Cluster Surveys (MICS) and LSMS (Living Standards Measurement Study). These data were obtained in May 2025.

3b. *State the distribution or set from which the dataset is sampled (i.e., the sampling frame).*

The dataset used for our analysis is structured where each observation (row) corresponds to a different subnational unit by year. Each feature (column) corresponds to a characteristic of a subnational unit. The ground truth rates of digital adoption in the dataset are calculated based on microdata from DHS surveys, which typically use a stratified two-stage cluster design (16).

All features are temporally aligned to their respective year, effectively creating a longitudinal panel spanning 2015–2025.

3c. *Justify why the dataset is useful for the modeling task at hand.*

This dataset is appropriate for modeling subnational levels and gaps of internet and mobile phone adoption as it includes, at the admin-1 level, both ground truth measures of digital adoption and a carefully curated set of predictors associated with digital adoption and gaps.

6D. Data Pre-processing.

4a. *Describe whether any samples are excluded with a rationale for why they are excluded.*

We only make predictions for LMICs where Facebook MAU data is available. No Facebook MAU counts are available for the following countries: American Samoa, China, Cuba, Fiji, French Southern Territories, Kosovo, Marshall Islands, Mayotte, North Korea, Papua New Guinea, Russia, Réunion, Saint Helena, Ascension, and Tristan da Cunha, Seychelles, Sudan, Western Sahara.

4b. *Describe how impossible or corrupt samples are dealt with.*

There are no impossible or corrupt samples.

4c. *Describe all transformations of the dataset from its raw form to the form used in the model, for instance, treatment of missing data and normalization—preferably through a flow chart.*

For most subnational units in our analysis, we have no missing predictors. When data is missing, we impute using the value from nearest non-missing year within that subnational unit. If data is missing for a feature for all years, we use the median value within the continent. An overview of data processing is available in Section 1.

6E. Modeling.

5a. *Describe, in detail, all models trained.*

We fit six different ensemble Superlearner models, one for each outcome of interest. Each ensemble Superlearner algorithm combines multiple predictions from a library of individual machine learning algorithms. Specifically, we include the following individual machine learning algorithms in our ensemble library:

- Generalized Linear Model (GLM)
- Lasso Regression
- Ridge Regression
- Elastic Net Regression
- Polynomial Spline Regression
- Random Forests
- Gradient Boosted Machine (GBM)
- Extreme Gradient Boosting (XGB)

Temporal variation was captured through year-specific features and the inclusion of a relative-year covariate, allowing the model to learn both spatial and temporal patterns in adoption.

5b. *Justify the choice of model types implemented.*

We chose to use an ensemble Superlearner model to enhance predictive accuracy by leveraging the strengths and smoothing over limitations of each individual model. The choice of individual machine learning algorithms was driven by their ability to handle various data characteristics, such as non-linear relationships, interactions, and high-dimensional data. We selected a diverse set of machine learning algorithms for our ensemble library to capture a wide range of data characteristics, including non-linear relationships, interactions, and high-dimensional features.

5c. *Describe the method for evaluating the model(s) reported in the paper, including details of train-test splits or cross-validation folds.* The models were evaluated using two types of cross-validation:

- **10-fold cross-validation**: The data were randomly split into ten folds, with nine folds used for training and one for testing, repeated for each fold. This gives a sense of how the model would perform for countries with subnational ground truth estimates of adoption available for sum, but not all, admin-1 units.

- **Leave-one-country-out cross-validation (LOCO-CV)**: Data from one country were held out at a time, the model was trained on the remaining countries, and predictions were made for the subnational units in the held-out country. This process was repeated for each country, ensuring that no data from the test country influenced the training. This strategy is more conservative, and gives insight into how the model would perform on a country where we have some subnational ground truth estimates of adoption.

We further evaluated the models by benchmarking against external estimates from MICS and LSMS surveys. This allows us to assess the performance of the model against independent estimates. To evaluate the models' ability to capture trends over time, we use countries with repeated surveys (from DHS, MICS, and LSMS) to compare our predicted change over time with observed changes over time, with the caveat that observed changes over time may reflect true change or artifacts of sampling variation and survey inconsistencies. Overall, our results demonstrate that our models capture changes in adoption reasonably well.

5d. *Describe the method for selecting the model(s) reported in the paper.*

We report both the performance of the ensemble Superlearner algorithms and the individual machine learning algorithms in Fig. S22. However, in the main text, we only show predictions from our best-performing Superlearner algorithms. The ensemble Superlearner algorithm is a weighted combination of predictions from each individual machine learning algorithm, and has the best overall performance across all indicators. Weights are calculated using a non-negative least squares (NNLS) regression meta-learner and shown in Section 3A.

5e. *For the model(s) reported in the paper, specify details about the hyperparameter tuning.*

Hyperparameter tuning was performed ad-hoc for each individual model within the Superlearner framework using cross-validation. Specific tuning processes were applied as follows:

- Random forests: Number of trees
- Gradient boosting machines: Learning rate, number of trees, and tree depth

5f. *Justify that model comparisons are against appropriate baselines.*

Our model baseline is a generalized linear model (GLM), fit within our Superlearner framework. This simple model is a standard and appropriate baseline that allows us to assess the performance of our ensemble superlearner algorithm.

6F. Data Leakage.

6a. *Justify that pre-processing and modeling steps only use information from the training dataset (and not the test dataset).*

Pre-processing and modeling steps were strictly limited to using information from the training dataset. The LOCO-CV approach ensured that no data from the test countries were included in the training process, maintaining strict data separation.

6b. *Describe methods used to address dependencies or duplicates between the training and test datasets.*

Dependencies and duplicates were managed by ensuring that each country's data were treated independently during the LOCO-CV process. This approach inherently avoids any overlap or dependencies between training and test datasets.

6c. *Justify that each feature or input used in the model is legitimate for the task at hand and does not lead to leakage.*

All features used in our models were carefully selected to avoid data leakage. None of the features in our models are proxies for the outcome, and were all measured independently of the outcomes.

6G. Metrics and Uncertainty.

7a. *State all metrics used to assess and compare model performance. Justify that the metric used to select the final model is suitable for the task.*

For our primary model performance metric, we use the coefficient of determination, R^2. This metric is valuable because it quantifies the proportion of variance in the dependent variable explained by the model, providing an absolute measure of model fit. Additionally, we use the mean absolute error (MAE) to assess the average magnitude of prediction errors, offering a direct interpretation of prediction accuracy in the units of the dependent variable. Finally, we use the Pearson correlation coefficient (r), which measures the linear correlation between observed and predicted values, showing how well the predicted values follow the trend of the observed ground truth data. These metrics collectively ensure a comprehensive evaluation of the model's performance, capturing both fit quality and prediction accuracy.

7b. *State uncertainty estimates and give details of how these are calculated.*

To estimate uncertainty for each subnational unit, we regress the absolute residuals against all observable variables for units with ground truth data. We use non-negative least squares regression to ensure estimated absolute residuals are positive. We then use this model to predict the absolute residual size for all subnational units.

7c. *Justify the choice of statistical tests (if used) and a check for the assumptions of the statistical test.*

We do not conduct any statistical tests.

6H. Generalizability and Limitations.

8a. *Describe evidence of external validity.*

Our models are calibrated using ground truth data from DHS surveys. It is possible that countries that do not have DHS surveys differ in important ways from the countries that do have DHS surveys. Given the availability of ground truth coverage from 33 different countries, this is unlikely to drastically affect our external validity. Further, comparison with independent ground truth estimates of digital adoption from LSMS surveys and MICs surveys at the subnational-level revealed strong agreement in countries with no DHS surveys.

8b. *Describe contexts in which the authors do not expect the study's findings to hold.*

In settings where digital adoption is especially high, the relationship between Facebook use and internet use might be more heterogeneous. Our models' performance in high-adoption settings is still reasonably accurate, but it performs best at estimating internet adoption levels in lower penetration settings. This is reflected in the higher uncertainty estimates in high-adoption settings.

In addition, if the correlation between Facebook penetration and digital adoption weakens in the future, the effectiveness of our methods may decline. However, the rise of other social media platforms could provide alternative or complementary data sources, if these user counts become publicly available.

Finally, estimating trends in digital adoption over time is inherently challenging. Our validation exercises show promising alignment between estimated and observed trends, especially given data limitations. However, more reliable ground truth estimates of trends over time would allow for more rigorous validation and would strengthen confidence in the models' ability to capture temporal dynamics.

References

1. M Araujo, Y Mejova, I Weber, F Benevenuto, Using Facebook Ads Audiences for Global Lifestyle Disease Surveillance: Promises and Limitations in *Proceedings of the 2017 ACM on Web Science Conference*, WebSci '17. (ACM, New York, NY, USA), pp. 253–257 (2017).
2. WorldPop, Open Spatial Demographic Data and Research (https://www.worldpop.org/) (2023).
3. J Smits, I Permanyer, The Subnational Human Development Database. *Sci. Data* **6**, 190038 (2019).
4. CD Elvidge, M Zhizhin, T Ghosh, FC Hsu, J Taneja, Annual Time Series of Global VIIRS Nighttime Lights Derived from Monthly Averages: 2012 to 2019. *Remote. Sens.* **13**, 922 (2021).
5. CD Elvidge, KE Baugh, M Zhizhin, FC Hsu, Why VIIRS data are superior to DMSP for mapping nighttime lights. *Proc. Asia-Pacific Adv. Netw.* **35**, 62 (2013).
6. UN, Global Sustainable Development Report, (Technical report, Division of Economic and Social Affairs.), Technical report (2016).
7. RV Phillips, MJ van der Laan, H Lee, S Gruber, Practical considerations for specifying a super learner. *Int. J. Epidemiol.* **52**, 1276–1285 (2023).
8. G Chi, H Fang, S Chatterjee, JE Blumenstock, Microestimates of wealth for all low- and middle-income countries. *Proc. Natl. Acad. Sci.* **119**, e2113658119 (2022).
9. I Permanyer, J Smits, Inequality in Human Development across the Globe. *Popul. Dev. Rev.* **46**, 583–601 (2020).
10. S Srinivasan, Disaggregating Digital Divides: Survey-based Estimates of Ownership and Use of Digital Technologies in Low- and Middle-income Countries. (2024).
11. W Bank, Living Standards Measurement Study (LSMS) (2023).
12. S Khan, A Hancioglu, Multiple Indicator Cluster Surveys: Delivering Robust Data on Children and Women across the Globe. *Stud. Fam. Plan.* **50**, 279–286 (2019).
13. A Bolgrien, EH Boyle, M Sobek, ML King, IPUMS-MICS Data Harmonization Code: Version 1.2 (2024).
14. S Kapoor, et al., REFORMS: Reporting Standards for Machine Learning Based Science (2023).
15. J Coyle, N Hejazi, I Malenica, O Sofrygin, R Phillips, Sl3: Modern Super Learning with Pipelines (Zenodo) (2021).
16. D Program, Guide to DHS Statistics - DHS-8, Technical report (2023).
